# A Ketogenic Diet for Treatment-Resistant Depression: A Randomized Clinical Trial

**DOI:** 10.1001/jamapsychiatry.2025.4431

**Published:** 2026-02-04

**Authors:** Min Gao, Megan Kirk, Heather Knight, Eva Lash, Moscho Michalopoulou, Nicola Guess, Richard Stevens, Michael Browning, Scott Weich, Philip W. J. Burnet, Susan A. Jebb, Paul Aveyard

**Affiliations:** 1 Nuffield Department of Primary Care Health Sciences, University of Oxford, Oxford, England; 2 National Institute for Health Research Oxford Health Biomedical Research Centre, Warneford Hospital, Oxford, England; 3 Department of Psychiatry, University of Oxford, Oxford, England; 4 Oxford Health NHS Foundation Trust, Oxford, England; 5 Sheffield Centre for Health and Related Research, University of Sheffield, Sheffield, England; 6 National Institute for Health Research Oxford Health Biomedical Research Centre, John Radcliffe Hospital, Oxford, England

## Abstract

**Importance:**

Preclinical evidence and case reports suggest potential therapeutic benefits of ketogenic diets (KDs) in the treatment of depression, but evidence from well-controlled randomized clinical trials (RCTs) is lacking.

**Objective:**

To assess the efficacy of a KD compared with a control diet in adults with treatment-resistant depression (TRD).

**Design, Setting, and Participants:**

This RCT was conducted between February 22 and June 15, 2024. Participants aged 18 to 65 years with TRD and a score of 15 or greater on the 9-item Patient Health Questionnaire (PHQ-9) from across the UK were included.

**Intervention:**

Participants were randomized 1:1 to one of two 6-week dietary interventions: (1) KD of prepared foods providing less than 30 g of carbohydrates per day with weekly individual dietetic support or (2) a control (phytochemical [phyto]) diet with vouchers to purchase 1 extra serving of vegetables or fruit and replace saturated fats with unsaturated fats, with equal dietetic support. The last follow-up was at 12 weeks.

**Main Outcomes and Measures:**

The primary outcome was the between-group difference in change in PHQ-9 score from baseline to week 6. Secondary outcomes included PHQ-9 score at 12 weeks, depression remission, anxiety, anhedonia, cognitive impairment, quality of life, and functional outcomes.

**Results:**

The study included 88 participants (mean [SD] age, 42.1 [13.1] years; 61 women [69%]): 44 in the KD group and 44 in the phyto diet group. Depression severity decreased markedly in both groups; the mean (SD) change in PHQ-9 score from baseline to week 6 was −10.5 (7.0) in the KD group and −8.3 (5.1) in the phyto group. The mean between-group differences in PHQ-9 score at 6 and 12 weeks were −2.18 (95% CI, −4.33 to −0.03; *P* = .05; Cohen *d*, −0.68; 95% CI −1.35 to −0.01) and −1.85 (95% CI, −4.04 to 0.33; *P* = .10; Cohen *d*, −0.58; 95% CI, −1.26 to 0.10), respectively. There were no differences in secondary outcomes between the KD and phyto groups. No serious adverse events occurred.

**Conclusions and Relevance:**

In this RCT, a KD had antidepressant benefits compared with a well-matched control diet at 6 weeks. However, the clinical relevance is uncertain, as the mean effect size compared with the control was modest and not evident in secondary analyses.

**Trial Registration:**

ClinicalTrials.gov Identifier: NCT06091163

## Key Points

**Question:**

Does a ketogenic diet (KD; high fat, low carbohydrate) vs a control diet improve mental health outcomes in individuals with treatment-resistant depression (TRD)?

**Findings:**

In this randomized clinical trial of 88 UK participants with TRD, both the KD and control groups reported rapid improvements in depression. After 6 weeks, the improvement was greater in the KD group than in the control group.

**Meaning:**

This randomized clinical trial suggests that KDs may be effective as an adjunctive treatment for TRD.

## Teaser

This randomized clinical trial assesses the effect of a ketogenic diet on mental health outcomes in individuals with treatment-resistant depression.

## Introduction

Patients with major depressive disorder are commonly treated with antidepressants, but many patients do not respond. Patients who do not respond to 2 separate agents are considered to have treatment-resistant depression (TRD).^1^

A ketogenic diet (KD), a high-fat, low-carbohydrate regimen that shifts cellular energy metabolism from glucose to ketones, has been proposed as a novel treatment for several psychiatric disorders,^2^ including depression.^3^ Ketone bodies may affect metabolic, neurotransmitter, inflammatory, and gut-brain pathways,^3,4^ which are potentially relevant to metabolic hypotheses of depression.

Research on KDs for psychiatric disorders, including major depressive disorder, is in its early stages, with evidence primarily from case reports and single-arm trials.^5,6,7,8,9,10^ Although these findings suggest that KDs are feasible and may improve severe, chronic, and refractory mental disorders, the studies were limited by small sample sizes and lack of control groups.

There are some important factors to address when developing randomized clinical trials (RCTs) of KDs for TRD.^11,12,13^ Weight loss is associated with improvements in depression and common when people adopt a ketogenic diet,^14^ and placebo effects in TRD studies are substantial, with meta-analytic data indicating a Hedges *g* value of 1.05 (95% CI, 0.91-1.10).^15^ Relying on studies without control groups is likely to overestimate the benefits of a KD. Moreover, the concentration of ketones needed to improve mental illness is uncertain.^16^ We conducted an RCT that compared a KD diet designed to maintain weight stability with a robust comparator dietary program that had reasonable face validity and matched input from a trained health coach to test whether a KD was effective in improving depression and associated symptoms.

## Methods

### Study Design

This single-blind RCT (NCT06091163) was conducted in the UK to test whether a 6-week KD was an effective treatment for TRD; the trial protocol is given in Supplement 1 and published elsewhere.^17^ The trial was conducted from February 22 to June 15, 2024. The protocol was followed as intended except dietetic support for both groups was sometimes given by trained health coaches and an extra portion of fruit was interchangeable with a portion of vegetables. Ethical approval was granted by the Oxford Research Ethics Committee. Potential participants completed an anonymous online screening questionnaire, and if eligible, they were invited to provide electronic written consent. Data collection and monitoring were managed through a trial database (REDCap). The trial followed the Consolidated Standards of Reporting Trials (CONSORT) reporting guideline.

### Setting and Participants

To be eligible for enrollment, participants had to be aged 18 to 65 years, have diagnosed depression, have received at least 2 antidepressants of adequate dosage following National Institute for Health and Care Excellence guidelines during the current episode (not necessarily concurrently), and still be experiencing at least moderately severe depression (9-item Patient Health Questionnaire [PHQ-9] score ≥15) despite ongoing treatment (eMethods in Supplement 2). The range of scores on the PHQ-9 is 0 to 27, with higher scores indicating more severe depression.

Major exclusion criteria were currently following a specific diet or unable to follow KDs (full criteria are described in the trial protocol in Supplement 1).^17^ Participants completed baseline questionnaires and provided stool and saliva samples before randomization.

### Randomization and Masking

The first 10 participants were allocated by simple randomization, and the remaining 78 participants were allocated by minimization with a random element based on body mass index (BMI) (<30 vs ≥30; calculated as weight in kilograms divided by height in meters squared) and baseline PHQ-9 score (15-19 vs 20-27). Treatment allocation (1:1) was concealed by the database from the study team until intervention groups were assigned. Participants were blinded to the nature of the other diet. The data analyst (M.G.) did not deliver the intervention.

### Procedures

#### Intervention

The intervention was delivered remotely. Participants followed a modified KD (<30 g of carbohydrates per day; 15%-20% energy from protein). To support adherence, 3 prepared KD meals per day, snacks, and urine ketone strips (Bayer Ketostix) were provided for free for 6 weeks. Participants tested first-morning urine for ketones at least twice weekly, recorded results, and reported them during weekly 30-minute counseling sessions with a dietitian or trained coach. To avoid the confounding effect of weight loss, individualized energy targets were set and snack portions adjusted if weight changed by 0.5 kg or more on 2 consecutive days. At baseline, participants also received written materials to support dietary adherence and weekly support sessions lasting up to 30 minutes with trained staff. Participants who preferred to prepare their own KD meals received £25 (US $33.36) per week in food vouchers.

#### Control

Participants followed a phytochemical (phyto) diet aimed at increasing phytochemical intake by adding 1 differently colored fruit or vegetable each day and replacing saturated animal fats with unsaturated plant oils. They received dietary support of similar frequency and duration as the KD group and followed the same protocol to maintain weight. They received food vouchers (£10/week [US $13.34/week]) to support the purchase of recommended foods. Educational materials included dietary guidelines, lists of foods categorized by color, and recipe suggestions. These minimal dietary changes, to our knowledge, have no known effect on depression and served as a credible placebo. Ketones were not measured since they would likely be undetectable and measurement would risk unblinding the purpose of the trial.

The intervention lasted 6 weeks, with 7 support sessions given to all participants. Participants were free to continue following the diets if desired, and if so, we provided them with helpful links to support them. Changes in antidepressant use as advised by the National Health Service usual care for depression were permitted in both groups.

### Data Collection

Participants self-reported demographic data, medical history, medication use, body weight, and height at baseline and completed questionnaires to assess depression (PHQ-9); anxiety (7-item Generalized Anxiety Disorder [GAD-7])^18^; anhedonia (Snaith-Hamilton Pleasure Scale [SHAPS])^19^; cognitive impairment (5-item Perceived Deficits Questionnaire [PDQ-5])^20^; quality of life (12-item Short-Form Health Survey)^21^; impairment of ability to work, conduct a normal social life, and maintain relationships (Work and Social Adjustment Scale [WSAS])^22^; and reward sensitivity (Probabilistic Instrumental Learning Task [PILT]).^23^ GAD-7 and PHQ-9 scores were collected at baseline and 2, 4, 6, and 12 weeks. All other measurements were collected at baseline and 6 and 12 weeks. Current antidepressant use was classified as monotherapy, dual therapy, or triple therapy. Sex was self-reported as man, woman, or nonbinary or other. Ethnicity was self-reported as Asian or Asian British, White, or multiple ethnicities and was included in the study to support equity, diversity, and inclusion reporting and to describe the demographic composition of the study population. This information is relevant for assessing the applicability of the findings to the ethnically diverse UK population. After completion of the study, semistructured interviews were conducted with 20 KD participants reflecting diversity in sex, ethnicity, and adherence to the diet.

### Outcomes

The primary outcome was the between-group difference in change in PHQ-9 score at 6 weeks. The secondary outcome measures were between-group differences in depression remission (defined as PHQ-9 score ≤4) and change in anxiety, anhedonia, cognitive impairment, quality of life, and impairment of ability to work, conduct a normal social life, and maintain relationships. Participants were followed up to 12 weeks to assess whether they continued the assigned diet and to evaluate changes in primary and secondary outcomes.

### Statistical Analysis

We aimed to detect a clinically meaningful 5-point difference in the PHQ-9 score.^17^ Given this, we calculated that 32 people per group would be necessary to achieve 90% power with a 5% type I error rate. Allowing for loss to follow-up of 35%, 100 participants would be needed. However, follow-up was better than this (95%), so recruitment ceased when 88 participants had been recruited and randomized.

We followed a statistical analysis plan (Supplement 1) finalized prior to database locking and conducted quantitative analyses using Stata, version 16 (StataCorp LLC) and R (version 4.4.2; ggplot2, version 3.5.1, patchwork version, 1.3.0 [R Project for Statistical Computing]). Data are presented as mean (95% CI) and reported as intention to treat, including data from all 88 randomized participants unless otherwise indicated. Participants were considered lost to follow-up if there were no available data at 6 weeks. We used linear mixed-effects regression to assess time (categorical variable), group, and group **×** time effects for each continuous outcome, adjusting for stratification factors (BMI and PHQ-9 score) to account for baseline differences between groups and participants, with a random intercept for each participant in the primary outcome analysis to account for repeated PHQ-9 measures. In secondary analyses, baseline PHQ-9 score was entered as a covariate with a random effect. Normality assumptions were checked graphically. For the binary outcome of depression remission, a mixed-effect logistic regression model was used with remission as the dependent variable and randomized group as a fixed effect and with baseline PHQ-9 score included as a random effect. Significance was defined as 2-sided *P* < .05. Our prespecified analysis assumed an exchangeable error structure; in a post hoc sensitivity analysis to verify that our findings were not sensitive to this modeling assumption, we repeated the main analysis with an autoregressive model error structure.

Participants were included in the per-protocol analysis if they completed at least 5 of 6 follow-up phone calls and were rated as adhering to the diet “completely/every day” or “mostly/most of the days” in each call. Sensitivity of the primary outcome to missing data was tested using available case analysis and scenarios assuming PHQ-9 scores of 20 or 27, baseline or last observation carried forward, or remission (score of 4).

We conducted exploratory analyses to assess the correlation (Kendall τ) between weekly ketone concentrations and changes in PHQ-9 scores to assess whether adherence to the diet was associated with improvement in depression. We evaluated whether the treatment effects on changes in PHQ-9 scores varied with additional adjustments for age, sex, and comorbidities (type 2 diabetes and hypertension).

Prespecified subgroup analyses of the primary outcome were conducted based on baseline depression severity and duration at baseline. The primary outcome model was extended to include a 3-way interaction term between study arm, time, and the subgroup variable, along with all relevant 2-way interaction terms.

Qualitative interview transcripts were analyzed using content analysis,^24^ with independent coding by 3 researchers (M.G., M.K., and M.M.) and triangulation through iterative review of field notes. We partnered with the McPin Foundation, a UK mental health research charity, to provide patient input throughout the study (eMethods in Supplement 2).

## Results

**Figure 1.**
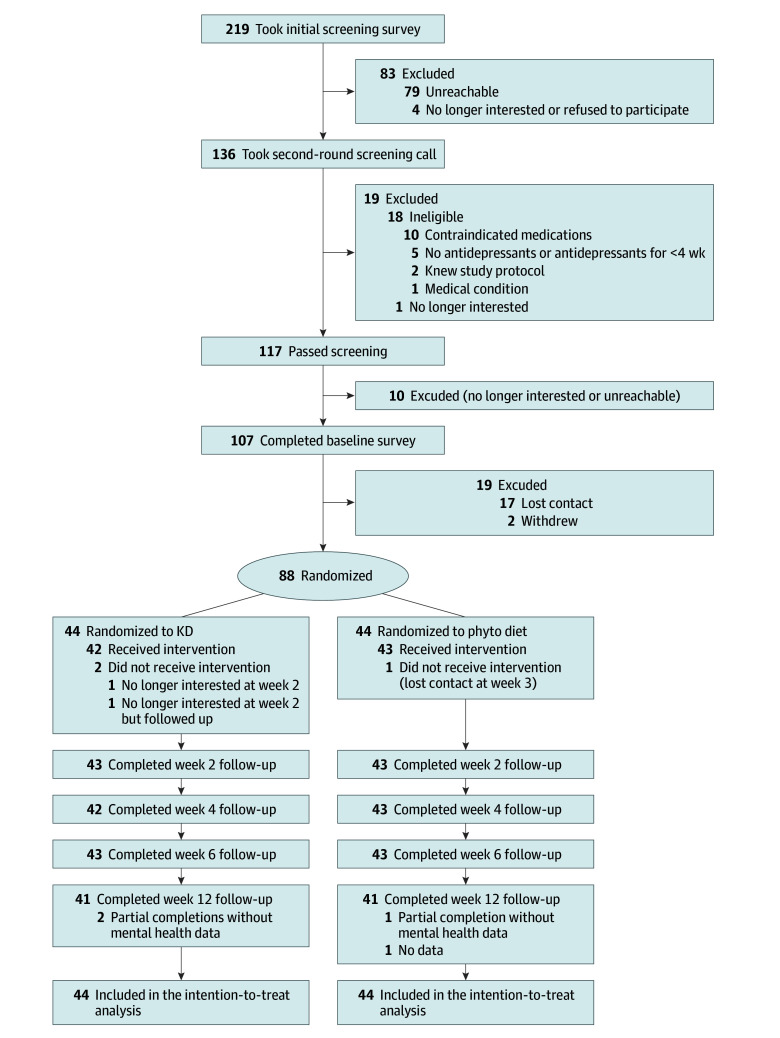
CONSORT Flowchart of Included Participants One participant missed the week 4 assessment but completed the week 6 assessment. KD indicates ketogenic diet; phyto, phytochemical.

From February 22 to June 15, 2024, 88 participants were randomized (44 to the phyto diet and 44 to the KD) (Figure 1 and eFigure 1 in Supplement 2). Data for the primary outcome were available for 86 participants (98%) at week 6 and 82 (93%) at week 12. Among the total participants, mean (SD) age was 42.1 (13.1) years; 26 participants (30%) were men, 61 (69%) were women, and 1 (1%) was nonbinary or other. Also, 7 (8%) were Asian or Asian British, 77 (88%) were White, and 4 (5%) were multiple ethnicities. At baseline, mean (SD) PHQ-9 score was 19.5 (3.2), and mean (SD) BMI was 32.3 (7.2). The median duration of the current depressive episode was 16 months (IQR, 7-30 months); 82 (93%) were receiving monotherapy. Secondary outcomes at baseline included a mean (SD) GAD-7 score of 13.3 (4.8), SHAPS score of 8.0 (3.2), PDQ-5 score of 14.4 (3.5), SF-12 score of 29.1 (2.8), and WSAS score of 27.1 (7.1) (Table 1).

**Table 1.** Baseline Characteristics of the Intention-to-Treat Population

Characteristic	Participants^a^		
	KD group (n = 44)	Phyto diet group (n = 44)	All (N = 88)
PHQ-9 score, mean (SD)	19.3 (3.2)	19.6 (3.2)	19.5 (3.2)
Secondary outcome measures, mean (SD)			
GAD-7 score	12.9 (4.6)	13.6 (5.1)	13.3 (4.8)
SHAPS score	8.1 (3.5)	7.9 (3.0)	8.0 (3.2)
PDQ-5 score	14.0 (3.3)	14.7 (3.7)	14.4 (3.5)
SF-12 score	29.0 (2.6)	29.2 (3.0)	29.1 (2.8)
WSAS score	25.5 (7.2)	28.8 (6.7)	27.1 (7.1)
Age at randomization, mean (SD), y	42.0 (13.5)	42.3 (12.8)	42.1 (13.1)
Self-reported sex^a^			
Male	12 (27)	14 (32)	26 (30)
Female	32 (73)	29 (66)	61 (69)
Nonbinary or other	0	1 (2)	1 (1)
Self-reported ethnic group			
Asian or Asian British	6 (14)	1 (2)	7 (8)
White	35 (80)	42 (95)	77 (88)
Multiple ethnic groups	3 (7)	1 (2)	4 (4)
Educational level			
Some education (no qualification)	1 (2)	3 (7)	4 (4)
GCSE/O level or equivalent	5 (11)	6 (14)	11 (13)
A level/BTEC or equivalent	9 (20)	7 (16)	16 (18)
Undergraduate or postgraduate degree	29 (66)	28 (64)	57 (65)
IMD quintile			
1 (Most deprivation)	9 (20)	8 (18)	17 (19)
2	4 (9)	7 (16)	11 (13)
3	13 (30)	8 (18)	21 (24)
4	10 (23)	11 (25)	21 (24)
5 (Least deprivation)	8 (18)	10 (23)	18 (20)
BMI			
Mean (SD)	32.0 (6.1)	32.6 (8.1)	32.3 (7.2)
18.5 to <25	6 (14)	8 (18)	14 (16)
25 to <30	12 (27)	10 (23)	22 (25)
30 to <35	11 (25)	8 (18)	19 (22)
≥35	15 (34)	18 (41)	33 (38)
Smoking status			
Nonsmoker	41 (93)	41 (93)	82 (93)
Smoker	3 (7)	3 (7)	6 (7)
Alcohol consumption, AUDIT-C score, mean (SD)	1.6 (1.6)	1.6 (1.5)	1.6 (1.5)
Duration of current depressive episode, median (IQR), mo	14 (6-30)	20 (8-36)	16 (7-30)
Current antidepressant medication use			
Monotherapy	41 (93)	41 (93)	82 (93)
Dual therapy	3 (7)	2 (5)	5 (6)
Triple therapy	0	1 (2)	1 (1)
Current antidepressant medication type			
SSRI	26 (59)	20 (46)	46 (52)
Serotonin modulator and stimulator	1 (2)	0	1 (1)
Serotonin and norepinephrine reuptake inhibitor	8 (18)	16 (36)	24 (27)
Tetracyclic antidepressants	6 (14)	6 (14)	12 (14)
Ketamine	0	1 (2)	1 (1)
Antipsychotics	1 (2)	0	1 (1)
Tricyclic antidepressants	2 (5)	1 (2)	3 (3)
Illicit drug use^b^			
Never	42 (96)	44 (100)	86 (98)
Monthly or less	2 (4)	0	2 (2)
Diabetes^b^			
No	41 (93)	42 (96)	83 (94)
Type 1	0	0	0
Type 2	1 (2)	2 (4)	3 (3)
Other	2 (4)	0	2 (2)
Hypertension			
No	39 (89)	40 (91)	79 (90)
Yes	5 (11)	4 (9)	9 (10)
No cardiovascular disease^b^	44 (100)	44 (100)	88 (100)

Abbreviations: A level, advanced level; AUDIT-C, Alcohol Use Disorders Identification Test–Consumption; BMI, body mass index (calculated as weight in kilograms divided by height in meters squared); BTEC, Business and Technology Education Council; GAD-7, 7-item General Anxiety Disorder Scale; GCSE, General Certificate of Secondary Education; IMD, index of multiple deprivation; KD, ketogenic diet; O level, ordinary level; PDQ-5, 5-item, Perceived Deficits Questionnaire; PHQ-9, 9-item Patient Health Questionnaire; phyto, phytochemical; SF-12, Short-Form 12 Health Survey; SHAPS, Snaith-Hamilton Pleasure Scale; SSRI, selective serotonin reuptake inhibitor; WSAS, Work and Social Adjustment Scale.

^a^ Data are presented as number (percentage) of participants unless otherwise indicated.

^b^ Omitted if no data on other options.

**Figure 2.**
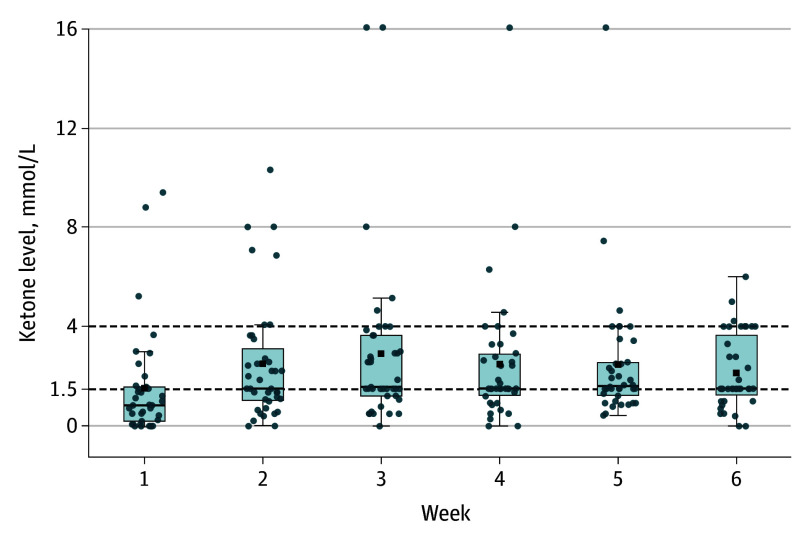
Box Plot of Weekly Ketone Concentration in the Ketogenic Diet Group Black squares indicate the distribution of ketone readings; lines in the middle of boxes, median level; outer ends of the boxes, IQRs; error bars, ranges; blue dots, ketone readings; and dashed lines, ketone levels of 1.5 mmol/L and 4.0 mmol/L.

Two KD participants (5%) discontinued the intervention early; 1 of these participants provided follow-up data. One participant in the phyto group discontinued the diet. At least 84% of participants in the KD group measured urine ketones at least twice weekly (as instructed), while at least 50% measured them daily (eFigure 2 in Supplement 2). Twenty-eight participants (64%) maintained a ketone concentration of 1.5 mmol/L or greater at least 60% of the time, with the proportion doing so increasing over time (Figure 2).

### Effect on Depression

**Figure 3.**
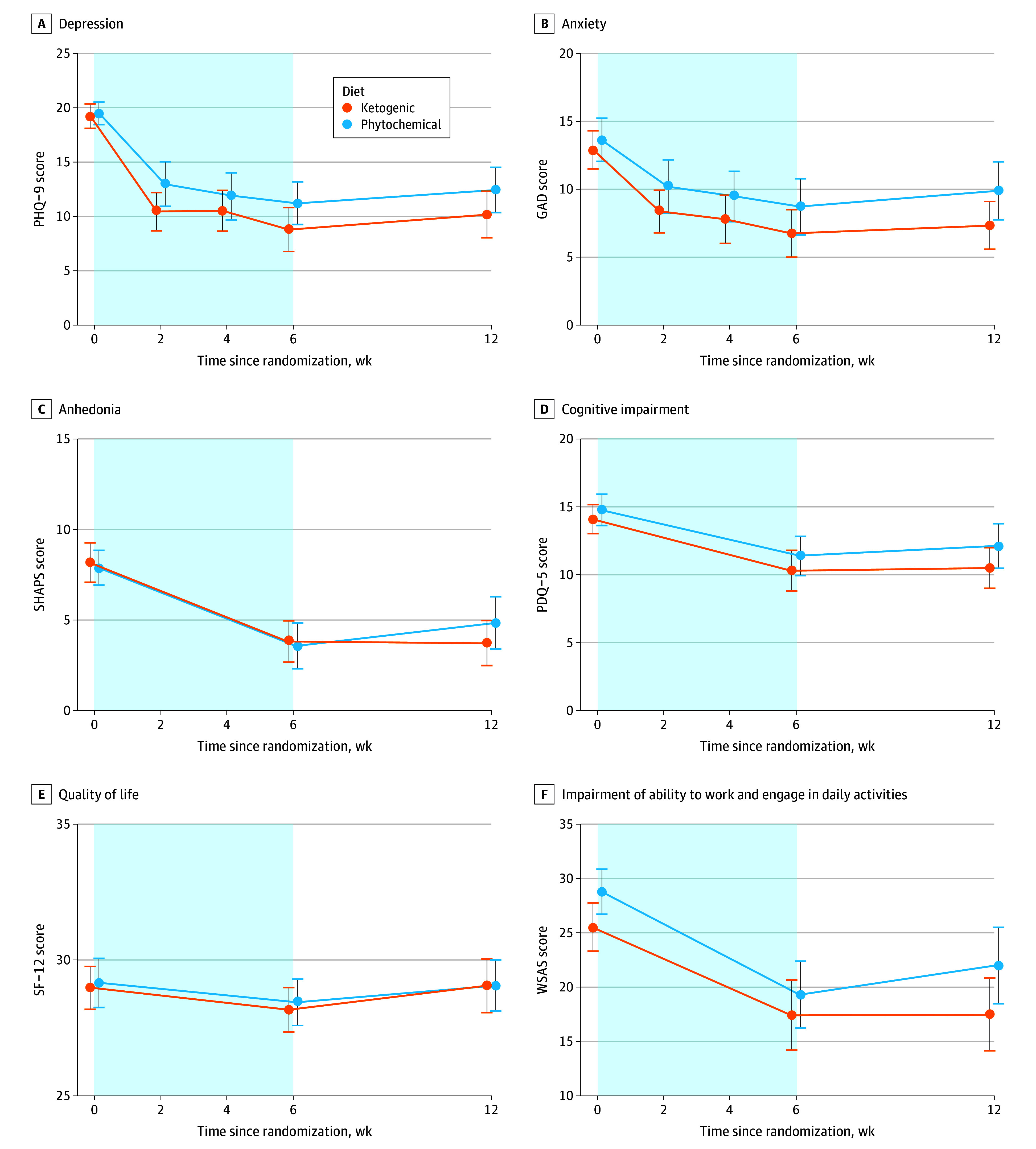
Line Graphs of Changes in Primary and Secondary Outcome Scores Over 12 Weeks GAD indicates 7-item General Anxiety Disorder Scale; PDQ-5, 5-item Perceived Deficits Questionnaire; PHQ-9, 9-item Patient Health Questionnaire; SF-12, Short-Form 12 Health Survey; SHAPS, Snaith-Hamilton Pleasure Scale; WSAS, Work and Social Adjustment Scale.

The mean (SD) change in PHQ-9 score from baseline to week 6 (primary outcome) was −10.5 (7.0) in the KD group and −8.3 (5.1) in the phyto group, representing a treatment effect of −2.18 (95% CI, −4.33 to −0.03; *P* = .05; standardized mean difference [SMD], −0.68; 95% CI, −1.35 to −0.01). At week 12, there was no difference between groups (−1.85; 95% CI, −4.04 to 0.33; *P* = .10; SMD, −0.58; 95% CI, −1.26 to 0.10) (Figure 3 and eTable 1 in Supplement 2).

Sixty-one participants (34 in the KD group, 27 in the phyto group) met the criteria for the per-protocol analysis (eFigure 2 in Supplement 2). There was no between-group difference in PHQ-9 score at week 6 (difference, −2.81; 95% CI, −6.08 to 0.45; *P* = .09; SMD, −0.88; 95% CI, −1.89 to 0.14) or week 12 (difference, −1.84; 95% CI, −5.17 to 1.48; *P* = .27; SMD, −0.57; 95% CI, −1.61 to 0.46) (eTable 1 in Supplement 2).

Sensitivity analyses assessing the impact of missing data at week 2 (n = 2), week 4 (n = 3), week 6 (n = 2), and week 12 (n = 6) using 5 imputation strategies (eMethods in Supplement 2) showed minimal differences from the primary analysis (eTable 2 in Supplement 2). The treatment effects on changes in PHQ-9 scores did not vary with additional adjustments for age, sex, or comorbidities (type 2 diabetes, hypertension) (eTable 3 in Supplement 2). In a post hoc sensitivity analysis using an alternative error model, similar results were obtained, but the effect on PHQ-9 score at 6 weeks was no longer significant.

Subgroup analyses (eTable 4 in Supplement 2) showed that the response to the KD was greater in participants with severe depression (PHQ-9 score, 20-27) than in those with moderate depression (PHQ-9, 15-19). At week 6, the difference in PHQ-9 score between groups was −4.73 (95% CI, −8.16 to −1.30) for participants with severe depression and 0.16 (95% CI, −2.30 to 2.63) for those with moderately severe depression (*P* = .02 for the difference in treatment effects). At week 12, the differences were −5.18 (95% CI, −8.63 to −1.72) and 1.24 (95% CI, −1.27 to 3.78), respectively.

At 6 weeks, 11 patients in the KD group (25%) and 4 patients in the phyto group (9%) achieved remission from depression (PHQ-9 score ≤4). At 12 weeks, 3 patients in the KD group (7%) had experienced relapse, with 8 patients continuing to experience remission (18%); remission in the phyto group was unchanged (4 patients [9%]). There was no significant difference in depression remission between groups (eTable 5 in Supplement 2).

### Effect on Other Secondary Outcomes

No significant differences were observed between the groups for GAD-7, SHAPS, PDQ-5, SF-12, or WSAS scores (Figure 3 and eTable 5 in Supplement 2) except for anxiety at week 12. The between-group difference in GAD-7 score at week 12 was −2.02 (95% CI, −3.95 to −0.10; *P* = .04; SMD, −0.42; 95% CI −0.82 to −0.02).

### Process Outcomes

Participants in the KD group attended a mean (SD) of 92% (18%) of support sessions compared with 85% (20%) in the phyto group (Table 2). By week 12, 6 weeks after support ended, 21 participants (48%) reported fully discontinuing the KD and only 9 participants (20%) followed the diet at least half the days. A total of 21 participants in the phyto diet group (48%) reported continuing more than half the time. Exploratory post hoc analyses examined the association between ketosis concentration and depression severity (eFigures 3-5 in Supplement 2). Kendall τ-b correlations showed no significant association between ketone concentration and PHQ-9 score. Participants with mean ketone levels of 1.5 mmol/L or greater (n = 23) had a mean (SD) change in PHQ-9 score of −8.4 (7.0), and those reaching mean ketone levels of 4 mmol/L or greater (n = 5) had a mean (SD) change of −9.0 (9.7). By contrast, participants with mean ketone levels lower than 1.5 mmol/L (n = 20) showed a greater mean (SD) change in PHQ-9 score of −12.9 (6.3), and those with mean ketone levels lower than 4 mmol/L (n = 38) had a mean (SD) change of −10.7 (6.7). Mechanistic analyses of cortisol, gut microbiome, reward sensitivity, and anhedonia will be reported separately following the statistical analysis plan (Supplement 1).

**Table 2.** Process Outcomes in Participants Assigned to the KD Group or Phyto Diet Group

Outcome	Participants		
	KD group (n = 44)	Phyto diet group (n = 44)	Total (N = 88)
Proportion who completed sessions out of 6 sessions, %, mean (SD)	92 (18)	85 (20)	88 (19)
Reported dietary adherence during each call, No. (%)^a^			
Week 1	43 (98)	34 (77)	77 (88)
Week 2	38 (86)	39 (89)	77 (88)
Week 3	38 (86)	38 (86)	76 (86)
Week 4	35 (80)	31 (70)	66 (75)
Week 5	34 (77)	36 (82)	70 (80)
Week 6	34 (77)	35 (80)	69 (78)
Diet adherence at week 12, No. (%)			
Not at all	21 (48)	2 (5)	23 (26)
Few days a week	13 (30)	19 (43)	32 (36)
More than half the days in a week	5 (11)	10 (23)	15 (17)
Nearly every day	4 (9)	11 (25)	15 (17)
Satisfaction with the intervention			
I have enjoyed the food I have eaten over the past 6 wk, mean (SD), score^b^	3.70 (1.04)	4.28 (0.88)	3.99 (1.00)
I feel that the diet has improved my mental health, mean (SD), score^b^^,^^c^	3.62 (1.01)	3.74 (0.66)	3.68 (0.85)
Don’t know, No. (%)^d^	1 (2)	0	1 (1)
Overall, how was your experience of the diet and support you got, mean (SD), score^e^	4.44 (0.85)	4.86 (0.35)	4.65 (0.68)

Abbreviations: KD, ketogenic diet; phyto, phytochemical.

^a^ Health coaches selected “completely/every day” or “mostly/most of the days” when assessing participants’ adherence to the diet.

^b^ A score of 1 indicated strongly disagree; 5, strongly agree; and 6, don’t know.

^c^ Mean values of responses without including “don’t know.”

^d^ Responses of “don’t know” were reported separately.

^e^ A score of 1 indicated very poor; 5, very good; and 6, don’t know.

No serious events related to the research procedures occurred. Results of exploratory analyses are described in the eFigure 6 in Supplement 2.

### Qualitative Interview Results

Participant demographics are shown in eTables 7 and 8 in Supplement 2, and qualitative data synthesis is shown in eTable 9 in Supplement 2. The eMethods in Supplement 2 detail participants’ motivations and feedback on the intervention.

## Discussion

This RCT provides preliminary evidence that adherence to a KD may have small antidepressant benefits in people with TRD. There was no benefit for anxiety, cognition, or functional measures. Most people sustained ketosis most of the time during the intensive support phase, but adherence was challenging, and once the provision of KD meals and support ended, only 9% of participants reported diet continuation. The per-protocol analysis did not suggest a larger effect in those who were adherent. Evidence of improvement persisted in different analyses but with wide 95% CIs, and ketone concentrations were not associated with depression improvement. There was exploratory evidence that the treatment effect was substantially larger in people with severe depression, with no benefit in those with moderate depression. Qualitative evidence highlighted the demanding nature of KDs and logistical burdens (eg, meal planning, social restrictions) and emphasized the importance of external support.

Previous human studies^5,6,7,8,9,10^ on KDs were limited to small, uncontrolled designs with high attrition. Our controlled design with a high completion rate in a heterogeneous population provides, to our knowledge, the first evidence of modest short-term antidepressant effects. The observed PHQ-9 score reduction (−2.2 points) in the KD group compared with the phyto group was lower than the prespecified minimal clinically important difference of 5 points, but other studies have suggested that a change of −1.7 points in PHQ-9 score may be used as a threshold for improvement.^25^ A meta-analysis showed that antipsychotic augmentation in TRD yielded a mean placebo-adjusted reduction in PHQ-9 score of about 3.^26^ This finding suggests that the effect size for KD, while statistically marginal, might be clinically nontrivial in the context of TRD.

Recent reviews have suggested a potential benefit of dietary interventions for depression.^27,28^ However, our phyto diet was unlikely to be sufficient in influencing depression outcomes. A recent systematic review including 13 RCTs and 1 meta-analysis found that specific plant-based compounds (eg, saffron, lavender, and turmeric) had potential efficacy in depression,^29^ but the quality of evidence was poor and the phytonutrients reviewed were not part of our intervention. The absolute reduction in PHQ-9 score in the phyto group in our study was 8 points, suggesting that support and belief in the dietary program may have had an effect that was larger than any improvement specifically attributable to the KD.

Our trial showed that the KD did not improve other assessed mental health outcomes except for a transient effect on anxiety. While preclinical research showed that exogenous ketone supplementation reduced anxiety behaviors in rats^30^ and case reports of KD addressing anxiety described 2 people with reduced anxiety symptoms, 1 with bipolar I disorder and another with unspecified mood disorder,^31,32^ our RCT suggests that benefits on anxiety are minimal.

Our trial was designed to assess efficacy by providing intensive support and supplementary food to maximize adherence to a KD, and we found that when all support stopped, few people continued the KD. Notably, participants did not experience relapse to previous levels of depression, suggesting either that a short period of carefully supported dietary intervention may have enduring benefits or that the mechanism of any improvement was unrelated to ketosis and perhaps linked to study participation. We found that participants with long-lasting TRD who had not responded to current treatment offered by primary and secondary care had large reductions in PHQ-9 score (KD: mean [SD], −10.5 [7.0]; phyto diet: mean [SD], −8.3 [5.1]), which may reflect the benefit of providing hope and expectation of improvement. The interventions differed in the type of diet but also in the need to prepare food and involved a larger cash transfer to the KD arm than the phyto arm. There is no evidence that differences in diet design or the provision of cash transfers are effective treatments for depression; therefore, these aspects of the intervention are unlikely to have confounded the observed effect of the KD.

### Strengths and Limitations

Our study evaluated the therapeutic effects of a KD for depression and has several strengths. First, inclusive recruitment strategies resulted in a participant pool of a broad geographic and socioeconomically diverse UK population. Second, the controlled design addressed a major limitation of previous studies by mitigating the placebo effects common in the treatment of depression and the impact of additional therapist contact. Third, we addressed confounding factors such as weight loss by providing advice and support to maintain weight, with no significant weight changes observed. Fourth, prepared KD meals and ketone monitoring aimed to support adherence and enabled exploratory analysis between ketone levels and depression changes. Fifth, the follow-up rate was high, reducing the uncertainties introduced by missing data.

This study also has limitations. The trial had a short intervention period (6 weeks), which is common in dietary interventions to assess initial efficacy, particularly in populations with complex conditions like TRD. The efficacy of KD for epilepsy develops gradually over a period of 1 to 3 weeks.^33^ In this study, the PHQ-9 score reduction started from 2 weeks and persisted to 6 weeks, indicating that this duration was sufficient to induce measurable short-term effects. We aimed to match placebo effects, and the health coaches reported that both groups expressed belief in the efficacy of the intervention; however, we cannot be certain that they were matched. Also, the provision of prepared meals in the KD arm reduced shopping and preparation demands, which may have influenced mood through convenience independent of dietary composition. In addition, using a single morning urine sample twice weekly provides only limited information on ketone levels and is less precise than measuring plasma ketone concentrations. While serious adverse events were monitored, the duration was too short to be likely to assess common long-term adverse effects of KDs (eg, hyperlipidemia, kidney stones, and vitamin deficiencies). Furthermore, we did not capture any changes in antidepressant use and metabolic biomarkers during the study. Finally, while participants reported moderately severe symptoms of depression and a relatively long current episode duration, the degree to which the participants recruited into this remote study represent those seen in routine clinical practice is uncertain.

## Conclusions

This RCT found small improvements in depression in people with TRD when a KD was used as an adjunct to pharmacotherapy. However, adherence to the diet required intense support, and few patients chose to continue the diet after support withdrawal. Further work to develop a more comprehensive intervention is needed before further clinical testing.

## Notes

Supplement 1.
Trial Protocol and Statistical Analysis Plan

Supplement 2.
eMethods.
**eFigure 1.** Geographic spread of participants enrolled in the DIME Study based on targeted recruitment **eFigure 2.** The percentage of participants in the KD group measuring urine ketones each week **eFigure 3.** Association between mean values of ketone concentration and change in PHQ-9 score from baseline to week 6 **eFigure 4.** Association between the proportion of mean values of ketone concentration above 1.5 mmol/L with change in PHQ-9 score from baseline to week 6 **eFigure 5.** Association between the proportion of mean values of ketone concentration above 4 mmol/L with change in PHQ-9 score from baseline to week 6 **eTable 1.** Primary outcome by group allocation and per protocol **eTable 2.** Sensitivity analysis outcomes by group allocation at week 6, of participants assigned to the KD group (n=44) or Phyto group (n=44) **eTable 3**. Secondary analyses by group allocation with adjustments for age, sex and presence of comorbidities (T2DM, hypertension), of participants assigned to the KD group (n=44) or Phyto group (n=44) **eTable 4.** Subgroup analysis outcomes by baseline depression severity (PHQ-9 scores of 15-19 vs. 20-27) and depression duration at baseline (median and above, vs. below median) **eTable 5.** Secondary outcomes by group allocation, of participants assigned to the KD group (n=44) or Phyto group (n=44) **eTable 6.** Exploratory outcomes by group allocation, of participants assigned to the KD group (n=44) or Phyto group (n=44) **eTable 7.** Demographics of 20 participants who participated in qualitative interviews **eTable 8.** Individual-level demographics of participants who participated in qualitative interviews (*n* = 20) **eTable 9.** Qualitative evidence synthesis from content analysis: Categories and data relating to perceptions and experiences of the intervention programme

Supplement 3.
Data Sharing Statement
