# Supplementary material for: A Ketogenic Diet for Treatment-Resistant Depression: A Randomized Clinical Trial
Source: JAMA Psychiatry. 2026 Feb 4;83(4):331–40. doi: 10.1001/jamapsychiatry.2025.4431 (PMC12874075; doi:10.1001/jamapsychiatry.2025.4431)
Supplement: Supplement 1. — Trial Protocol and Statistical Analysis Plan [file jamapsychiatry-e254431-s001.pdf]

Central University Research Ethics Committee (CUREC)

CUREC 3 Protocol and Application Form

Pharmacological Studies in Healthy Volunteers

Only fully signed type-written applications will be accepted, by email

Please complete this Protocol and application form if your study involves the administration of a licensed drug, herbal remedy, or food supplement to healthy volunteers AND is not a clinical trial.

All advisory text is highlighted in yellow and should be deleted before finalising the document.
Should you require any assistance in completing this document, please contact the MS IDREC Secretariat - ethics@medsci.ox.ac.uk.

Section A. Research details

**1. Full title of research**	**Dietary Interventions for MEntal Health in People with Treatment-Resistant Depression**	
**2. Short title of research**	Dietary Interventions for Mental Health (DIME)	
**3. MS IDREC reference**		
**4. Date and version number**	30 May 2023, V0.1	
**5. Principal Investigator (PI)**	Dr. Min Gao	
**6. PI's training in research ethics and/or research integrity**<br>Research integrity training within the past 3 years is compulsory for all University research staff and students. Please enter date of relevant course completion (one of 1a, 1b or 1c must be completed).	**Course Title**	**Date completed**
	1a. Research Integrity Core Course (New researchers & students)	
	1b. Research Integrity Refresher Course (Experienced researchers)	18/05/2023
	1c. Other (please specify title)	NIHR GCP 09/02/2022
	2. Supplementary Module – Research involving human participants	
	3. Information Security Training	13/02/2023
**7. Student name and degree programme (if applicable)**	N/A	
**8. Department/Institute name**	Nuffield department of Primary Care Health<br>University of Oxford	

**9. University email address**	min.gao@phc.ox.ac.uk
**10. University telephone number**	+44 (0)1865 289315
**11. Medically qualified collaborator (Licensed doctor)**	Professor Paul Aveyard, GP, University of Oxford, GMC: 3332407<br>01865 617 860, paul.aveyard@phc.ox.ac.uk
**12. Funding source**	National Institute of Health Research (NIHR) Oxford Health Biomedical Research Centre (BRC)

**13. Will you submit or have you submitted this research to another ethics committee?**	Yes ☐	No ☒
If other relevant approvals for this research are required (e.g. from other universities' ethics committees) please attach them and give more details below:		

**14. State any conflicts of interest and explain how these will be addressed**	None
**15. Confidentiality Statement**	This document contains confidential information that must not be disclosed to anyone other than the authorised individuals from the University of Oxford, the Investigator Team and members of the Medical Sciences Interdivisional Research Ethics Committee (MS IDREC), unless authorised to do so.

Section B. Researchers

**1. Researcher title and name**	Professor Paul Aveyard	
**2. Department / Institute name**	Nuffield Department of Primary Care Health Sciences<br>University of Oxford	
**3. Role in research**	Professor Paul Aveyard serves as the co-PI on this study. He will supervise throughout this study.	
**4. Training in research ethics and/or research integrity**<br>Research integrity training within the past 3 years is compulsory for all University research staff and students. Please enter date of relevant	**Course Title**	**Date completed**
	1a. Research Integrity Core Course (New researchers & students)	
	1b. Research Integrity Refresher Course (Experienced researchers)	05/08/2022
	1c. Other (e.g. GCP - please specify title)	GCP 29/12/2020

course completion (one of 1a, 1b or 1c must be completed).	2. Supplementary Module – Research involving human participants	05/08/2022
	3. Information Security Training	23/03/2023

**1. Researcher title and name**	Dr. Megan Kirk Chang	
**2. Department / Institute name**	Nuffield Department of Primary Care Health Sciences<br>University of Oxford	
**3. Role in research**	Dr. Megan Kirk Chang will work closely on this study. She will work with Dr. Min Gao on study management. She will prepare materials for participant recruitment and develop participant-facing materials.	
**4. Training in research ethics and/or research integrity**<br>Research integrity training within the past 3 years is compulsory for all University research staff and students. Please enter date of relevant course completion (one of 1a, 1b or 1c must be completed).	**Course Title**	**Date completed**
	1a. Research Integrity Core Course (New researchers & students)	07/03/2023
	1b. Research Integrity Refresher Course (Experienced researchers)	
	1c. Other (e.g. GCP – please specify title)	NIHR GCP<br>13/02/2023
	2. Supplementary Module – Research involving human participants	24/05/2023
	3. Information Security Training	02/02/2023

**1. Researcher title and name**	Dr. Nicola Guess	
**2. Department / Institute name**	Nuffield Department of Primary Care Health Sciences<br>University of Oxford	
**3. Role in research**	Dr. Nicola Guess, a dietitian, will choose the ketogenic meals/snacks and support the development of related dietary materials for this study.	
**4. Training in research ethics and/or research integrity**<br>Research integrity training within the past 3 years is compulsory for all University research staff and students. Please enter date of relevant course completion (one of 1a, 1b or 1c must be completed).	**Course Title**	**Date completed**
	1a. Research Integrity Core Course (New researchers & students)	
	1b. Research Integrity Refresher Course (Experienced researchers)	16th May 2022
	1c. Other (e.g. GCP – please specify title)	2nd May 2022
	2. Supplementary Module – Research involving human participants	29/06/23
	3. Information Security Training	29/06/23

**1. Researcher title and name**	Professor Michael Browning		
**2. Department / Institute name**	Department of Psychiatry<br>University of Oxford		
**3. Role in research**	Professor Michael Browning will provide support for conducting research in individuals with depression.		
**4. Training in research ethics and/or research integrity**<br>Research integrity training within the past 3 years is compulsory for all University research staff and students. Please enter date of relevant course completion (one of 1a, 1b or 1c must be completed).	**Course Title**	**Date completed**	
	1a. Research Integrity Core Course (New researchers & students)		
	1b. Research Integrity Refresher Course (Experienced researchers)	June 2022	
	1c. Other (e.g. GCP – please specify title)	NIHR GCP April 2022	
	2. Supplementary Module – Research involving human participants		
	3. Information Security Training	June 2023	

**1. Researcher title and name**	Dr. Moscho Michalopoulou	
**2. Department / Institute name**	Nuffield Department of Primary Care Health Sciences<br>University of Oxford	
**3. Role in research**	Dr. Moscho Michalopoulou, a dietitian, will work with Dr. Nicola Guess to offer support in the design of dietary interventions and related dietary materials for this study.	
**4. Training in research ethics and/or research integrity**<br>Research integrity training within the past 3 years is compulsory for all University research staff and students. Please enter date of relevant course completion (one of 1a, 1b or 1c must be completed).	**Course Title**	**Date completed**
	1a. Research Integrity Core Course (New researchers & students)	
	1b. Research Integrity Refresher Course (Experienced researchers)	29/05/2023
	1c. Other (e.g. GCP – please specify title)	NIHR GCP May 2023
	2. Supplementary Module – Research involving human participants	29/05/2023
	3. Information Security Training	29/05/2023

**1. Researcher title and name**	Professor Scott Weich

**2. Department / Institute name**	School of Health and Related Research<br>University of Sheffield	
**3. Role in research**	Professor Scott Weich will provide support for conducting research in individuals with depression.	
**4. Training in research ethics and/or research integrity**<br>Research integrity training within the past 3 years is compulsory for all University research staff and students. Please enter date of relevant course completion (one of 1a, 1b or 1c must be completed).	**Course Title**	**Date completed**
	1a. Research Integrity Core Course (New researchers & students)	
	1b. Research Integrity Refresher Course (Experienced researchers)	31/07/2020
	1c. Other (e.g. GCP – please specify title)	GCP 31/07/2020
	2. Supplementary Module – Research involving human participants	
	3. Information Security Training	01/09/23

**1. Researcher title and name**	Dr. Philip Burnet	
**2. Department / Institute name**	Department of Psychiatry<br>University of Oxford	
**3. Role in research**	Dr. Philip Burnet will provide support for collecting stool samples and conducting gut microbiome analysis.	
**4. Training in research ethics and/or research integrity**<br>Research integrity training within the past 3 years is compulsory for all University research staff and students. Please enter date of relevant course completion (one of 1a, 1b or 1c must be completed).	**Course Title**	**Date completed**
	1a. Research Integrity Core Course (New researchers & students)	
	1b. Research Integrity Refresher Course (Experienced researchers)	June 2022
	1c. Other (e.g. GCP – please specify title)	
	2. Supplementary Module – Research involving human participants	
	3. Information Security Training	June 2022

**1. Researcher title and name**	Professor Susan Jebb

**2. Department / Institute name**	Nuffield Department of Primary Care Health Sciences<br>University of Oxford	
**3. Role in research**	Professor Susan Jebb will provide advice on the design of this study.	
**4. Training in research ethics and/or research integrity**<br>Research integrity training within the past 3 years is compulsory for all University research staff and students. Please enter date of relevant course completion (one of 1a, 1b or 1c must be completed).	**Course Title**	**Date completed**
	1a. Research Integrity Core Course (New researchers & students)	04/04/2022
	1b. Research Integrity Refresher Course (Experienced researchers)	
	1c. Other (e.g. GCP – please specify title)	NIHR GCP 16/01/2023
	2. Supplementary Module – Research involving human participants	
	3. Information Security Training	30/03/23

**1. Researcher title and name**	Dr. Richard Stevens	
**2. Department / Institute name**	Nuffield Department of Primary Care Health Sciences<br>University of Oxford	
**3. Role in research**	Dr Richard Stevens will provide support for data analysis throughout the study.	
**4. Training in research ethics and/or research integrity**<br>Research integrity training within the past 3 years is compulsory for all University research staff and students. Please enter date of relevant course completion (one of 1a, 1b or 1c must be completed).	**Course Title**	**Date completed**
	1a. Research Integrity Core Course (New researchers & students)	
	1b. Research Integrity Refresher Course (Experienced researchers)	22/12/2022
	1c. Other (e.g. GCP – please specify title)	
	2. Supplementary Module – Research involving human participants	
	3. Information Security Training	30/03/2023

Section C. synopsis		
**1. Please state why this research is not considered a Clinical Trial of an Investigative Medicinal Product**	This study is not investigating a medicinal product. The intervention involves a ketogenic diet (KD - i.e. a high-fat and very low carbohydrate diet) which is followed in the population without medical supervision. In our study, the diet intervention includes pre-prepared meals and snacks, which are considered food rather than medicinal products and are not subject to regulatory oversight as such. These products are produced by approved manufacturers.	
**2. List all places where research will be conducted**	Online and over the phone	
**3. Age range of participants**	18 – 65 years old<br><br>As an early stage, proof of concept investigation of a novel approach to the treatment of depression, we consider the restricted age-range is justified for two principal reasons. Crucially, people aged over 65 are seen by old-age psychiatrists rather than in 'routine' psychiatric services, and we plan for future trials of this intervention fitting into routine psychiatric services, not the specialist old-age services. Second, older people are more likely to have multi-morbidity and to be taking prescription medications to manage their conditions. This increases the risk of unforeseen interactions with the diet and might make it harder to detect and isolate the effect of the dietary treatment if medications are altered concomitantly.	
**4. Anticipated number of participants**	100 participants (50 ketogenic diet [KD] group; 50 control group).	
**5. Anticipated research start date**	1st September 2023 or as soon thereafter as practical	
**6. Anticipated research end date**	31st December 2024	
**7.**	**Objectives**	**Outcome Measures**
**Primary**	To assess the effect of a KD diet versus a control group on self-reported depression severity at week 6	Difference between control group and intervention group in self-reported depression severity assessed using the Patient Health Questionnaire-9 (PHQ-9)
**8. Name of drug/substance**	Pre-prepared ketogenic diet meals (3 meals with snacks per day) with weekly nutritional counselling with a registered dietitian	
**9. Purpose of drug/substance use in this research**	To assess the effect of a KD diet versus a control group on self-reported depression severity	
**10. Adverse reactions and side effects posing a**	Previous studies of the intervention have reported mostly mild and transient side effects that may appear two to seven days after starting a ketogenic diet, including	

**particular risk with this drug/substance**	headache, foggy brain, fatigue, irritability, nausea, difficulty sleeping, and constipation. These symptoms usually disappear by themselves within a few days to a week.

Section D. Abbreviations

Define all unusual or 'technical' terms related to the research. Add or delete rows as appropriate. Maintain alphabetical order for ease of reference.

AUDIT	Alcohol Use Disorders Identification Test
BMI	Body mass index
BRC	Biomedical Research Centre
CI	Chief Investigator
CRF	Case Report Form
CTU	Clinical Trials unit
eCRF	electronic Case Report Form
eICF	electronic Informed Consent Form
ePIS	electronic participant information sheet
GAD-7	General Anxiety Disorder Scale
GCP	Good Clinical Practice
GP	General Practitioner
ICF	Informed Consent Form
KD	Ketogenic diet
MS IDREC	Medical Sciences Interdivisional Research Ethics Committee
NIHR	National Institute of Health Research
PDQ-5	Perceived deficits questionnaire – 5-item
PHQ	Patient Health Questionnaire
PI	Principal investigator
PILT	Probabilistic instrumental learning task
RA	Research assistant
SCFAs	Short-chain fatty acids
SF-12	Short-Form 12 Health Survey
SHAPS	Snaith-Hamilton Pleasure Scale
SLGT2	Sodium-glucose co-transporter-2
WSAS	Work and Social Adjustment Scale

Section E. Background and rationale

One in 6 people suffer from clinical depression. Antidepressants are a first-line treatment for depression, but at least 1 in 3 patients with depression do not respond to antidepressants and not everyone responds to, or wants psychological therapy. Furthermore, there is still a high relapse rate after psychological treatment.[1] Longer depressive episodes, a higher number of hospitalisations, moderate to high suicide risk, physical and psychiatric comorbidities, and higher dosage of medication,[2] were partly explained by the low response (16-17%) and remission rates (13%).[3] Hence, we need new treatment options.

Recent research in depression indicates that the imbalance between excitatory (e.g. glutamatergic system) and inhibitory (e.g. GABA system) systems may be a better way of understanding depression.[4] There is strong evidence that GABAergic neurotransmission is disrupted in depression and concentrations of GABA are significantly reduced in depression, especially in treatment-resistant depression.[5] This suggests increasing inhibitory neurotransmitters (e.g. GABA) could be an alternative way to treat depression. Treatment-resistant depression is frequent in clinical practice, but is less studied than other forms of depression. Therefore, investigation into treatment-resistant depression is public health relevant.

KDs (ketogenic diets) have been recently suggested as a possible intervention for depression. A KD is characterised by high-fat and very low-carbohydrate intake, and has been used to treat refractory epilepsy and metabolic disorders that could occur with depression.[6] KDs lead to the production of ketone bodies from fatty acids, which provide brain cells with an energy source that is more efficient than glucose, resulting in beneficial metabolic changes in the body and brain.[7] Ketone bodies are also believed to exert anti-seizure effects by interfering with cell metabolism, homeostasis and signalling.[8] Ketogenic diets (KDs) have been widely used to effectively treat refractory epilepsy since the 1920s, reducing seizure frequency by at least half. As depression and epilepsy are highly interrelated, KDs may have therapeutic effects on depression that need to be estimated.

Preclinical data, mainly from animal models, suggest potential mechanisms of action of KDs on depression.[9,10] First, KDs have been found to modulate neurotransmitter levels, including dopamine, serotonin, and glutamate,[11] which are important for mood regulation. By altering the signalling along these different networks, KDs may impact depressive symptoms. Additionally, KDs can increase the levels of endogenous noradrenaline,[12] creating an anticonvulsant effect similar to serotonin and norepinephrine reuptake inhibitors (SNRIs). Another mechanism is through the increased production of the inhibitory neurotransmitter GABA,[13,14] which helps regulate brain activity in individuals with depression. Second, KDs have shown potential in enhancing cellular bioenergetics and reducing oxidative stress. Impaired glucose metabolism and energy deficits are common features of depression,[15] and KDs offer an alternative fuel source through the oxidation of fatty acids, providing efficient energy production.[16] Animal studies suggest that KDs can lead to vascular brain changes, increasing capillary density at the blood-brain barrier and improving brain energy metabolism.[17] The HPA axis is a neuroendocrine system involved in maintaining homeostasis in humans under physiological conditions and stress, and cortisol is the main hormone of the HPA axis. The extent of HPA axis activity is thought to be influenced by an individual's nutritional environment, and studies have indicated that HPA axis tone may be inversely related to carbohydrate status, such that high glucose availability leads to decreased HPA activation.[18] Additionally, the waking cortisol response is increased, both in patients with current symptoms of depression and those who are not currently depressed, but at high risk of developing the illness. Cortisol levels therefore represent a plausible mechanistic pathway by which a KD may influence risk of depression. Third, KDs may reduce inflammation. Inflammatory markers (e.g. C-reactive protein) are abnormally

high in people with depression,[19,20] which are correlated with depression severity.[21] Recent data found decreased concentrations of pro-inflammatory cytokines in the blood in a ketogenic diet.[22] Fourth, KDs may change the gut microbiome and preliminary evidence suggests that gut microbes modulate brain function, particularly in relation to mood disorders. Studies in rodents have shown that a ketogenic diet reduced alpha-diversity (number of different species) of intestinal microbes but increased the abundance of Akkermancia muciniphila, a known SCFA producer, which is associated with improved metabolic health. These studies also demonstrated that the altered gut microbiome was associated with the seizure-reducing effects of the keto-diet, which was more prominent in stressed animals. Monitoring the changes in the gut microbiome following a ketogenic diet regime in people with treatment-resistant depression and correlating the abundance of microbial communities with subjective depression ratings may reveal the mechanisms underlying the potential psychotropic effect of a ketogenic diet.[23] Overall, clarifying the impact of KDs on the microbiome and cortisol and the association with changes in depression would help assess whether this is an important mechanism in the effect of KDs on depression if one is found.

Despite increased public interest and rapid market growth of KD food products (estimated $12.4 billion by 2024) [24] and evidence of biological plausibility demonstrated in epilepsy and animal models, the efficacy of KDs for treatment-resistant depression has not yet been established. To our knowledge, there are only three registered clinical trials underway testing KD for mental disorders, however, none examine efficacy of KD for treatment-resistant depression and there are no currently published results. A recent systematic review by Dietch et al. is the first to appraise the literature on KD for mental disorders in human samples and highlights the paucity of robust research. Twelve studies examining 388 participants (9 case reports, 2 cohort studies, and one observational study) met inclusion criteria and found no high-quality evidence of KD efficacy for mental disorders.[25] The absence of controlled trials, heavy reliance on individual case studies, grouping of various clinical disorders together, lack of uniform definition of KD across studies, including five studies not reporting KD nutritional intake, and no measurement of ketosis in half the studies make interpretation of the literature difficult. However, the review data suggest that people with various mental illnesses who follow a KD can have marked improvements in mental health. Moreover, a recent review suggests that symptom scales such as the PHQ-9 may be useful but should be better combined with measures of functioning and quality of life to fulfil patients' perspectives.[26]

The current study proposes to test whether a 6-week ketogenic diet is an effective treatment for treatment-resistant depression. We will aim to recruit around 100 participants with treatment-resistant depression. On completion of baseline assessments, participants will be randomised 1:1 into the KD group or control group. The KD group involves 6-week pre-prepared ketogenic diet (i.e. the ratio of fat to protein and carbs is 4:1) with weekly KD-focused behavioural and nutritional counselling. The control group will receive weekly nutritional counselling to increase vegetable consumption and reduce saturated fat intake. Participants in the control group will receive food vouchers (£20 every two weeks) to help purchase these items. This aims to be a plausible placebo dietary treatment for depression. Usual treatment for depression will continue, and a leaflet material is provided in both groups (**APPENDIX 9. Dietary materials for the intervention group** and **APPENDIX 10. Dietary materials for the control group**). The primary outcome will be assessed by the difference between groups in the change in self-reported depression severity, using the Patient Health Questionnaire-9 (PHQ-9) (**APPENDIX 8. SELF-REPORTED QUESTIONNAIRES**) at 6 weeks. The secondary outcomes are anxiety score, ability to experience pleasure, work and social adjustment, quality of life, and cognitive performance (**APPENDIX 8. SELF-REPORTED QUESTIONNAIRES**) from baseline to 6 weeks. This trial will also investigate potential mechanistic pathways (i.e. changes in cortisol and gut microbiome) that may explain the effect of a KD on depressive symptoms. We will follow participants to 12 weeks to assess whether they are following the assigned diet and the changes in primary and secondary outcomes.

In this study, a special concern is risk of self-harm and suicide, and further assistance and monitoring are in place. PHQ-9 will be completed every two weeks in the first six weeks and asks participants 'Over the last two weeks, how often have you been bothered by thoughts that you would be better off dead, or of hurting yourself in some way?'

with response options 'Not at all, several days, more than half the days, nearly every day.' Participants that report that they have had such thoughts more than half the days or nearly every day will have their risk of suicide assessed at the weekly telephone call using the procedure outlined in **Section I. Safety** and Error! Reference source not found.. Anyone judged to have a serious risk of suicide where an emergency response is initiated will be counted as having a serious adverse event.

Section F. Participants

1. Description of research participants

Adults aged 18-65 years with depression, who have been treated with at least two antidepressants in the current episode but still have moderate severity depression (≥15) as assessed by PHQ-9.

2. Inclusion Criteria

- Aged 18-65 years
- Have been diagnosed with depression
- Have had 2 or more antidepressant treatment trials of adequate dose and duration within the current depressive episode to which they did not fully respond. An adequate treatment trial is defined as at least 4 weeks of a medication at a minimum NICE-approved dose
- With a Patient Health Questionnaire-9 (PHQ-9) total score greater than or equal to 15 at baseline
- Able to understand and be willing to adhere to the demands of the study
- Provision of written informed consent
- Have access to a tablet/computer for online assessments, follow-ups with the registered dietitian, and able to attend appointments for assessments and treatment and adhere to study procedures
- Have both a fridge and a freezer at home
- Complete all baseline assessments

3. Exclusion criteria

The person may not enter the trial if ANY of the following apply:

- Currently following a low carbohydrate or ketogenic diet
- Currently following a vegan or vegetarian diet as these diets are more challenging to accommodate in a KD and adding vegetables in the control group is unlikely to be seen as helpful.
- Currently receiving, or have received, in-patient psychiatric treatment or electroconvulsive therapy (electric shock to the brain under brief general anaesthetic) within the past year, or scheduled to receive such treatment during the study
- Currently using St John's wort or other remedies for depression that were bought without a doctor's prescription
- Currently have suicidal ideation with intent* or attempted suicide within the past two months
- Ever had an eating disorder, bipolar disorder, schizophrenia, or psychosis
- Have substance use or alcohol dependence
- Have epilepsy

- Have serious food allergies (experiencing food hypersensitivity that leads to anaphylaxis or other severe symptoms, which may require hospitalisation or are life-threatening) or otherwise require a special diet that cannot be accommodated within a KD such as phenylketonuria or lactose intolerance
- Treated with insulin, sulfonylureas, GLP-1 analogues, or SGLT2 inhibitors
- Women who are pregnant, planning pregnancy in the next three months, or breastfeeding
- Have a body mass index (BMI) of <18.5 kg/m^2
- Have unstable or severe medical conditions (e.g., cancer, cardiovascular, renal, lung, psychiatric, or bleeding disorders, diabetes, etc.), currently receiving cancer treatment except hormonal treatment for breast cancer or non-melanoma skin cancer treatment
- Have gallstones, renal tubular acidosis, kidney stones, small bowel malabsorption or a history of pancreatitis
- Have scheduled a major surgery in the next 3 months
- Taking part in other studies that may compromise this study or this study may compromise the other study/ies
- Have read the trial protocol or the clinical trial registration information and therefore are unblinded
- Live in the same household as another participant in the trial
- Not able to complete the online task with a tablet/computer
- Not willing to provide saliva, urine and stool samples

* We define intent as someone who reports that they have thought of a means of killing themselves and are planning to acquire the means or have acquired them. We will ask participants whether they have ever attempted to end their life in the past in an open question, before narrowing down to assess whether this/these attempts were in the last two months. In the course of this conversation, the history of suicidality will emerge and the RA will keep this as a note, which will help us understand the level of risk.

4. Recruitment

The study will be advertised to the public via: 1) online social media platforms through advertisements created in collaboration with our industrial partner (Nativve Health Research: https://www.healthresearch.study/) (Error! Reference source not found.). The social media advertisements will link directly to a Nativve Health Research landing page (https://www.healthresearch.study/participate/DIME/), where eligible participants will be presented with an electronic participant information sheet (ePIS) (Error! Reference source not found.); 2) public advertisements placed in pharmacies, community centres, and other community venues. The public advertisements include a printed Nativve Health Research landing page with a QR code that can be linked to the electronic Nativve Health Research landing page.

5. Eligibility assessment

Following consent being received, eligibility will be assessed by a two-step consent process.
Step 1: Interested people will need to complete an anonymous online screening form to assess eligibility and provide explicit consent to do so but not full consent to enter the study to reduce participant burden. The screening questionnaire assesses the inclusion/exclusion criteria, and is shown in **APPENDIX 3. SCREENING**.

Step 2: If potential participants pass the screening questionnaire, participants will be invited to complete the study consent form. Following this, the researcher will conduct a telephone assessment to further evaluate: a) the nature of their usual diet (if applicable); b) the names, duration and doses of antidepressants that they have been taking; c) other medical conditions diagnosed by a doctor; d) the presence of symptoms of psychosis and suicide risk.

The screening for diet, antidepressants and psychosis will comprise asking the following questions and further questions to clarify meaning.

- Q1: “Are you following a specific diet?”
- Q2: if the answer to Q7 is “Yes”, then ask “which diet are you following?”
- Q3: “Can you tell me the names of the antidepressants you’ve been taking, how long you’ve been taking them, and the doses”
- Q4: “Have you had any strange or odd experiences lately that you cannot explain?”
- Q5: “Do you ever hear things that other people cannot hear, such as noises, or the voices of other people whispering or talking?”
- Q6: “Do you ever have visions or see things that other people cannot see?”
- Q7: “Do you ever feel that people are bothering you or trying to harm you?”
- Q8: “Has it ever seemed like people were talking about you or taking special notice of you?”
- Q9: “Are you afraid of anything or anyone?”

If a potential participant responds “No” to questions Q4-Q9, and a response to Q1/Q2 does not indicate a low-carb, ketogenic, vegan or vegetarian diet, they meet the eligibility criteria of not having psychotic symptoms and not currently following an excluded diet. If there is doubt, the RA will pass this to one of the clinical team in the study to assess further. If doubt remains, the person will not be enrolled. If the participant appears eligible, s/he will be invited to complete the baseline assessment and any eligible person who completes this will be enrolled and randomised.

The RA will also ask about suicide ‘risk’ based on the answer to PHQ-9 question 9 to assess whether potential participants can join the study. RA will ask the potential participant if they have been having thoughts that life is not worth living, or that they would be better off dead. If the person’s response indicates that they are experiencing these types of thoughts, the RA will then ask for further details regarding the person’s suicidal thoughts as necessary. This may include questions regarding the frequency and nature of the thoughts and whether specific suicide plans, actions, or attempts have been made. A flow chart has been constructed to demonstrate the appropriate course of action to take in these situations in relation to the person’s responses (Error! Reference source not found.).

The RA will ask participants about the nature of any other illnesses that they have and the treatments they may be receiving, particularly looking for markers that these are having an impact on the participant’s life or rapidly changing or especially severe. If in doubt, this will be passed to one of the medical assessors on the team.

6. Information Provided to Participants and Informed Consent

Participants will be asked to provide consent to anonymous screening. If participants appear to be eligible, they will consent to enter the study by completing Error! Reference source not found. and provide their full contact details. Participants must complete this form before the assessment phone call and any other study-specific procedures can commence.

The ePIS presented to participants will contain no less detail than: what it will involve for the participant; the implications and constraints of the protocol; and the known side effects and any risks involved in taking part. Further, it will be clearly stated that participants are free to withdraw from the study at any time, for any reason, and with no obligation to give the reason for their withdrawal. Potential participants will be allowed as much time as they wish to consider the information in the ePIS and eICF, and the opportunity to question the research team, or other independent parties to decide whether they will participate in the study. A copy of the signed eICF will be created on the study database platform, accessible only to the research team.

Section G. Research Procedures

1. Baseline Assessments and Procedures

If a person is eligible after full assessment, the researcher will send the potential participant a link by email or text to complete the baseline assessment online. The questionnaires and learning task will take about 1 hour. Participants will be asked to complete an online survey capturing socio-demographic information, medical history, questionnaires and tasks regarding mental health and associated quality of life and work (**Table**) (Error! Reference source not found. **BASELINE ASSESSMENT**), weigh themselves (we will provide a scale if participants do not have one), and provide saliva the first hour of the morning that is convenient to them, and a stool sample. We will collect data which are shown in **Table** , below:

Table 2: Self-reported data collection at baseline

1.	Contact details	Name and address, telephone numbers, email address.<br>Name and address and contact details of participant's GP
2.	Demographic and clinical information	Postcode, sex, age, marital status, level of education, ethnicity, number of episodes of depression, age of onset of first depression episode, current antidepressant treatment (type/dose), current psychological therapy for depression, and current medical conditions
3.	Self-rated questionnaires and assessments	Patient Health Questionnaire-9 (PHQ-9); Snaith-Hamilton Pleasure Scale (SHAPS); General Anxiety Disorder Scale (GAD-7); Perceived Deficits Questionnaire-5-item (PDQ-5); Work and Social Adjustment Scale (WSAS); Short-Form 12 Health Survey (SF-12); Probabilistic instrumental learning task (PILT)
4.	Self-reported anthropometric measures	Weight, height
5.	Saliva/stool samples	We will send participants a sample kit for collecting salivary cortisol and stool samples. Collection and packaging the stool sample will take less than 5 minutes plus time taken to post the sample in the pre-packaged and paid for posting pack. Salivary samples are taken in the first hour of the designated day and take about 2 minutes to collect and deal with each sample, which is collected by putting a dental roll in the mouth and extruding it into a plastic container. Participants will be able to do other things during collection but not eat. Samples will be collected on waking, and at 30, 45, and 60 minutes after waking. The samples will be sent back to the laboratory in a pre-addressed and stamped padded envelope.

Note: Postcode will be converted to an index of multiple deprivation score and the demographic variables will be presented by group at baseline for descriptive purposes only.

After all baseline assessments are complete, participants will be allocated by minimisation 1:1 to the KD group or control group. A researcher will embed a non-deterministic algorithm in the Redcap database. This algorithm aims to produce treatment groups balanced for important prognostic factors by minimising on the following variables (as below). Only after eligibility and consent is confirmed will the database reveal the allocation. Investigators delivering the intervention will not be blinded, but the outcome is collected blind (by self-report), and those involved in analysing the outcome data will be blinded to allocation.

- BMI (less than 30 kg/m2 vs. 30+ kg/m2)
- Depression severity (PHQ-9 score: 15-19 [moderately severe] vs. 20-27 [severe])

All follow-up is done online and without trial staff and will therefore not be subject to observation bias. Following randomisation, participants will be given information and advice for their respective group.

The questionnaire with details is described in **APPENDIX 6. BASELINE ASSESSMENT** and **APPENDIX 8. SELF-REPORTED QUESTIONNAIRES**. The probabilistic instrumental learning task consists of two pairs of symbols with one pair associated with win outcomes (win £1 or no change) and the other associated with loss outcomes (lose £1 or no change). Each symbol in the pair corresponds to reciprocal probabilities (0.7 or 0.3) of the associated outcomes occurring. Participants are required to choose between the two symbols in order to maximize their winnings. Once a choice is made, outcome feedback is provided. In order to maximize their winnings, participants use the outcome feedback to gradually learn the symbol–outcome associations over time, such that they consistently choose the symbol associated with high-probability win and avoid the symbol associated with high-probability loss. Note that different symbols are used at each visit to minimize learning effects confounding subsequent performance. Further details for this task are provided in this paper.[27]

2. Subsequent Visits

Follow-ups will be online only. Participants in both groups will be asked to complete questionnaires and tasks regarding mental health and quality of life and work (**Table 3**). Participants will be asked to weigh themselves, provide saliva (in the morning) and stool samples as outlined above. Two-week and 4-week assessments should take about 5 minutes to complete and the 6-week and 12-week assessment about 30 minutes. In the unlikely event that there are participants that default from completing the online questionnaires and have not contacted us, a researcher will make two attempts within 3-days to phone the participant directly to administer the PHQ-9 (primary outcome) over the phone.

We will invite about 20 participants to a Teams call one-to-one interview to discuss their experiences of trying to follow the diet assigned to them, the support on offer, and the perceived effects of it on their wellbeing. We will select participants to achieve a range based on their adherence to the diet and their response as measured by change in PHQ-9. We will analyse these interviews using simple content analysis. We anticipate interviews lasting up to 30 minutes. We will audio record them prior to analysis then destroy the recordings once the analysis is complete and we are confident of the meaning. The interviews will be audio only because both participants will turn off their video function. This will allow us to use the automatic transcription in Teams to enable us to use anonymised quotes in the write-up.

Table 3: Study schedule and follow-up assessments

Outcome measures	Baseline assessment (consent to the study)	2 weeks follow-up assessment	4 weeks follow-up assessment	6 weeks follow-up assessment	12 weeks follow-up assessment
PHQ-9	✓	✓	✓	✓	✓
GAD-7	✓	✓	✓	✓	✓
SHAPS	✓			✓	✓
PDQ-5	✓			✓	✓
SF-12	✓			✓	✓
WSAS	✓			✓	✓

PILT	✓			✓	✓
Substance use and health-related absence from work	✓			✓	✓
Weight	✓			✓	✓
Height	✓				
Saliva/Stool samples	✓			✓	
Interview					✓

Note: PHQ-9, Patient Health Questionnaire-9; GAD-7, General Anxiety Disorder Scale; SHAPS, Snaith-Hamilton Pleasure Scale; PDQ-5, Perceived deficits questionnaire-5-item; SF-12, SF Short-Form 12 Health Survey; WSAS, Work and Social Adjustment Scale; PILT, Probabilistic instrumental learning task.

3. Biological Sample Handling

All participants will be asked to give saliva and stool samples. Saliva samples will be returned to Medichecks (a company that has been previously approved by the University of Oxford as a third-party supplier) for analysis; Stool samples will be returned to the Oxford Centre for Microbiome Studies, where they will be stored until analysis. The centre has an HTA licence for storage (Ref. 12217) of human samples but the samples will be destroyed after batch analysis.

All saliva and stool kits come with postage-paid return envelopes provided by the laboratory responsible for testing. Return envelopes do not require participants to disclose their personal information. Participants' samples will be labelled with their study ID in place of their name and study name on the test kit so they are not identifiable. When the saliva and stool test kits are used, in order to send them to the laboratory and have them processed, no identifying participant information will be shared with the laboratory, as they will be identified with the study ID only.

Urine samples will be tested at home by the participants in the ketogenic diet as a self-monitoring aid. Participants will use a dipstick and the result recorded. The urine will be tipped down the toilet after testing.

4. Will the research include any audio, video or photographic recordings?

Yes. The interviews for participants will be conducted after the 12-week follow-up via secure, encrypted software licensed by the university (Microsoft Teams), and the interviews will be audio-recorded at the consent of the participant to help with summarising the findings (**Appendix 16**). The cameras of the interviewer and the participant will be turned off. Data will be securely kept in a password-protected University shared drive with access restricted to the research team. Some qualitative interviews with participants in the ketogenic diet arm will be transcribed verbatim by Microsoft Teams recording facility to help create quotes. The recordings will be deleted once interviews have been transcribed and the transcripts have been checked for accuracy and clarity, and data analysis completed.

5. Discontinuation/Withdrawal of Participants

If the participant meets any one of the following conditions, the Investigator will stop the treatment at his/her discretion. The participant, however, will not be considered to have dropped out of the study. This may happen for several reasons, including but not limited to:

a. The participant wishes to stop the treatment.

b. The Investigator judges that it is difficult to continue the treatment because of emergence of serious adverse events (SAE) as defined below.
c. The Investigator judges that the risk outweighs benefit in continuing the treatment.
d. The participant in the KD arm becomes pregnant.
e. The Investigator judges that it is inappropriate to continue the treatment for other reasons, such as bereavement.

If a participant wishes to withdraw from follow-up, data collected up to the point of withdrawal will still be used, as detailed in the ePIS. The reason for follow-up withdrawal, if provided, will be recorded on the eCRF. No participants will be replaced if they discontinue treatment or withdraw from follow-up.

6. Definition of End of Study

The end of study is set as the date of resolution of the last data query.

7. Please detail all expenses or gifts that will be offered to participants.

All participants will be reimbursed for their time and effort after each fully completed assessment after baseline (vouchers = £10 at week 2 and week 4; £30 at week 6; £30 at week 12; £10 qualitative interview after week 12). A pro-rated £10 gift voucher will be offered to the participant for partial completion where PHQ-9 (primary outcome) is the only measurement administered over the phone. Participants randomised to the control group will receive £20 shopping vouchers every two weeks to purchase vegetables and fat replacement. Participants in the ketogenic diet group will receive food to support dietary adherence.

Section H. Interventions

Drug/Substance 1

**Name of drug/substance to be used**	A 4:1 (fat: protein+carbohydrates) pre-prepared KD meals and snacks with weekly KD-focused nutritional counselling
**Formulation, dose and route of administration for research**	4:1 (fat: protein + carbohydrates) pre-prepared KD meals (3 meals per day) and snacks without energy restriction. Each nutritional counselling session typically lasts 20-30 minutes.<br><br>In this study, a registered dietitian will contact participants to enquire about their current diet and provide advice on how to change it. Participants will be offered and encouraged to use pre-packaged meals from a commercial ketogenic diet supplier to enhance adherence and reduce the burden on participants. However, participants will be free to mix and match appropriate non-packaged food or additional ketogenic foods in their diet in consultation with the dietitian. The dietitian will provide weekly support for up to 30 minutes and enquire about issues and experiences, troubleshooting as necessary, and provide guidance on how to prevent or overcome side effects of KD diets, and keep record of the participant's ketosis level. As the diet progresses, if the participant wishes to continue following the diet, the dietitian

	and participant will work on how to transition away from pre-packaged foods.<br>The foods require storing in a fridge and will be sent a week at a time so will require some space to store them.<br>Previous studies of the KD intervention have reported mostly mild and transient side effects, commonly referred to as "keto flu". These side effects may appear two to seven days after starting a ketogenic diet, including headache, foggy brain, fatigue, irritability, nausea, difficulty sleeping, and constipation. These symptoms usually disappear by themselves. The team will also provide a pamphlet explaining the benefits and risks associated with the study and KD meals, as well as offer solutions to any problems that may arise (**APPENDIX 9. Dietary materials for the intervention group**).<br>We also provide home urine test strips for ketosis and ask participants to measure ketosis at least twice a week. The main purpose is to reassure the participant and the dietitian that they are in ketosis. The dietitian will record these results, as it is evidence that the participant has achieved ketosis.
**Duration of treatment for research**	6 weeks
**Licence status of this drug/substance**	N/A
**Usual Indication**	Used for treatment of diabetes, weight loss, treatment of epilepsy or other reasons usually at people's own instigation.
**Usual Dose**	3 meals with snacks per day without energy restriction
**Usual duration of treatment**	6 weeks
**Where will drug/substance be sourced from?**	Meal providers for NHS services
**Where will drug/substance be stored at site?**	It will be prepared by a company in the UK
**How will drug/substance be dispensed?**	Meals and snacks will be directly shipped to the participant's address and stored by the participant, mostly out of a fridge.
**How will the drug/substance be prepared by the researchers for use in this research?**	N/A

Section I. Safety

**1. Definitions**	
**Adverse Event (AE)**	Any untoward medical occurrence in a participant to whom a substance has been administered, including occurrences which are not necessarily caused by or related to that substance.

**Adverse Reaction (AR)**	An untoward and unintended response in a participant to a substance, which is related to any dose administered to that participant.<br>A causal relationship between the administered substance and an AE is at least a reasonable possibility, i.e. the relationship cannot be ruled out.
**Serious Adverse Event (SAE)**	A serious adverse event (SAE) is any untoward medical occurrence that:<br>• results in death<br>• is life-threatening<br>• requires inpatient hospitalisation or prolongation of existing hospitalisation<br>• results in persistent or significant disability/incapacity<br>• consists of a congenital anomaly or birth defect.<br>Other adverse events that require medical intervention may also be considered a serious adverse event when, based upon appropriate medical judgement, the event may jeopardise the participant and may require medical or surgical intervention to prevent one of the outcomes listed above. This would include threatened suicide that was prevented by an emergency response. Any planned admissions that were scheduled at the start of the study will not be counted as SAEs.<br>NOTE: The term "life-threatening" in the definition of "serious" refers to an event in which the participant was at risk of death at the time of the event; it does not refer to an event which hypothetically might have caused death if it were more severe.<br>All participants will be prompted to self-report SAEs at the weekly dietitian call. At 12 weeks, we will ask participants about hospitalisation in the last 6 weeks and phone participants who describe an episode to elicit the details.
**Serious Adverse Reaction (SAR)**	An adverse event that is both serious and, in the opinion of the reporting Investigator, believed with reasonable probability to be due to one of the research treatments, based on the information provided.
**Suspected Unexpected Serious Adverse Reaction (SUSAR)**	All SARs would be deemed SUSARs given neither treatment is expected to cause an SAE.

2. Reporting procedures for serious adverse events or reactions

Only a serious adverse event (SAE) occurring to a participant will be collected in this study. Reports of related (resulted from administration of any of the research procedures) and unexpected (the type of event is not listed in the protocol as an expected occurrence) SAEs will be reported to the ethics committee and the study steering committee (SSC). For fatal and life-threatening suspected unexpected serious adverse reactions (SUSARs), this will be reported as soon as possible, but no later than 7 calendar days after the PI is first aware of the reaction. Any additional relevant information will be reported within 8 calendar days of the initial report. Non-fatal or non-life-threatening SUSARs will also be reported as soon as possible, and no later than 15 days after the PI is first aware of the reaction.

AEs that are not serious will not be collected, as they offer no scientific value in our case, and have been extensively collected and reported in previous studies of ketogenic diets and are well known. Collecting only SAEs will reduce

the burden to participants such as life-threatening events, incidents resulting in persistent or significant disability/incapacity.

3. Safety of participants

1. What level of baseline safety screening will take place for this research?

If potential participants pass the questionnaires for screening, the researcher will conduct a telephone assessment to assess whether they are on a specific diet before participants can be enrolled, the names and doses of antidepressants, other medical conditions diagnosed by a doctor, and the presence of symptoms of psychosis and suicide risk. Trained RAs will make notes on the semi-structured interview document, recording the main features of the phone call, highlighting areas of particular concern and any other specific details as appropriate. This ensures reliable information is shared and supports accurate safety reporting. The semi-structured interview documentation will be forwarded to the local clinician for their review if there are any reports of suicidal thoughts.

2. Provide details about the safety monitoring of participants and the staff/researchers carrying this out

Safety monitoring

The team includes two consultant psychiatrists and one of the co-PIs who has received training in suicide assessment and response from the US Department of Veteran Affairs. They will provide training for our RAs with a safety manual that outlines the expectations for the assessments, threshold criteria for contacting 999 or GPs or clinical staff for risk assessment, and common issues that may arise during screening assessment (**APPENDIX 14. SUICIDE ASSESSMENT TRAINING MANUAL**). A link to UK mental health crisis resources will be made available on the study landing page. We will send an automated email to all participants with a link to the resource list at baseline for accessing help should they feel like ending their lives (**APPENDIX 15. Participant Email SMS Scripts**).

The co-PI will facilitate a half-day training for the RAs to review the safety manual, review the assessment measure, and engage in role-plays and supervised practice assessments on how to administer a validated screening interview assessing suicidality. If the role plays are satisfactory, the co-PI will shadow the RA for at least 5 participant screening conversations with potentially eligible participants to assure this process and communication that follows it is done correctly. If the role plays do not meet the required standard, the co-PI will provide additional training for the RAs, review their conversations with participants, and shadow the RA for an additional 3 participant screening conversations until the process and communication is executed correctly.

Suicide or self-harm risk

PHQ-9 will be completed every 2 weeks and asks participants 'Over the last two weeks, how often have you been bothered by thoughts that you would be better off dead, or of hurting yourself in some way?' with response options 'Not at all, several days, more than half the days, nearly every day.' Participants that report that they have had such thoughts more than half the days or nearly every day will have their risk of suicide assessed by a telephone call using the procedure outlined in Error! Reference source not found.. If the participant screens positive for "high risk of suicide" a triage guideline will be followed if a positive "high risk" of suicidal intent/plan has been indicated. Participants will be notified that they are not currently eligible for the study at this time, and that further evaluation will be required. We will ring 999 if the participant is in crisis. If there is a significant but not imminent danger, we will inform the GP as outlined in our flow diagram. Regular debriefing and reliability meetings with RAs, psychiatrist consultants, and the lead co-PI will take place to discuss this process. If a participant discloses risk of suicide intent or attempt after baseline and is assessed as not imminently at high risk, the participant will be reminded about the crisis resources by the assessor and resent the link of crisis resources (**APPENDIX 7**).

3. Give details on the medical cover required and who will provide this cover
We have registered medical doctors in the investigating team who are used to assessing patients with psychiatric disorders. Should suicidal ideation be uncovered or other untoward occurrences, the clinicians will respond appropriately, mostly to ensure that usual medical care is provided by the participant's own GP or mental health crisis team. The trial team will ensure that clinical cover is available each day that data is recorded.<br><br>A researcher will contact participants the next working day if the response to the PHQ-9 death and suicide question means this is necessary.

4. Will the participants' GP be informed about their participation in the research? In not, please justify
Participants' GPs will receive one letter at baseline after randomisation and another providing a summary of the participant's progress in depression and anxiety severity at 12 weeks. If another letter is needed to make the participant's GP aware of an issue, this will be sent ad-hoc as soon as possible.<br>We will also inform the participant's GP if potentially serious events occur and the participant is not competent to decide on whether to seek medical assessment.

5. What is your planned procedure if an incidental finding is suspected?
In the unlikely event that a person discloses that they have just attempted, or are imminently about to attempt suicide, RAs should attempt to identify the person's current location, if they have not already, and call 999 to alert the emergency services.<br>If the person mentions they have made specific suicide plans, RAs should ask what the person plans to do, if they know where or when they would do it, whether they have access to what they need to carry out the plan, and if they intend to carry it out. If the person states that they plan to carry this out right now, RAs should identify the person's current location and call 999 to alert the emergency services.<br>If the person responds that they think of suicide or death very frequently or in some detail, but do not mention any specific plans for suicide, RAs should ask the person to tell them more about what has been going through their mind and follow the relevant line of questioning dependant on the answers given (see Error! Reference source not found.).<br>Unless the call has ended prematurely due to the person disclosing that they will be attempting suicide, RAs should inform the person that they will be sharing the content of this phone call with a clinical member of the research team.<br>The RA will contact a clinical member of the research team and relay the details of the conversation with the person, to discuss thoughts disclosed around suicide, and provide details of any actions taken thus far. Emergency contact should be made by telephone and followed up with an email confirming the details of the conversation. However, if the person is known to have ongoing suicidal ideation and the GP or mental health services have a plan as to how to respond to it (e.g. if local services is already supporting the patient for ongoing suicidality and the current report is a continuation of this), then any subsequent communication with the clinician may be by email rather than phone. This is outlined in **APPENDIX 7. FLOW CHART FOR ASSESSING RISK OF SUICIDE** at baseline and after each PHQ-9 questionnaire.

6. If an incidental finding has clinical implications, what action will you take?
The assessments concern psychological symptoms, health, and functioning, cortisol, and stool microbiome. There is a risk of the participant declaring suicidal intent and we will follow **Appendix 7. FLOW CHART FOR ASSESSING RISK OF SUICIDE** to deal with this. In the very unlikely event that cortisol concentrations are very low or unusually high, we will inform the participant's GP. No other incidental clinical findings can be revealed by these assessments.

4. Ethical considerations		
Research usually carries the risk of some ethical challenge. If this is the case you need to demonstrate your awareness of the problem and your response to mitigate ethical objections.<br>For guidance on ethical issues, please see http://researchsupport.admin.ox.ac.uk/governance/ethics/resources, however the following areas are often a cause for concern:		
**1. Will the research involve any participants considered vulnerable in the context of the research (e.g. children, elderly, prisoners, adults at risk)?**<br>**If yes,** please describe how they are defined as vulnerable and detail any CUREC Approved Procedures or guidance that will be applied to the research (for current documents and templates see https://researchsupport.admin.ox.ac.uk/governance/ethics/resources).	Yes ☐	No ☒
During the screening stage we will not enrol participants at risk, such as participants with high suicide risk, psychotic disorders, and other serious physical illnesses.		
**2. Will the research involve deliberate deception of participants?**<br>**If yes**, justify why deception is used, describe deception and debriefing process, and include debriefing documents in the application	Yes ☐	No ☒
The aim of the study will be described accurately, but in broad terms, rather than specific detail. The PIS will describe the aim to assess the impact of a dietary intervention, without specifically mentioning ketogenic diet, as this could influence participants' dietary behaviours such that participants in the control arm choose to follow this diet, which will contaminate the study and could make the results difficult to interpret and undermine its purpose. Once randomisation has occurred, the participant will receive educational materials and consultation with a registered dietitian about the specific dietary intervention. At the end of the study, we will write to all participants to share the hypothesis and provide the results (**APPENDIX 12. Final letter for participants**).		
**3. Could the proposed research affect your own physical and/or psychological safety as a researcher?**<br>**If yes,** describe how this will be mitigated.	Yes ☐	No ☒
**4. Does your research raise issues relevant to the Counter-Terrorism and Security Act (the Prevent Duty), which seeks to prevent people from being drawn into terrorism?**<br>**If yes,** please say how you plan to address any related risks. Please see advice on this on our Best Practice Guidance Web Page.	Yes ☐	No ☒
**5. Please list any stakeholder or community engagement that has been, or will be, undertaken in relation to the research**		
For the development of this protocol, we engaged in public involvement through virtual 1:1 or 1:2 sessions with 8 members of the public who have depression, of whom 3 had previously followed a KD diet.<br><br>Here are the comments from PPI members and how we have adapted them into our protocol, as outlined below.<br><br>**Recruitment**		

1. Members of the public indicated that social media might not effectively reach older populations, suggesting alternative recruitment strategies such as community centres, newsletters, local trusts, hair salons/barbers, mosques, and faith groups.
   In response to this feedback, we have adapted our protocol to include social media advertising and public advertising to engage participants across all age ranges.
2. Members of the public expressed openness to providing biological samples while emphasising the need for clear explanation regarding the significance of such samples.
   In response to this feedback, our participant-facing materials explicitly emphasise the importance of collecting samples to gain insights into the value of cortisol and gut microbiome in explaining study outcomes.

Intervention

3. Most public members wanted to be able to have snacks throughout the day.
   In response to this feedback, we will provide standard meals supplemented with a variety of snacks.
4. Public members expressed varying opinions regarding pre-prepared versus fresh food. It was noted that fresh food accompanied by recipe cards could enhance confidence and self-efficacy, with potential benefits extending beyond the programme. Some people favoured ready meals while others preferred cooking from pre-portioned ingredients.
   In response to this feedback, and considering the aim of this study, which is to investigate the impact of a ketogenic diet on depression severity, we have decided to provide pre-prepared meals. This adaptation aims to alleviate any concerns participants may have about feeling overwhelmed by cooking tasks and ensures the study's scientific rigour is maintained.
5. Public members expressed that they would find motivational messages helpful.
   In response to this feedback, we will integrate motivational messages within our two dietary groups during the dietary counselling visits. These messages aim to inspire and encourage participants throughout their engagement with the study, building a positive mindset and enhancing their adherence to the ketogenic diet.
6. Public members highlighted the importance of engaging family members to provide support, recognising their influential role in the dietary choices of participants.
   In response to this feedback, we will develop informative leaflet materials specifically designed for family members. These materials will emphasise the significance of their support in both the well-being of the participants and the success of the study.
7. Public members expressed concerns regarding their uncertainty about what to do in the event of unintentional lapses from the KD diet for a few days.
   In response to this feedback, we will make sure that the dietary visits use behavioural strategies aimed at addressing relapsing and relapse prevention. This is an approach used by dietitians as part of standard clinical practice.

6. Please give details of any other research-specific ethical and/or safety considerations, not related to drug/substance administration

N/A

7. Will any data or information from this study be provided to individual participants?

Yes. A lay summary of the results of the study will be e-mailed to all participants when the final papers are published. The data on cortisol and microbiome have no easy interpretation nor implications for health for individuals so we do not plan to send those to participants.

Section J. Statistics and analysis

1. **Do you have a statistical plan?**<br>**If no,** please justify.	Yes ☒	No ☐
A detailed statistical analysis plan will be finalised and published ahead of the recruitment start date. We have described the outline plan here.		

2. **Number of Participants**
100 participants

3. **Have you done a sample size calculation?**<br>If yes, please give details below<br>If no, please give details to indicate you have considered the implications the selected sample size will have on the research outcome	Yes ☒	No ☐
We consider a 5-point difference to represent a clinically important difference, as suggested by Lowe et al.[28] This study reported that the SD of the change in PHQ-9 over 3 months as 5.8 and as 6.1 over 6 months. Assuming an SD of 6 over 6 weeks would suggest a standardised effect size of 0.83 in PHQ-9 to be clinically relevant. The sample size to test differences between groups at 90% power and at a type one error rate of 5% would be 64 (32 per group). A sample size of 100 participants will be recruited to make the study robust against up to 35% attrition/missing data.[27, 28] In simulations we confirmed that this sample size remains robust under scenarios such as unequal sizes of the strata used in minimisation.		

4. Analysis of Outcome Measures

Initial descriptive analysis will present the profile of the subjects by study arm without using statistical comparisons. For the primary hypothesis, the change in PHQ-9 scores from baseline to week 6 will be compared between groups. A mixed effect model that includes treatment group, time and their interaction as fixed effects, and individual subjects as random effects will be fit using PHQ-9 at all available assessments. A linear contrast will be used to test the difference between groups in changes from baseline to week 6 in PHQ-9 scores. Two tailed tests and significance level of 0.05 will be used. Secondary outcomes will be analysed using analogous mixed effects generalised linear models. Pre-specified subgroup analyses of the primary outcome will be explored by baseline depression severity (severe versus moderate) and duration of depression at baseline split at the median. Differences in AEs by study arm will be compared and presented as proportions as described in **Section I. Safety** and differences will be assessed using Fisher's exact test.

We will conduct an exploratory mediation analysis using methods described by Valeri & Vanderweele [29] to examine whether adherence to the diet, changes in microbiome, and cortisol awakening response appear to mediate changes in depression assessed by PHQ-9. Extracted faecal information will be sequenced to obtain a global representation of the microbiome compositions, and Alpha diversity will be measured using Observed Species, Whole Tree Phylogeny, and Shannon and Simpson indices. Spearman correlations will determine associations between alpha diversity metrics, individual microbes, and PHQ-9 scores. Kruskal–Wallis one-way analysis of variance tests will be performed to compare relative abundance of the top ten genera, with false discovery rate (FDR) corrections between dietary groups.

The extent of missing data will be reported descriptively, including the number of missing data in each group, and baseline characteristics of people with and without missing outcome data. The primary analysis, with mixed models, is valid under the assumption that data is missing at random (MAR). The robustness of the conclusions to the MAR assumption will be tested in sensitivity analyses as below –

1. Sensitivity analysis assuming everyone with missing outcome data has severe depression
2. Sensitivity analysis assuming everyone with missing outcome data has no depression (included for completeness, not for plausibility)

3. Repeat main analysis with analysis restricted to (a) those with maximum of one missing observations (b) those with maximum of two missing observations

Complete case analysis will be used as the primary analysis if the proportion of missing data is below 5%.[30] We will conduct a sensitivity analysis to explore reasons for missingness and determine appropriate handling of missing data. The following approaches may be applied based on the missing data: single imputation (e.g., last observation carried forward) and full information maximum likelihood estimation. Intention-to-treat analysis using baseline or last observation carried forward (LOCF) are recommended for parallel group randomised trials by CONSORT guidelines since they provide a full report of any deviation of the intervention and includes every participant who enrolled in the study and completed baseline measures, regardless of whether they completed the intervention.[31] ITT also gives an unbiased estimate of the treatment effect. If data are missing not at random (MNAR) for our continuous dependent outcome variables, full information maximum likelihood estimation will be used because it is more compatible between the imputation and analysis models compared to multiple imputation.[30]

Section K. Data management and handling

All information provided by participants is considered **research data** for the purpose of this form. Any research data from which participants can be identified is known as **personal data**; any personal data which is sensitive is considered **special category data**.

Management of personal data, either directly or via a third party, must comply with the requirements of the UK General Data Protection Regulation (GDPR) and the Data Protection Act 2018, as set out in the University's Guidance on Data Protection and Research. In answering the questions below, please also consider the points raised in the Data Protection Checklist and whether, for higher-risk data processing, a separate Data Protection Impact Assessment may also be required for the research. Advice on research data management and security is available from Research Data Oxford and your local IT department. Advice on data protection is available from the Information Compliance team.

1. Please mark 'X' against the data you will collect for your research

Screening documents	☒	Audio recordings	☒
Consent records including participant name or other identifiers (e.g. written consent forms, audio-recorded consent, assent forms)	☒	Video recordings	☐
Consent obtained anonymously (e.g. via online survey)	☒	Transcript of audio/video recordings	☒
Opt-out forms	☐	Photographs	☐
Contact details for the purpose of this research only	☒	Information about the health of the participant (including mental health)	☒
Contact details for future use (guidance)	☒	Physiological test results / measurements	☐
Task results (e.g. questionnaires, diary completion)	☒	Scans (e.g. MRI, Ultrasound)	☐

Data already in the public domain.<br>Specify the source of the data:	☐	IP addresses (refer to Best Practice Guidance 09: Data collection, protection and management for guidance)	☐
Previously collected (secondary) data	☐	Other (please specify below)	☒
Bank (or other) details required for reimbursement	☐	**Data generated from Biological samples**	

2. How and where will each type of data be stored whilst the research is ongoing (until the end of all participant involvement)?

List each type of data selected above, and explain how each will be physically transferred (including movement/sharing of audio files, paper records, electronic downloads etc.) from where it is collected to a suitable storage site (e.g. Nexus365 OneDrive for Business, SharePoint, University servers). State the storage location for each.

Refer to Best Practice Guidance on data collection, protection and management (BPG09).

Screening documents: Participants will be anonymously consenting to the eligibility process in the beginning. The screening will take place online on a secure encrypted online platform (the study eCRF). Screen failures will not be allowed to go through the eligibility assessment again, as the system will recognise the IP address and block any other attempt. This safeguards people who cannot be enrolled in the study for safety reasons due to exclusion criteria they might meet. This anonymous data will be analysed as part of the CONSORT flow chart and kept for 3 years, as the other data.

Consent records: Only if potential participants pass the initial screening process will they be asked to complete the full consent form for the study and then give us their name and contact details. This can generate a PDF consent form to be downloaded by the participant. The consent forms will be securely stored in a password-protected study folder on the university's secure and encrypted drive. After being downloaded from the encrypted online platform, they will be retained for a period of 3 years following the completion of the study. Any video recordings will be deleted at the end of the study.

Contact details for the purpose of this research only: The research team will use participant contact details to contact individuals about the research study and make sure that relevant information about the study is recorded for participant care, and to oversee the quality of the study. We will also collect and store GP contact details to send one letter at baseline after randomisation and another providing a summary of the participant's progress in depression and anxiety severity at 12 weeks. Such information will be kept online on the study database server and in the end of the study, in a separate database to the one containing research data, on the secure university drive.

Contact details for future use: If participants select this option in the consent form, we will keep their name and contact details (i.e. e-mail, telephone) in a secure separate database for 7 years, to inform them about relevant future studies.

Questionnaire and task results: All questionnaire data will be entered onto the online database. For the PILT task, data will be collected online using a task run on the servers of Gorilla online (owned by Cauldron Science ltd). Participants will link anonymously to the URL of the Gorilla based task. All data stored on the Gorilla server will therefore be anonymous. Gorilla servers are encrypted and are within the EU. At the end of the study, we will download and store the anonymised research data securely at the University of Oxford indefinitely. Data will be transferred to the statistical software for analysis and we will perform validation checks (e.g. range checks).

Test results: All test results will be identified by the study ID only and be pseudonymous. They will be provided in spreadsheet format to the study team and deleted from the host laboratory. They will be linked into the analysis database and stored for up to 7 years.

The participants will be identified by a unique study specific number and/or code in this database. The name and any other identifying detail will NOT be included in any database used for data analysis.

Audio recordings: The qualitative interviews are planned to be conducted using Microsoft Teams with the video of both participants and interviewer turned off. This is a licensed secure and encrypted software approved by the University of Oxford. This platform also provides the capability to generate transcriptions, forming the foundation for creating a permanent and anonymized record of the interviews. The recording process will commence once verbal consent is obtained from the participants through Microsoft Teams. Subsequently, access to the .mp3 files will be tightly controlled and limited exclusively to the designated research team members tasked with analysing these. These individuals will be the only ones authorised to access the recordings for the purpose of this study.

Other Information about the health of the participant: We will collect data on participants' depression and treatment of it, key elements of medical history and demographic data to describe participants in the study. All

study data will be entered on to the eCRFs. At the end of the study, we will download, delete from the study database platform and Gorilla server, and store the anonymised research data securely on the University of Oxford network drives. Data will be transferred to the statistical software for analysis and we will perform validation checks (e.g. range checks).
**3. Will you use a unique participant number on research data instead of participant name?**<br>**If yes,** state whether or not you will retain a list of participant names against numbers (pseudonymisation via a linkage list).<br>**Where will the list be stored, and when will it be destroyed?**
Yes, we will retain a list of participant names against their unique code, using the method of pseudonymisation. This list will be kept in a separate file from all other documents in a secure drive with limited access on a strictly need-to-know basis, using password protection. This linkage will be retained until the end of the study then destroyed.
**4. Who will have access to the research data?**
Researchers listed on this form, the SSC, and other trained researchers if deemed necessary (e.g. statisticians) will have access to the research data. Access will be granted to the MS IDREC for the purposes of monitoring and/or audit of the research.
**5. If research data is to be shared with another organisation, how will it be transferred / disclosed securely?**
We have no plans to share the data, but we are conscious that sometimes researchers ask for access to the data. We will share anonymised data with bona fide researchers with a clear purpose under a data sharing agreement.
**6. When and how will identifiable data be destroyed or deleted?**<br>N.B. If any identifiable data will be retained beyond the end of the study and/or indefinitely, please state what data this is, and the reasons for retention (e.g. contact details for future studies; photos used in publication). This must be clearly stated on participant information, and specific consent obtained.
Recordings of the interviews will be deleted after analysis but the transcripts kept for 3 years after publication.<br>We will keep consent forms for up to 7 years to ensure that there is a comprehensive and consistent record of participants' agreement to participate in the study. These will then be deleted permanently from the drive.<br>We will keep participants' contact details and their GPs' contact details until the study results are published in order to send them a lay summary, and the separate contact information database will be then deleted permanently from the drive. There will be a separate database with contact details for participants who wish to be contacted for future studies. This will be kept for up to 7 years and then permanently destroyed.

**7. Please confirm that you will store other (non-identifiable) research data safely for at least 3 years after final publication or public release and adhere to any additional research funder policies.**<br>For more information about the University policies, please see the University's web pages on research data management.<br>**If 'Yes'**, please give details of who will store the data and on storage format, location and security.<br>**If 'No'**, please provide further details.	Yes ☒	No ☐
The final database and the qualitative interview transcripts will have identifying information removed and only these versions will be kept indefinitely in a safe drive with strict-access by the study team on a need-to-know basis. In case a research team requests this database in the future for research purposes (e.g. an individual participant data meta-analysis), each request will be reviewed by the PI and only after establishing a data-sharing agreement between the universities/teams.		

Section L. Monitoring and oversight

1. Who will be responsible for day-to-day supervision of the research?

Dr. Min Gao and Dr. Megan Kirk Chang will be responsible for day-to-day running of the research. Professor Paul Aveyard will supervise them and the study throughout this research.

Regular monitoring will be performed by the study team. Data will be evaluated for compliance with the protocol and accuracy in relation to source documents. The monitors will verify that the clinical study is conducted and data are generated, documented, and reported in compliance with the protocol, GCP and the applicable regulatory requirements.

2. Give information about frequency of meetings that will be held to discuss progress/problems. Who will be present at the meetings?

Study management group

The study management group will comprise of all named investigators and it will be responsible for the day-to-day running of the study and meet monthly to evaluate progress. Study coordinators and Professor Paul Aveyard will meet weekly every two weeks to evaluate progress. The frequency of the meetings may be adjusted depending on the progress.

Study steering committee

The committee will consist of clinicians with relevant clinical expertise and experience in clinical trials. They will not be members of the University of Oxford and not related to the investigators. According to NIHR's Research Governance guidelines[32], the SSC will examine the protocol and give advice on the conduct of the study and take on the roles of the DMEC with respect of monitoring safety if this is required. This is an open-label trial where the interventions are dietary and not known to cause harm and there are no early stopping rules that would require this. The SSC will receive and review information on the progress and accruing data of the trial and will provide advice on the conduct of the study to the investigators.

Section M. Ethical and regulatory considerations

Declaration of Helsinki

The Investigator will ensure that this research is conducted in accordance with the principles of the Declaration of Helsinki.

Approvals

The application form/protocol, informed consent form, participant information sheet and any proposed advertising material will be submitted to the Medical Sciences IDREC for written approval.

The Investigator will submit and, where necessary, obtain approval from the above parties for all amendments to the original approved documents.

Annual Progress Report

The CI shall submit an Annual Progress Report to the Medical Sciences IDREC within one month of the anniversary of approval.

Section N. Insurance

The University has a specialist insurance policy in place which would operate in the event of any participant suffering harm as a result of their involvement in the research (Newline Underwriting Management Ltd, at Lloyd's of London).

Section O. Dissemination and feedback of research outcomes

1. Will you preregister this research?	Yes ☒	No ☐
2. If yes, please state the platform where it will be preregistered	We will register the study at https://clinicaltrials.gov/, once ethical approval is confirmed.	
3. How will you disseminate project outcomes at the end of the research?	The protocol and results will be published in open access journals and will be presented in conferences nationally and internationally. We will also compile a results summary for participants and lay members, to be disseminated to the study participants and other relevant partner organisations, stakeholder groups, and charities.	

Section P. References

1. Ali S, Rhodes L, Moreea O, et al. How durable is the effect of low intensity CBT for depression and anxiety? Remission and relapse in a longitudinal cohort study. 2017; **94**: 1-8.
2. Conway CR, Gebara MA, Walker MC, et al. Clinical characteristics and management of treatment-resistant depression. *The Journal of clinical psychiatry* 2015; **76**(11): 2709.
3. Rush AJ, Trivedi MH, Wisniewski SR, et al. Acute and longer-term outcomes in depressed outpatients requiring one or several treatment steps: a STAR* D report. 2006; **163**(11): 1905-17.
4. Page CE, Coutellier L. Prefrontal excitatory/inhibitory balance in stress and emotional disorders: Evidence for over-inhibition. *Neuroscience & Biobehavioral Reviews* 2019; **105**: 39-51.
5. Duman RS, Sanacora G, Krystal JH. Altered Connectivity in Depression: GABA and Glutamate Neurotransmitter Deficits and Reversal by Novel Treatments. *Neuron* 2019; **102**(1): 75-90.
6. Martin K, Jackson CF, Levy RG, Cooper PN. Ketogenic diet and other dietary treatments for epilepsy. *Cochrane Database Syst Rev* 2016; **2**: Cd001903.
7. Augustin K, Khabbush A, Williams S, et al. Mechanisms of action for the medium-chain triglyceride ketogenic diet in neurological and metabolic disorders. *The Lancet Neurology* 2018; **17**(1): 84-93.
8. Ułamek-Kozioł M, Czuczwar SJ, Januszewski S, Pluta R. Ketogenic Diet and Epilepsy. *Nutrients* 2019; **11**(10).
9. Neves GS, Lunardi MS, Lin K, Rieger DK, Ribeiro LC, Moreira JD. Ketogenic diet, seizure control, and cardiometabolic risk in adult patients with pharmacoresistant epilepsy: a review. *Nutr Rev* 2021; **79**(8): 931-44.
10. Brietzke E, Mansur RB, Subramaniapillai M, et al. Ketogenic diet as a metabolic therapy for mood disorders: Evidence and developments. *Neuroscience and biobehavioral reviews* 2018; **94**: 11-6.
11. Neal EG, Cross JJohn, dietetics. Efficacy of dietary treatments for epilepsy. 2010; **23**(2): 113-9.
12. Weinshenker D. The contribution of norepinephrine and orexigenic neuropeptides to the anticonvulsant effect of the ketogenic diet. *Epilepsia* 2008; **49**(s8): 104-7.
13. Yudkoff M, Daikhin Y, Nissim I, et al. Response of brain amino acid metabolism to ketosis. 2005; **47**(1-2): 119-28.

14. Düking T, Spieth L, Berghoff SA, et al. Ketogenic diet uncovers differential metabolic plasticity of brain cells. *Science advances* 2022; **8**(37): 2375-548.
15. Fattal O, Budur K, Vaughan AJ, Franco KJP. Review of the literature on major mental disorders in adult patients with mitochondrial diseases. 2006; **47**(1): 1-7.
16. Yarar-Fisher C, Li J, Womack ED, et al. Ketogenic regimens for acute neurotraumatic events. *Curr Opin Biotechnol* 2021; **70**: 68-74.
17. Puchowicz MA, Xu K, Sun X, et al. Diet-induced ketosis increases capillary density without altered blood flow in rat brain. 2007; **292**(6): E1607-E15.
18. Ryan KK, Packard AEB, Larson KR, et al. Dietary Manipulations That Induce Ketosis Activate the HPA Axis in Male Rats and Mice: A Potential Role for Fibroblast Growth Factor-21. *Endocrinology* 2018; **159**(1): 400-13.
19. Osimo EF, Pillinger T, Rodriguez IM, Khandaker GM, Pariante CM, Howes OD. Inflammatory markers in depression: A meta-analysis of mean differences and variability in 5,166 patients and 5,083 controls. *Brain Behav Immun* 2020; **87**: 901-9.
20. Carniel BP, da Rocha NS. Brain-derived neurotrophic factor (BDNF) and inflammatory markers: Perspectives for the management of depression. *Progress in neuro-psychopharmacology & biological psychiatry* 2021; **108**: 110-51.
21. Amodeo G, Trusso MA, Fagiolini AJN. Depression and inflammation: disentangling a clear yet complex and multifaceted link. 2017; **7**(4): 448-57.
22. Dupuis N, Curatolo N, Benoist J-F, Auvin S. Ketogenic diet exhibits anti-inflammatory properties. *Epilepsia* 2015; **56**(7): 95-8.
23. Attaye I, van Oppenraaij S, Warmbrunn MV, Nieuwdorp M. The Role of the Gut Microbiota on the Beneficial Effects of Ketogenic Diets. *Nutrients* 2021; **14**(1): 191.
24. LLP MI. Global Ketogenic Diet Food Market - Growth, Trends and Forecast (2019 - 2024). 2019. https://www.reportlinker.com/p05790924/Global-Ketogenic-Diet-Food-Market-Growth-Trends-and-Forecast.html#:~:text=The%20global%20ketogenic%20diet%20food,and%20aiding%20in%20weight%2Dloss.
25. Dietch DM, Kerr-Gaffney J, Hockey M, et al. Efficacy of low carbohydrate and ketogenic diets in treating mood and anxiety disorders: systematic review and implications for clinical practice. *BJPsych open* 2023; **9**(3): e70.
26. Cummergen K, Hannah L, Jopling L, Cameron R, Walsh C, Perez J. What outcomes matter to service users who experience persistent depression: A mixed-method narrative review and synthesis. *Journal of Affective Disorders Reports* 2022; **10**: 100431.
27. Walsh AEL, Browning M, Drevets WC, Furey M, Harmer CJ. Dissociable temporal effects of bupropion on behavioural measures of emotional and reward processing in depression. *Philos Trans R Soc Lond B Biol Sci* 2018; **373**(1742).
28. Löwe B, Unützer J, Callahan CM, Perkins AJ, Kroenke K. Monitoring depression treatment outcomes with the patient health questionnaire-9. *Medical care* 2004; **42**(12): 1194-201.
29. Valeri L, Vanderweele TJ. Mediation analysis allowing for exposure-mediator interactions and causal interpretation: theoretical assumptions and implementation with SAS and SPSS macros. *Psychol Methods* 2013; **18**(2): 137-50.
30. Jakobsen JC, Gluud C, Wetterslev J, Winkel P. When and how should multiple imputation be used for handling missing data in randomised clinical trials – a practical guide with flowcharts. *BMC Medical Research Methodology* 2017; **17**(1): 162.
31. Schulz KF, Altman DG, Moher D, the CG. CONSORT 2010 Statement: updated guidelines for reporting parallel group randomised trials. *BMC Medicine* 2010; **8**(1): 18.
32. NIHR. Research Governance Guidelines. 2019. https://www.nihr.ac.uk/documents/research-governance-guidelines/12154#Data_Monitoring_and_Ethics_Committee_(DMEC) (accessed 27/01/2022).

Section Q. Declarations and signatures of researchers

In providing signatures, the MS IDREC Secretariat will accept either:

Option 1: Email confirmations sent from a University of Oxford email address. Separate emails should be sent by each of the relevant signatories as outlined below, indicating acceptance of their responsibilities.

Option 2: That the form be fully-signed with handwritten (wet-ink) signatures. Please scan these and the rest of the form pages to create a single PDF document and email to us.

I/We, the researcher(s) agree:

- To start this research only after obtaining approval from MS IDREC/CUREC;
- To carry out this research only if funding is adequate to enable it to be carried out according to good research practice and in an ethical manner;
- That it is the responsibility of the Principal Investigator to ensure that all researchers working on this project are qualified and either experienced, or have received appropriate ethical training, to conduct the research described;
- To provide additional information as requested by MS IDREC/CUREC before approval is secured and as research progresses;
- To maintain the confidentiality of all data collected from or about participants;
- To notify the MS IDREC in writing immediately of any proposed change which would increase the risks that any participant is exposed to and await approval before proceeding with the proposed change;
- To notify the MS IDREC if the Principal Investigator on the research changes and supply the name of the successor;
- To notify the MS IDREC in writing within seven days if any serious *adverse event* occurs in the course of research;
- To use data collected only for the research for which approval has been given;
- To grant access to data only to authorised persons; and
- To maintain security procedures for the protection of personal data, including (but not restricted to): removal of identifying information from data collection forms and computer files, storage of linkage codes in a locked cabinet and password control for access to identified data on computer files.

**Principal Investigator (Name)**	Min Gao Paul Aveyard
**Principal Investigator (Signature)**<br>Pasted images of signatures cannot be accepted	Min GAO Paul Aveyard
**Medically qualified collaborator (Name)**	Paul Aveyard
**Medically qualified collaborator (Signature)**<br>Pasted images of signatures cannot be accepted	Paul Aveyard
**Student (Name)**	
**Student (Signature)**<br>Pasted images of signatures cannot be accepted	

**Section R. Acceptance by Head of Department/Faculty*** *or other senior member of the department if the Principal Investigator is the Head of Department. Example nominees include Deputy Head of Department, or, for student projects, Director of Graduate Studies.	
• I have read this application, and am aware of the research proposed.<br>• To the best of my knowledge, the proposed design and scientific methodology do not raise concerns.<br>• I support this research in principle, subject to ethical and other necessary reviews.	
**Head of Department (Name)**	
**Head of Department (Signature)**<br>Wet-ink signature (not pasted electronic image)<br>*or*<br>The Head of Department/nominee can send an email (including PI name and study title) to ethics@medsci.ox.ac.uk confirming the above	
**Date**	

Section S. Amendment history

List details of all protocol amendments here whenever a new version of the protocol is produced.

This is not necessary prior to initial ethics submission.

Amendment No.	Protocol Version No.	Date issued	Author(s) of changes	Details of Changes made

CENTRAL UNIVERSITY RESEARCH ETHICS COMMITTEE (CUREC)

AMENDMENT FORM FOR MS IDREC-APPROVED STUDIES

Please complete electronically and submit by email with supporting documents. Where possible, provide word versions of all revised documents showing the amendments (old and new text) as tracked changes.

Please note that the MS IDREC do not need to be informed of the addition of a new member of the research team to a study, unless it is to appoint a new Principal Investigator (PI) or to add an undergraduate student researcher for the purpose of their BA Experimental Psychology or BA Psychology, Philosophy and Linguistics research. However, if your study required Health Research Authority (HRA) approval due to involvement/use of NHS staff or NHS premises, then you must still notify the Sponsor - by submitting a minor amendment form, CV(s) for new research team member(s) and any other modified study documentation for Sponsor review and then to the HRA for approval. Details for HRA submission will be provided following Sponsor review and authorisation.

**Principal Investigator**	
Name	Min Gao
Name of student *(if student research)*	
Department or Institute	Primary Care department
University e-mail address	min.gao@phc.ox.ac.uk

**Study Details**	
Full title of research study	Dietary Interventions for MEntal Health in People with Treatment-Resistant Depression
CUREC ethics approval reference	R87397/RE001
Sponsor's reference number (PID, *where applicable)*	National Institute of Health Research (NIHR) Oxford Health Biomedical Research Centre (BRC)
Number of previous amendments submitted since original approval	None

**Documents Submitted for Review**			**New Version No.**
Updated CUREC form *(required for most amendments)*	YES ☒	NO ☐	N/A
Revised Advertising Material	YES ☐	NO ☒	
Revised Information sheet(s)	YES ☐	NO ☒	

Documents Submitted for Review			New Version No.
Revised Consent form(s)	YES ☐	NO ☒	
Other revised documents *(please list)*	YES ☒	NO ☐	APPENDIX 16. Qualitative interview questions
Email / Letter from Sponsor approving this amendment *(if applicable)*	YES ☐	NO ☒	N/A
New documents *(please list)*	YES ☐	NO ☒	

Main Changes Proposed in this Amendment

Please list the changes proposed with reasons for making the change in the table below. Refer to the examples provided at the top of the table and add further lines if required.
There is no requirement to detail the exact changes made to each document, since these will be shown as tracked changes on the document.

Proposed Change	Reason for Request
Example: Extension of REC approval from current end date of 31 Dec 2017 to 31 Dec 2018	*Example: Recruitment has been slow*
Example: Addition of a new sub-study	*Example: Findings to date have been interesting and we would like to include further investigations to corroborate our data*
1. Replace “registered dietitian” with “supervised by a dietitian”	There are three reasons for this change: first, we are short of registered dietitians to deliver support calls, so our registered dietitians will arrange training for new researchers. These researchers will initially deliver the calls under the supervision of our registered dietitians to ensure quality. They will conduct the calls independently but will seek advice from registered dietitians as needed.<br>Second, our support calls do not involve extensive nutritional advice, and our dietitians have collected and prepared common questions with correct answers, so this change will not affect the integrity of the intervention.<br>Third, this change is safe for our participants because patients with special dietary requirements or bowel issues were not included in this study.
2. We have revised “APPENDIX 16. Qualitative interview questions” to use more conversational language and added further questions about patients' experiences and attitudes towards the meals.	This change helps researchers follow the process more easily, facilitates our conversations with participants, and increases our understanding of how patients feel about the entire study.

STATISTICAL ANALYSIS PLAN

Version number and date: 4.0, 12 December 2024

A ketogenic diet as an additional treatment to antidepressants in people with treatment-resistant depression: a randomised, placebo-controlled trial (DIME)

Version history

Version:	Version Date:	Changes:
4.0	12 December	Final version
3.0	28 November 2024	Add "5. SECONDARY ANALYSIS"
2.0	30 October 2024	Update the CONSORT flow diagram
1.0	9 September 2024	Addition of information on data analysis

TABLE OF CONTENTS

1. INTRODUCTION 3
   1.1. PREFACE 3
   1.2. PURPOSE AND SCOPE OF THE PLAN 3
   1.3. TRIAL OVERVIEW 3
   1.4. OBJECTIVES AND OUTCOME MEASURES 3
2. TRIAL DESIGN 4
   2.1. TARGET POPULATION 5
   2.2. SAMPLE SIZE 6
   2.3. RANDOMISATION AND BLINDING IN THE ANALYSIS STAGE 6
3. ANALYSIS – GENERAL CONSIDERATIONS 6
   3.1. DESCRIPTIVE STATISTICS 6
   3.2. DEFINITION OF POPULATION FOR ANALYSIS 6
4. PRIMARY ANALYSIS 7
   4.1. PRIMARY OUTCOMES 7
   4.2. SECONDARY OUTCOMES 7
   4.3. EXPLORATORY AND DESCRIPTIVE OUTCOMES 7
   4.4. PROCESS OUTCOMES 8
   4.5. HANDLING MISSING DATA 9
   4.6. HANDLING OUTLIERS 9
   4.7. MULTIPLE COMPARISONS AND MULTIPLICITY 9
5. SECONDARY ANALYSIS 9
6. SUBGROUP ANALYSES 9
7. MECHANISTIC ANALYSES 9
8. ADDITIONAL EXPLORATORY ANALYSIS 9
9. ADVERSE EVENTS 10
10. VALIDATION 10
11. PROTOCOL AMENDMENTS RELEVANT TO THE ANALYSIS PLAN 10
12. REFERENCES 10

1. INTRODUCTION

1.1. PREFACE

The Chief Investigator will run the statistical analysis. The investigators and the Trial Statistician have reviewed and approved the statistical analysis plan. The current SAP will support trial protocol [1].

Data will be entered and analysed in Stata 16.

We will aim to follow this SAP unless it is not appropriate to do so and will report deviations from the SAP in the publication.

1.2. PURPOSE AND SCOPE OF THE PLAN

The purpose of the plan is to describe the analysis of the primary, secondary, exploratory and mechanistic outcome measures as stated in the protocol. No interim analysis is planned.

1.3. TRIAL OVERVIEW

Please see sections 1 (background) and 2 (methods and analysis) of the protocol [1].

1.4. OBJECTIVES AND OUTCOME MEASURES

Table 1 lists the primary and secondary outcome measures, an exploratory outcome measure, and mechanistic outcome measures. Table 1 corresponds to the primary, secondary and mechanistic outcome measures listed in Table 2 of the published protocol [1]. Exploratory and process outcome measures are now listed in separate sections below.

Table 1 Objectives and outcome measures

	Objectives	Outcome Measures	Time point(s)
**Primary**	Self-reported depression severity at post-treatment (week 6)	PHQ-9	Weeks 6
**Secondary**	Self-reported depression severity during follow-ups	PHQ-9	Baseline, weeks 2, 4, 6, 12
**Secondary**	Remission of depression	PHQ-9 score ≤ 4	Baseline, weeks 6
**Secondary**	Anxiety	GAD-7	Baseline, weeks 2, 4, 6, 12
**Secondary**	The inability to experience pleasure (i.e. anhedonia)	SHAPS	Baseline, week 6, 12
**Secondary**	Cognitive functioning	PDQ-5	Baseline, week 6, 12
**Secondary**	Health-related quality of life	SF-12	Baseline, week 6, 12
**Secondary**	Functional outcome	WSAS	Baseline, week 6, 12

**Mechanistic**	Changes in cortisol level for awakening response	Saliva sample (home test kits)	Baseline, week 6
**Mechanistic**	Changes in gut microbiome for all species	Stool samples (home test kits)	Baseline, week 6
**Mechanistic**	Changes in reward sensitivity	PILT	Baseline, week 6, 12
**Mechanistic**	Changes in the inability to experience pleasure (i.e. anhedonia)	SHAPS	Baseline, week 6, 12

Note: PHQ-9, Patient Health Questionnaire-9; GAD-7, General Anxiety Disorder Scale; SHAPS, Snaith-Hamilton Pleasure Scale; PDQ-5, Perceived deficits questionnaire-5-item; SF-12, SF Short-Form 12 Health Survey; WSAS, Work and Social Adjustment Scale; PILT, Probabilistic instrumental learning task.

2. TRIAL DESIGN

Figure 1 shows the CONSORT Flow Diagram of our parallel randomized controlled trial, as described below.

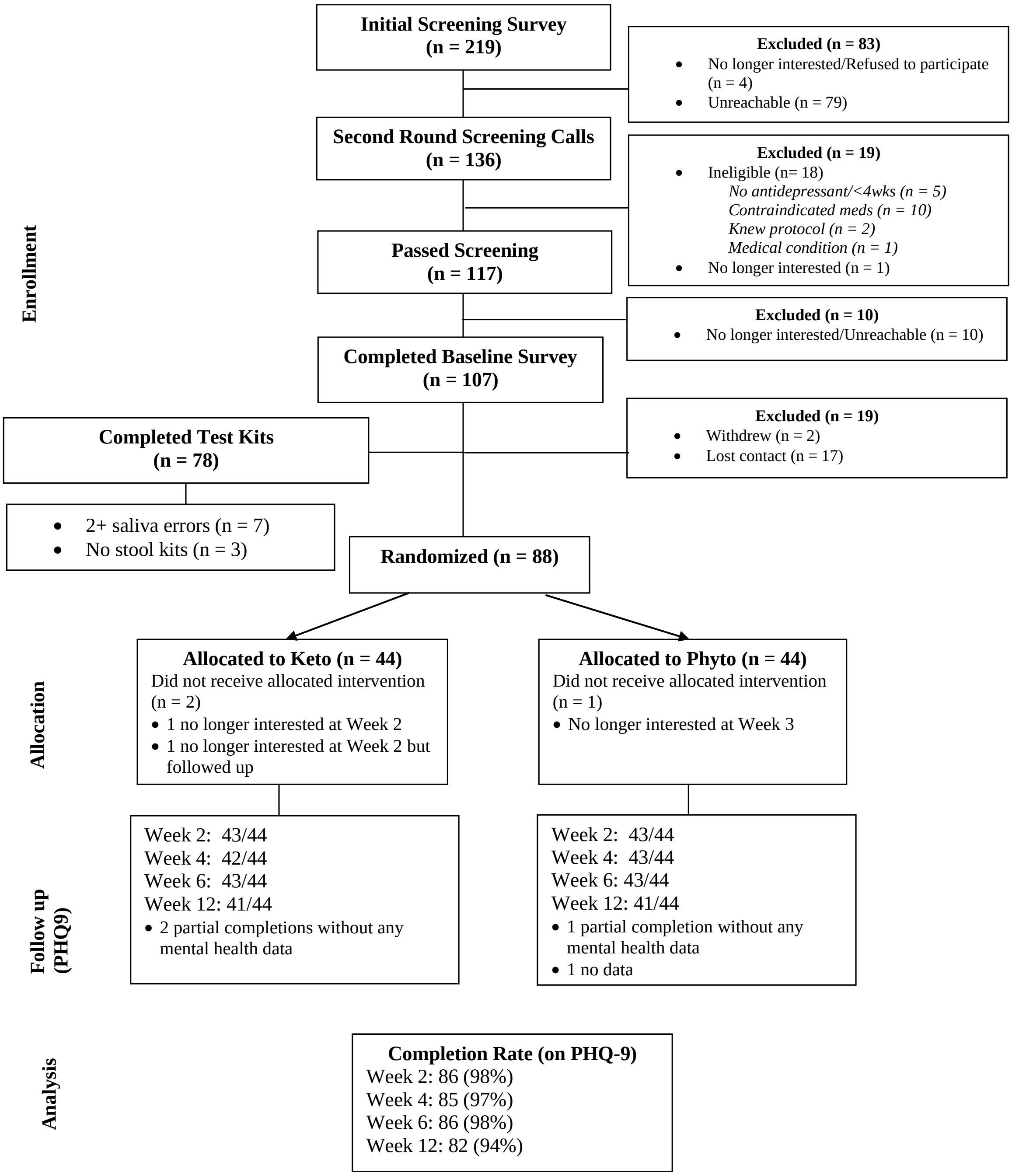

Figure 1 CONSORT Flow Diagram

2.1. TARGET POPULATION

Eligible participants are adults aged 18-65 years with depression, who have been treated with at least two antidepressants in the current episode but still have moderate or severe depression (≥15) as assessed by PHQ-9.

Detailed inclusion/exclusion criteria are listed in the protocol.

2.2. SAMPLE SIZE

We consider a 5-point difference in PHQ-9 total score (0-27 scale) to represent a minimum clinically important difference, as suggested by Lowe et al [2] This study reported that the SD of the change in PHQ-9 over 3 months as 5.8 and as 6.1 over 6 months. Assuming an SD of 6 over 6 weeks would suggest a standardised effect size of 0.83 in PHQ-9 to be clinically relevant. The sample size to test differences between groups at 90% power and at a type one error rate of 5% would be 64 (32 per group). A sample size of 100 participants will be recruited in case of 35% attrition/missing data. In simulations we confirmed that this sample size remains robust under scenarios such as unequal sizes of the strata used in minimisation. In actuality, recruitment was stopped when 88 people were randomised because it was clear that follow-up rates were substantially higher than predicted, meaning that we were on track to achieve more than 64 people contributing to the primary outcome and achieving sufficient power.

2.3. RANDOMISATION AND BLINDING IN THE ANALYSIS STAGE

With minimisation (based on BMI [less than 30 kg/m2 vs. 30+ kg/m2] and PHQ-9 [15-19, moderately severe] vs. [20-27, severe]) and 1:1 ratio. Details including the allocation concealment mechanism are described in the 2.6 section of the protocol.

3. ANALYSIS – GENERAL CONSIDERATIONS

3.1. DESCRIPTIVE STATISTICS

A table will present the baseline characteristics. This table will include depression severity (continuous), duration of current depression (continuous), current antidepressant medication use (categorical), all secondary outcome scores (continuous), age (continuous), sex (categorical), ethnic group (categorical), education (categorical), area of deprivation (categorical), BMI (continuous), smoking status (categorical), alcohol consumption (continuous), illicit drug use (binary), and health conditions (T2DM, hypertension, CVD). Continuous variables will be summarised using means and standard deviations. Medians with interquartile ranges will be presented where appropriate. Categorical variables will be summarised using frequency counts and percentages.

3.2. DEFINITION OF POPULATION FOR ANALYSIS

Primary analysis: Given the nature of the study, all randomised participants with at least one measurement on the outcome of interest will be included in the primary intention-to-treat analysis in the group they were randomised.

Safety analysis (serious adverse event): This will include participants who commenced the ketogenic diet compared with all those who commenced the Phyto diet.

The per-protocol population is characterised by:

- health coaches selecting 'Completely/Every Day' or 'Mostly/Most of the Days', when assessing participants' adherence to the diet, in at least 5 out of the (up to) 6 follow-

up calls. (Note on missing data: participants responding to fewer than 5 calls will not considered adherent)

4. PRIMARY ANALYSIS

4.1. PRIMARY OUTCOMES

The change in PHQ-9 scores from baseline to week 6 will be compared between groups. Initially, data will be plotted in the following tables. A linear contrast will be used to test the difference between groups in changes from baseline to week 6 in PHQ-9 scores. Two-tailed tests and a significance level of 0.05 will be used.

The statistical analysis of the primary outcome, effectiveness of the intervention for weight loss, will be carried out both on the basis of intention-to-treat (ITT) and per-protocol (PP). For the ITT analysis, participants will be analysed according to their allocated intervention group. For the per-protocol analysis, we will include participants who, in at least 5 out of the 6 follow-up calls, were rated by health coaches as adhering to the diet at levels of "Completely/Every Day" or "Mostly/Most of the Days".

In both cases, a linear mixed effect model that includes treatment group, time (categorical) and their interaction as fixed effects, and individual subjects as random effects will be fit using PHQ-9 at all available assessments. We will include stratification variables (BMI values and PHQ-9) as continuous variables.

The primary and secondary treatment effects will be given by the interaction between timepoint and treatment. We will present the outcome in the original units of the scale and calculate Cohen's d.

Normality of residuals will be checked graphically. If there are substantial violations from normality in the primary analysis then a non-parametric *p*-value will be calculated by permutation testing. For binary outcomes, we will use analogous generalised linear models.

4.2. SECONDARY OUTCOMES

Proportion achieving remission (PHQ9<=4) will be analysed using a mixed effect logistic regression model. If convergence fails, then week 6 and week 12 will be analysed as separate logistic regression models.

Secondary outcome measures will be analysed using analogous models to the primary outcome. This will include linear models adjusting for treatment group, baseline value of the dependent variable, and the stratification factors (BMI and PHQ-9).

4.3. EXPLORATORY AND DESCRIPTIVE OUTCOMES

The following exploratory outcomes will be reported descriptively. To facilitate interpretation, we propose to analyse weight change, rather than BMI change in the protocol given those two

are exactly equivalent. In the protocol, dietary adherence was listed as an exploratory outcome, but here we classify it as a process outcome (Section 4.4 below).

Objectives	Objectives	Outcome Measures	Time point(s)
**Exploratory**	Change in weight	Self-measured	Baseline, weeks 6, 12
**Exploratory**	Alcohol use	AUDIT	Baseline, week 12*
**Exploratory**	Smoking	Self-reported	Baseline, week 6, 12
**Exploratory**	Illicit drug use	Self-reported	Baseline, week 6, 12
**Exploratory**	Being off work sick	Self-reported	Baseline, week 6, 12

* For alcohol, week 6 is omitted here, since at week 6 abstinence from alcohol is a component of adherence to the ketogenic diet.

4.4. PROCESS OUTCOMES

The following outcomes will be presented descriptively

Objectives	Outcome Measures	Time point(s)
**Intervention engagement**	Proportion of sessions attended (Numbers of attended/6)	Baseline, week 6
**Dietary adherence score**	The percentage of health coaches selecting 'Completely/Every Day' or 'Mostly/Most of the Days' when assessing participants' adherence to the diet.	Weeks 1, 2, 3, 4, 5, 6
**Ketone levels**	- the percentage of achieving ketosis level of 1.5 mmol/L and above<br>- the percentage of achieving ketosis level of 4 mmol/L and above<br>- the percentage of achieving ketosis level of median mmol/L and above	Weeks 1, 2, 3, 4, 5, 6
**Diet adherence after intervention**	Self-reported adherence to KD at each time point "Are you still following the diet that the dietitian advised you to follow?"	Week 12
**Satisfaction with the intervention**	Proportion of diet and study satisfaction (e.g. I have enjoyed the food I have eaten over the past six weeks, how was your experience of the diet and support you got)	Weeks 6

4.5. HANDLING MISSING DATA

The primary analysis is a mixed effects model using available case analysis. This is valid under a missing at random assumption (MAR).

Sensitivity analyses will include assumptions that those with missing data have either severe depression (PHQ-9 score 20), worst case depression (PHQ-9 score 27), unchanged score with baseline observation carried forward, unchanged score with last observation carried forward, or have achieved remission (PHQ-9 score 4).

4.6. HANDLING OUTLIERS

For the analysis of the primary outcome, we do not expect significant outliers based on our definition of population for analysis. Data outliers will be defined as being at least three standard deviations from the mean of its distribution in the variable at that time-point. We will investigate and report the reasons for outliers. Outliers will be included in the analysis and a sensitivity analysis will be conducted by setting outliers to be missing.

4.7. MULTIPLE COMPARISONS AND MULTIPLICITY

As the comparisons have been pre-specified, we will not correct for multiple testing [3]. The level of statistical significance will be set at $p<0.05$.

5. SECONDARY ANALYSIS

Analysis of the primary analysis population will be repeated with additional adjustments for age, sex and presence of comorbidities (T2DM, hypertension, CVD).

6. SUBGROUP ANALYSES

Pre-specified subgroup analyses of the primary outcome will be explored by baseline depression severity (PHQ-9 score: 15-19 [moderately severe] vs. 20-27 [severe]) and duration of depression at baseline split at the median. We will elaborate the primary outcome model by adding a three-way interaction term between trial arm, time, and the subgroup term. We will include all possible two-way interaction terms in this model. As these models are exploratory, we will in any case present treatment effects for each subgroup.

7. MECHANISTIC ANALYSES

If results from primary analyses warrant further investigation, a further analysis plan for analyses of mediation and mechanistic variables will be prepared with a view to a further publication.

8. ADDITIONAL EXPLORATORY ANALYSIS

We have not yet planned any additional exploratory analysis.

9. ADVERSE EVENTS

Only a serious adverse event (SAE) occurring to a participant will be collected in this study. Serious adverse events will be presented descriptively

- classified by types,
- the relevance of this SAE to the treatment

We will report serious adverse events (SAEs) only, reporting the total number and the total number of people affected by the arm. We expect these to be very rare and will not use inferential statistics.

10. VALIDATION

The trial statistician has reviewed the analysis plan as well as the code and output of the primary outcome measures.

11. PROTOCOL AMENDMENTS RELEVANT TO THE ANALYSIS PLAN

We added the following outcomes since publication of the trial protocol [1]: the PILT task would also be conducted 12-weeks after randomisation.

12. REFERENCES

1. Gao M, Kirk M, Lash E, et al. Evaluating the efficacy and mechanisms of a ketogenic diet as adjunctive treatment for people with treatment-resistant depression: A protocol for a randomised controlled trial. *Journal of Psychiatric Research* 2024; **174**: 230-6.
2. Löwe B, Unützer J, Callahan CM, Perkins AJ, Kroenke K. Monitoring depression treatment outcomes with the patient health questionnaire-9. *Medical care* 2004; **42**(12): 1194-201.
3. Li G, Taljaard M, Van den Heuvel ER, et al. An introduction to multiplicity issues in clinical trials: the what, why, when and how. *Int J Epidemiol* 2017; **46**(2): 746-55.
