# Supplementary material for: A Ketogenic Diet for Treatment-Resistant Depression: A Randomized Clinical Trial
Source: JAMA Psychiatry. 2026 Feb 4;83(4):331–40. doi: 10.1001/jamapsychiatry.2025.4431 (PMC12874075; doi:10.1001/jamapsychiatry.2025.4431)
Supplement: Supplement 2. — eMethods. eFigure 1. Geographic spread of participants enrolled in the DIME Study based on targeted recruitment eFigure 2. The percentage of participants in the KD group measuring urine ketones each week eFigure 3. Association between mean values of ketone concentration and change in PHQ-9 score from baseline to week 6 eFigure 4. Association between the proportion of mean values of ketone concentration above 1.5 mmol/L with change in PHQ-9 score from baseline to week 6 eFigure 5. Association between the proportion of mean values of ketone concentration above 4 mmol/L with change in PHQ-9 score from baseline to week 6 eTable 1. Primary outcome by group allocation and per protocol eTable 2. Sensitivity analysis outcomes by group allocation at week 6, of participants assigned to the KD group (n=44) or Phyto group (n=44) eTable 3. Secondary analyses by group allocation with adjustments for age, sex and presence of comorbidities (T2DM, hypertension), of participants assigned to the KD group (n=44) or Phyto group (n=44) eTable 4. Subgroup analysis outcomes by baseline depression severity (PHQ-9 scores of 15-19 vs. 20-27) and depression duration at baseline (median and above, vs. below median) eTable 5. Secondary outcomes by group allocation, of participants assigned to the KD group (n=44) or Phyto group (n=44) eTable 6. Exploratory outcomes by group allocation, of participants assigned to the KD group (n=44) or Phyto group (n=44) eTable 7. Demographics of 20 participants who participated in qualitative interviews eTable 8. Individual-level demographics of participants who participated in qualitative interviews (n = 20) eTable 9. Qualitative evidence synthesis from content analysis: Categories and data relating to perceptions and experiences of the intervention programme [file jamapsychiatry-e254431-s002.pdf]

Supplemental Online Content

Gao M, Kirk M, Knight H, et al. A ketogenic diet for treatment-resistant depression: a randomized clinical trial. *JAMA Psychiatry*. Published online February 4, 2026. doi: 10.1001/jamapsychiatry.2025.4431

eMethods.

eFigure 1. Geographic spread of participants enrolled in the DIME Study based on targeted recruitment.

eFigure 2. The percentage of participants in the KD group measuring urine ketones each week.

eFigure 3. Association between mean values of ketone concentration and change in PHQ-9 score from baseline to week 6.

eFigure 4. Association between the proportion of mean values of ketone concentration above 1.5 mmol/L with change in PHQ-9 score from baseline to week 6.

eFigure 5. Association between the proportion of mean values of ketone concentration above 4 mmol/L with change in PHQ-9 score from baseline to week 6.

eTable 1. Primary outcome by group allocation and per protocol.

eTable 2. Sensitivity analysis outcomes by group allocation at week 6, of participants assigned to the KD group (n=44) or Phyto group (n=44).

eTable 3. Secondary analyses by group allocation with adjustments for age, sex and presence of comorbidities (T2DM, hypertension), of participants assigned to the KD group (n=44) or Phyto group (n=44).

eTable 4. Subgroup analysis outcomes by baseline depression severity (PHQ-9 scores of 15-19 vs. 20-27) and depression duration at baseline (median and above, vs. below median).

eTable 5. Secondary outcomes by group allocation, of participants assigned to the KD group (n=44) or Phyto group (n=44).

eTable 6. Exploratory outcomes by group allocation, of participants assigned to the KD group (n=44) or Phyto group (n=44).

eTable 7. Demographics of 20 participants who participated in qualitative interviews.

eTable 8. Individual-level demographics of participants who participated in qualitative interviews (*n* = 20).

eTable 9. Qualitative evidence synthesis from content analysis: Categories and data relating to perceptions and experiences of the intervention programme.

This supplemental material has been provided by the authors to give readers additional information about their work.

© 2026 Gao M et al. *JAMA Psychiatry*.

eMethods

Setting and participants

The study was promoted via social media advertisements. Study advertising was targeted to reach a balanced representation across age, gender, ethnic groups, and socioeconomic backgrounds.

Potential participants completed an anonymous online screening questionnaire and if eligible, they were invited to provide electronic written consent. Potentially eligible individuals were invited to a telephone assessment to evaluate their usual diet, current antidepressant use, other medical conditions diagnosed by a physician, and the presence of psychotic symptoms or suicide risk to confirm eligibility.

Patient and Public Involvement

Three group interviews with a 12-member lived-experience patient advisory group were conducted, informing the study's development, design, implementation, results interpretation, and dissemination. Their ongoing contributions aimed to deliver a study that was patient-centered and addressed priorities meaningful to those with lived experience. Further details of the patient involvement were outlined here [1].

Exploratory analyses

Mean weight change was similar and modest, in both groups as intended (eTable 6). Alcohol consumption measured by AUDIT-C scores slightly increased by week 12. Two KD participants started smoking after intervention but 1 Phyto participant stopped smoking. No participants stopped illicit drug use during the study. At week 12, one fewer participant in the KD group and one more in the Phyto group were off work sick compared to baseline.

Adverse events

Only serious adverse events (SAEs) were collected, defined as those resulting in death, being life-threatening, requiring hospitalization or its prolongation, causing significant disability, or involving a congenital anomaly. There were no serious events related to the research procedures.[2]

Motivation to participate in the study

Participants' reasons to join the DIME study varied. Many expressed their desire to reduce or stop their long-term antidepressant medication use, a curiosity to experiment with diet for their mental health symptoms, and felt the convenience was a motivating factor. Targeted social media ads were viewed as effective, and participants' trust in the study's academic affiliation provided reassurance of its credibility and was a motivator for people who wanted to contribute to mental health research. Additional factors such as losing weight and the study incentives were reported. Motivational factors were broadly categorized as *intrinsic* (e.g., curiosity, interest, or enjoyment) and *extrinsic* (e.g., external rewards or incentives).

Initial perceptions of KDs

Participants had heard of the ketogenic diet, notably as a weight loss strategy, but few had tried this approach. No participants were aware of the proposed benefits of a KD for mental health and were surprised or confused when learning about this. For example, one participant stated, "*I've never seen the link made between mental health and the keto diet,*" while another noted that media representations of KDs emphasized weight loss, overshadowing other potential benefits. Concerns about adopting a high-fat diet and deviating from familiar eating patterns generated initial apprehension to start the diet.

Perceptions, Experiences, and Challenges of this KD Intervention

Initial onboarding to the KD elicited mixed experiences. Structured support, including an induction consultation, educational materials, and prepared meal delivery, were reported to reduce apprehension and cognitive burden. Several participants experienced temporary physical discomfort during KD onset, including lethargy, nausea, and

© 2026 Gao M et al. *JAMA Psychiatry*.

dizziness, which negatively impacted adherence. Guidance from the study team helped with diet adjustment over time.

Participants generally perceived the KD program as demanding and restrictive. Those who persisted reported it as rewarding in the short term. Participants did not always adhere because they reported finding the diet restrictive, with limited variety, and experienced difficulties eliminating "comfort" foods (e.g., bread), temptations in social settings (e.g., restaurants), logistical constraints at work (e.g., no microwave or refrigeration), and financial concerns of purchasing keto-friendly foods without support. Emotional difficulties were reported, such as mood fluctuations, confusion over energy-restricted prepared meals, and intense guilt after a dietary lapse. Three people from ethnic minority backgrounds reported that they nearly gave up the diet because the supplied ketogenic food was not tailored to their cultural food preferences. Customized dietary guidance to help participants purchase their own groceries was provided, helping two participants maintain adherence during the 6-week intervention. Participants believed that a better choice of ketogenic-compliant food and advice on mitigating side effects would enhance adherence.

Participants reported that the supplied meals, dietary guidance on alternatives (e.g., keto-friendly pasta), and social support were important to foster adherence. While educational materials were highlighted as initially useful, they were not read after the first week and people mostly valued the non-judgmental and personalized in-person support. Strategies such as pre-planning restaurant meals and using ketosis urine strips to monitor progress were reported to enhance adherence.

Post-Intervention Continuance and Long-term Adherence

Following the end of the six-week program, most participants discontinued the KD, reporting that they found it restrictive and difficult to do without structured support and meal delivery. Some retained some KD habits, such as reduced sugar or processed food intake, or increased protein or vegetable consumption, with some having purchased cookware (e.g., spiralizer) to make keto-friendly alternatives. Some participants reported intentionally reverting to high-carbohydrate food consumption as a reward for intervention completion. One participant stated,

> *"After you haven't had sugar and carbs for six weeks, you just want to reward yourself with something. Buy pasta or, I don't know, that guilty pleasure."*

Participants reported feeling positive about weight loss, improved mood and reduced anhedonia, improved sleep quality and energy, and daily function, though these were perceived to be short-lived. One participant noted at the end of the intervention,

> *"I felt more mentally equipped to deal with the challenges of life that would probably normally have really upset me. [...] I felt I was more able to process [...] in a more rational manner."*

We did not collect data on medication use at week 12. However, a few participants reported reduced antidepressant use during the KD, with one person discontinuing medication while on the KD. Some participants reported that the restrictions of the diet made them feel stressed and worsened their mental health. While a few participants reported lasting benefits after the intervention ended, most expressed uncertainty about whether the observed changes were attributable to the KD or influenced by other external factors such as dietary support, seasonal changes, or personal life circumstances (e.g., new job, moving). Several participants expressed interest in resuming the KD for the perceived physical and emotional benefits, but desired a more flexible approach, over a strict KD protocol, to enjoy non-compliant foods. Some participants reported intense carbohydrate cravings and, in one case, a resumption in smoking that impeded long-term KD adherence.

© 2026 Gao M et al. *JAMA Psychiatry*.

Despite discontinuation of the KD, most would recommend it to others with caveats, acknowledging individual preferences. Participants were positive about the potential of a KD to improve mood and as an add-on to antidepressant medication for those motivated to commit to this strict diet but did not view it as a stand-alone replacement for treatment.

Participants recommended refining education materials, diversifying meal options to accommodate cultural preferences, and enhancing long-term support after cessation of supplied meals. They considered that tracking and self-monitoring tools incorporated into a digital platform may help support adherence.

© 2026 Gao M et al. *JAMA Psychiatry*.

eFigure 1. Geographic spread of participants enrolled in the DIME Study based on targeted recruitment

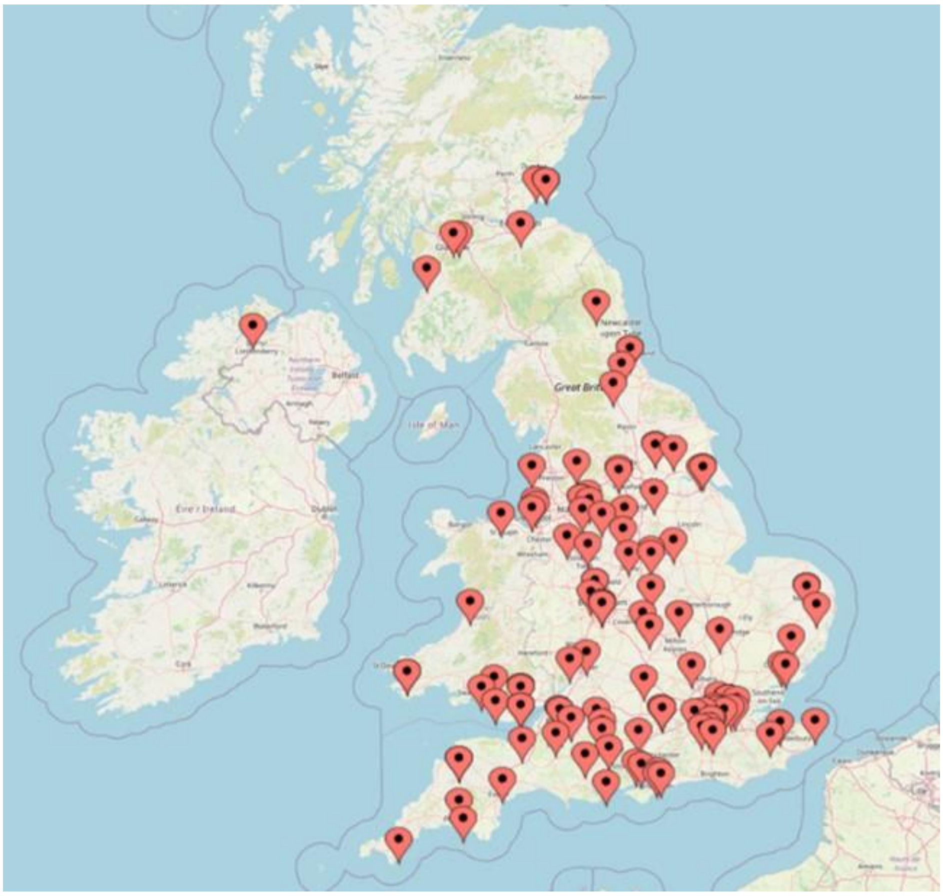

© 2026 Gao M et al. *JAMA Psychiatry*.

eFigure 2. The percentage of participants in the KD group measuring urine ketones each week

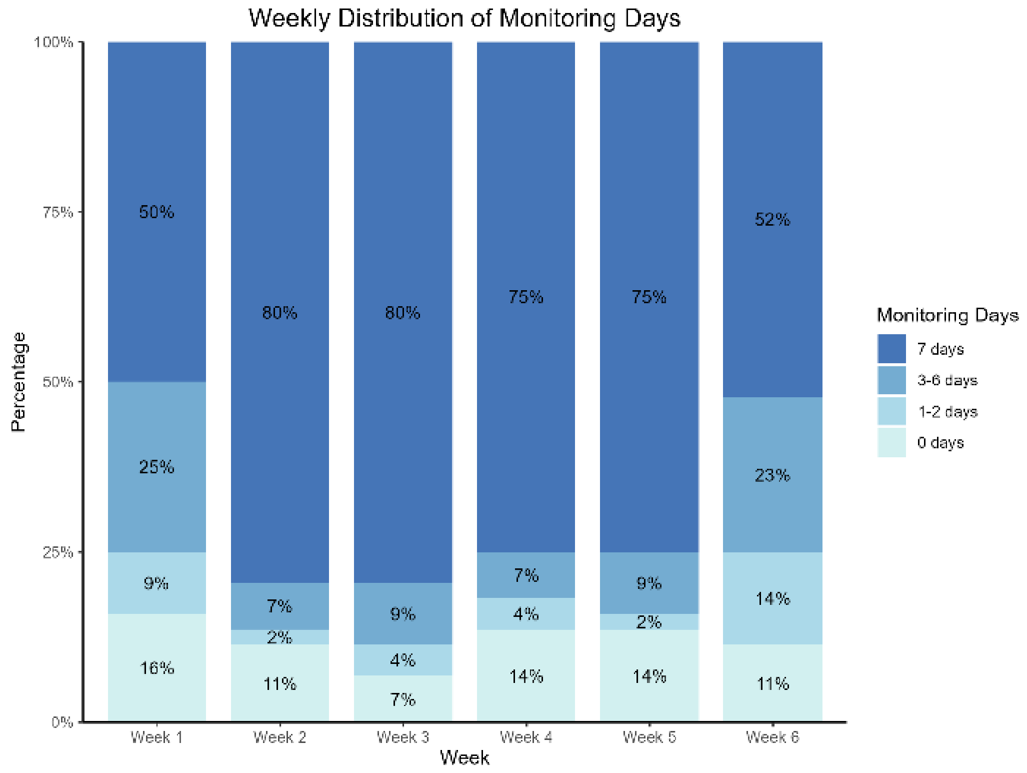

© 2026 Gao M et al. *JAMA Psychiatry*.

eFigure 3. Association between mean values of ketone concentration and change in PHQ-9 score from baseline to week 6

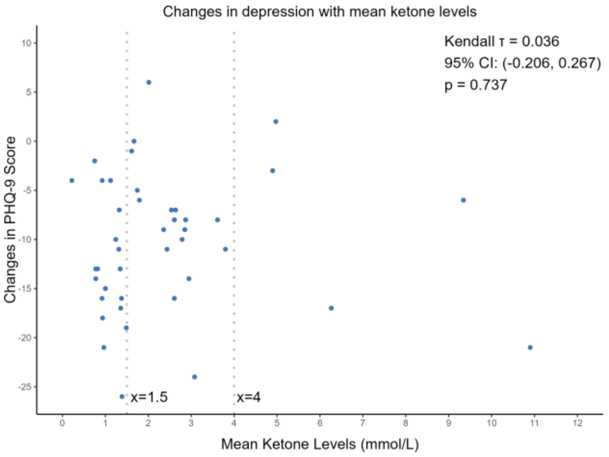

eFigure 4. Association between the proportion of mean values of ketone concentration above 1.5 mmol/L with change in PHQ-9 score from baseline to week 6

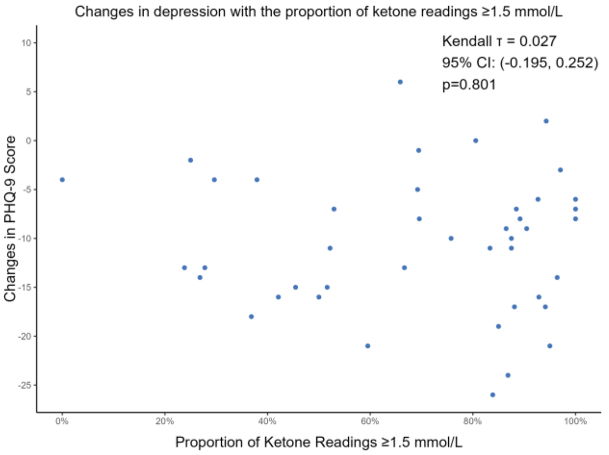

eFigure 5. Association between the proportion of mean values of ketone concentration above 4 mmol/L with change in PHQ-9 score from baseline to week 6

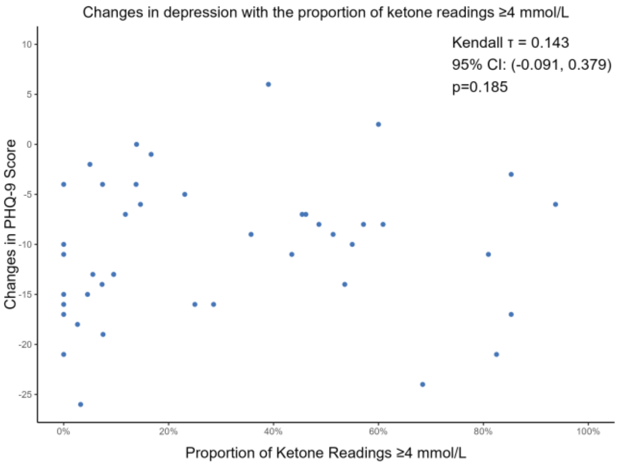

© 2026 Gao M et al. *JAMA Psychiatry*.

eTables

eTable 1. Primary outcome by group allocation and per protocol.

	KD group	Phyto group	Estimated difference (95% CI)	Estimated SMD (95% CI)	P value*
Primary outcome					
Baseline PHQ-9 (mean ± SD; n=88)	19.30 (3.22)	19.61 (3.24)			
**Group allocation:** 6-week PHQ-9 change (mean ± SD; n=86)	-10.49 (6.98)	-8.26 (5.09)	-2.18 [-4.33, -0.03]	-0.68 [-1.35, -0.01]	0.05
**Group allocation:** 12-week PHQ-9 change (mean ± SD; n=82)	-9.12(7.42)	-7.22(5.26)	-1.85 [-4.04, 0.33]	-0.58[-1.26, 0.10]	0.10
**Per protocol:** 6-week PHQ-9 change (mean ± SD; n=61)	-10.30 (7.3)	-8.30 (5.0)	-2.81[-6.08, 0.45]	-0.88[-1.89, 0.14]	0.09
**Per protocol:** 12-week PHQ-9 change (mean ± SD; n=59)	-9.16 (7.4)	-8.15 (5.1)	-1.84[-5.17, 1.48]	-0.57[-1.61, 0.46]	0.27
Note: Differences and p-values were adjusted for stratification factors (baseline BMI and PHQ-9) and for missing data; PHQ-9, Patient Health Questionnaire					

© 2026 Gao M et al. *JAMA Psychiatry*.

eTable 2. Sensitivity analysis outcomes by group allocation at week 6, of participants assigned to the KD group (n=44) or Phyto group (n=44).

Missing data on primary outcome treated as	Estimated (95% CI)	P value*
Severe Depression (PHQ-9 score 20)	-2.15[-4.39, 0.08]	0.06
Worst Case Depression (PHQ-9 score 27)	-2.18[-4.58, 0.22]	0.08
Baseline Observation Carried Forward	-2.18[-4.33, -0.03]	0.05
Last Observation Carried Forward	-2.18[-4.33, -0.03]	0.05
Achieved Remission (PHQ-9 score 4)	-2.11[-4.28, 0.05]	0.06
Note: PHQ-9, Patient Health Questionnaire		

© 2026 Gao M et al. *JAMA Psychiatry*.

eTable 3. Secondary analyses by group allocation with adjustments for age, sex and presence of comorbidities (T2DM, hypertension), of participants assigned to the KD group (n=44) or Phyto group (n=44)

	Estimated difference (KD group minus Phyto diet group) (95% CI)	Estimated SMD (95% CI)	P value*
**Depression**			
Baseline PHQ-9 (mean ± SD; n=88)			
2-week PHQ-9 change (0n=86)	-2.26[-4.42, -0.11]	-0.70[-1.37, -0.03]	0.04
4-week PHQ-9 change (n=85)	-1.17[-3.33, 0.99]	-0.36[-1.04, 0.31]	0.28
6-week PHQ-9 change (n=86)	-2.17[-4.32, -0.02]	-0.67[-1.34, -0.01]	0.05
12-week PHQ-9 change (n=82)	-1.84 [-4.02, 0.35]	-0.57[-1.25, 0.11]	0.10
Note: PHQ-9, Patient Health Questionnaire			

© 2026 Gao M et al. *JAMA Psychiatry*.

eTable 4. Subgroup analysis outcomes by baseline depression severity (PHQ-9 scores of 15-19 vs. 20-27) and depression duration at baseline (median and above, vs. below median)

Estimated difference	Baseline depression severity		Depression duration (months)	
	PHQ-9 score: 15-19 (N=46)	PHQ-9 score: 20-27 (N=42)	Below medium (N = 43)	Medium or above (N = 45)
2-week PHQ-9 change (mean ± SD)	0.140[-2.33, 2.61]	-4.93[-8.35, -1.50]	-2.94[-6.24, 0.35]	-1.48[-4.28, 1.31]
P values	0.02		0.51	
4-week PHQ-9 change (mean ± SD)	-0.24[-2.71, 2.23]	-2.23[-5.68, 1.23]	-1.85[-5.16, 1.47]	-1.43[-4.22, 1.37]
P values	0.35		0.85	
6-week PHQ-9 change (mean ± SD)	0.16 [-2.30, 2.63]	-4.73[-8.16, -1.30]	-3.88[-7.17, -0.58]	-1.39[-4.18, 1.41]
P values	0.02		0.26	
12-week PHQ-9 change (mean ± SD)	1.24[-1.27, 3.78]	-5.18[-8.63, -1.72]	-1.25[-4.09, 1.58]	-2.99[-6.33, 0.35]
P values	0.003		0.44	
Note: p-values are for a differential treatment effect between groups, calculated as a three-way interaction (see Methods); PHQ-9, Patient Health Questionnaire				

© 2026 Gao M et al. *JAMA Psychiatry*.

eTable 5. Secondary outcomes by group allocation, of participants assigned to the KD group (n=44) or Phyto group (n=44).

	KD group	Phyto group	Estimated difference or OR (95% CI)[a]	Estimated SMD (95% CI)[a]	P value*
**Depression**					
2-week PHQ-9 change (mean ± SD; n=86)	-8.81(5.98)	-6.48(5.22)	-2.27 [-4.42, -0.12]	-0.71[-1.38, -0.04]	0.04
4-week PHQ-9 change (mean ± SD; n=85)	-8.71(6.46)	-7.53(5.75)	-1.19 [-3.35, 0.97]	-0.37[-1.04, 0.30]	0.28
6-week remission of depression (n, %)	11(25.58)	4 (9.30)	6.56 [0.97, 44.62]	--	--
12-week remission of depression (n, %)	8(18.60)	4 (9.52)	3.02 [0.42, 21.47]	--	--
**Anxiety (mean ± SD)**					
Baseline GAD-7 (n=88)	12.91 (4.55)	13.64 (5.12)	--	--	--
2 week change (n=86)	-4.63(4.79)	-3.30(5.71)	-1.27 [-3.16, 0.63]	-0.26 [0.65, 0.13]	0.19
4 week change (n=85)	-5.21(5.70)	-4.00 (4.74)	-1.14[-3.04, 0.77]	-0.24 [-0.63, 0.16]	0.24
6 week change (n=86)	-6.26(5.74)	-4.77 (5.69)	-1.43 [-3.32, 0.47]	-0.30 [-0.69, 0.10]	0.14
12 week change (n=82)	-5.85(6.15)	-3.63 (5.91)	-2.02 [-3.95, -0.10]	-0.42 [-0.82, -0.02]	0.04
**Anhedonia (mean ± SD)**					
Baseline SHAPS score(n=88)	8.14(3.47)	7.86 (3.03)	--	--	--
6 week change (n=85)	-4.45(4.17)	-4.35 (3.68)	-0.07 [-1.63, 1.48]	-0.02 [-0.50, 0.46]	0.93
12 week change (n=80)	-4.68(4.13)	-3.13(4.07)	-1.48 [-3.06, 0.11]	-0.45 [-0.94, 0.03]	0.07
**Cognitive impairment (mean ± SD)**					
Baseline PDQ-5 score (n=88)	14.02(3.34)	14.70 (3.69)	--	--	--
6 week change (n=85)	-3.88(4.54)	-3.30 (4.27)	-0.52 [-2.26, 1.21]	-0.15 [-0.64, 0.34]	0.55
12 week change (n=82)	-3.76(4.67)	-2.73 (4.53)	-1.02 [-2.77, 0.74]	-0.29 [-0.79, 0.21]	0.26
**Quality of life (mean ± SD)**					
Baseline SF-12 score(n=88)	28.98(2.58)	29.16 (2.96)	--	--	--
6 week change (n=85)	-0.78(3.54)	-0.67 (3.21)	-0.11 [-1.41, 1.20]	-0.04 [-0.51, 0.43]	0.87
12 week change (n=79)	0.025(2.75)	-0.15(3.45)	0.14 [-1.20, 1.47]	0.05 [-0.43, 0.53]	0.84
**Impairment of ability to work and engage in daily activities (mean ± SD)**					
Baseline WSAS score (n=88)	25.48(7.17)	28.80 (6.72)	--	--	--
6 week change (n=85)	-8.29(8.26)	-9.63 (8.19)	1.34 [-1.89, 4.56]	-0.02 [-0.20, 0.17]	0.42
12 week change (n=80)	-8.53(8.21)	-7.49(9.40)	-1.17 [-4.47, 2.12]	0.02 [-0.17, 0.21]	0.49

[a] Adjusted for baseline value and continuous BMI value

* Omnibus likelihood ratio test: p= 0.047, indicating sufficient evidence to reject the null hypothesis that there was a trend towards a significant reduction in PHQ-9 scores at 6 weeks.

Abbreviations: PHQ-9, Patient Health Questionnaire-9

© 2026 Gao M et al. *JAMA Psychiatry*.

eTable 6. Exploratory outcomes by group allocation, of participants assigned to the KD group (n=44) or Phyto group (n=44).

	KD group	Phyto group	Total
**Weight change (kg, mean ± SD)**			
Baseline values (n=88)	90.32(17.89)	93.90(26.16)	92.11(22.35)
6-week change (n=86)	-1.38(5.28)	-1.04(7.27)	-1.21(6.31)
12-week change (n=85)	-0.57(8.05)	-0.84(6.99)	-0.70(7.50)
**AUDIT-C (mean ± SD)**			
12-week values (n=81)	2.66(1.93)	3.03(2.17)	2.84(2.05)
**Smoking (n, %)**			
6-week (n=86)			
Non-smokers	38(88%)	41(95%)	79(92%)
Light smokers	5 (12%)	2 (5%)	7(8%)
12-week (n=85)			
Non-smokers	38(88%)	40(95%)	78(92%)
Smokers	5(12%)	2(5%)	7(8%)
**Illicit drug use (n, %)***			
Baseline (n=88)			
Never	42(95%)	44(100%)	86(98%)
Monthly or less	2(5%)		2(2%)
6-week (n=86)			
Never	39(91%)	43(100%)	82(95%)
Monthly or less	4(9%)		4 (5%)
12-week (n=85)			
Never	41(95%)	42(100%)	83(98%)
Monthly or less	1(2%)	--	1(1%)
Two to four times a month	1(2%)	--	1(1%)
**Being off work sick (n, %)**			
Baseline (n=88)	11(25%)	14(32%)	25(28%)
6-week (n=86)	8(19%)	12(28%)	20(23%)
12-week (n=85)	10(23%)	15(36%)	25(29%)
*no data on other options were omitted.			

© 2026 Gao M et al. *JAMA Psychiatry*.

eTable 7. Demographics of 20 participants who participated in qualitative interviews

Demographic Variable	Value
**Age (M, *SD*)**	38.6 (12.04)
Min, Max	20,62
**Gender (n, %)**	
Female	13, 65%
Male	7, 35%
**Ethnicity (n, %)**	
Asian	6, 30%
Mixed	1, 5%
White	13, 65%
**IMD Decile (n, %)**	
Deciles 1-2 (most deprived 20%)	4, 20%
Deciles 3-5	7, 35%
Deciles 6-8	7, 35%
Deciles 9-10 (least deprived 20%)	2, 10%
**Compliance to Intervention (n, %)**	
Everyday	7, 35%
Most days	9, 45%
Some of the days	3, 15%
None/Withdrew	1, 5%

Note: IMD, Index of Multiple Deprivation.

© 2026 Gao M et al. *JAMA Psychiatry*.

eTable 8. Individual-level demographics of participants who participated in qualitative interviews (*n* = 20)

Interview ID	Sex		Age		Ethnicity		IMD decile
	M	F	<50 years	>50 years	White	Non-White	
1	✓		✓			✓	8
2		✓	✓			✓	8[a]
3		✓	✓		✓		2
4		✓	✓		✓		9
5	✓			✓	✓		5
6		✓	✓			✓	1
7		✓		✓	✓		5
8		✓	✓		✓		7
9		✓	✓			✓	6[b]
10	✓			✓	✓		4
11	✓		✓		✓		5
12		✓	✓		✓		2
13		✓	✓		✓		4
14	✓		✓			✓	2
15		✓	✓		✓		8[b]
16	✓		✓			✓	3
17		✓	✓		✓		5
18		✓	✓			✓	8
19	✓		✓		✓		10
20		✓	✓		✓		6

[a]Welsh Index of Multiple Deprivation; [b]Scottish Index of Multiple Deprivation

© 2026 Gao M et al. *JAMA Psychiatry*.

eTable 9. Qualitative evidence synthesis from content analysis: Categories and data relating to perceptions and experiences of the intervention programme

Main category	Subcategory	Component	Evidence synthesis	Example Data
**Motivation to participate**	Intrinsic	Curiosity and interest in this area of research, i.e. diet and mental health	Intrinsic motivators included curiosity, personal interest, and enjoyment in contributing to mental health research.<br><br>Participants expressed a desire to contribute to mental health research and curiosity about experimenting with the potential connection between diet and mental health benefits. Curiosity about the study's novelty and the opportunity to gain knowledge about dietary impacts on mental health were recurring reasons to take part.<br><br>Participants expressed wanting to reduce or discontinue long-term antidepressant medication use, driven by a desire to explore dietary interventions as an alternative, more holistic and less harmful approach to managing mental health symptoms.<br><br>Participants expressed the study may be an opportunity to regain personal agency over their mental health by actively engaging in self-improvement and adopting new dietary habits to manage low mood. Others viewed participation as a way to learn and explore their own mental health by understanding the mind-body connection.	*"I've never done like a Study before, so it was the first study I did […] when I was reading it I was like, yeah, this sounds like something good for me to be a part of something new to try"*<br><br>*"I went in pretty blind and I just said yes because I just thought well […] the only thing I wanted to get out of it was knowledge and see what helps."*<br><br>*"I've always had a real interest in the link between food and our bodies and food and our minds as well. So I thought it would be a really interesting study to take part in. So, yeah, I suppose just out of curiosity, really."*<br><br>*"I'm interested in diet. My diet was very heavy with sugar in particular. I liked the fact that it was quite a defined diet"*<br><br>*"it felt entirely plausible that a that a major change of diet could have a significant impact on mental health."*
		Desire to alleviate depressive symptoms<br>&<br>Alternative to medication use		*"I've always just tried medication and I've never really kind of looked at the implications of […] looking at my diet, so I thought it would be a good chance to be able to look at that to see if it did impact rather than just using medication all the time."*<br><br>*"I've been on antidepressants for quite a few years now. I don't feel like that they're working. Or at least not as well as I think that they could or should be."*<br><br>*"I do have a sort of extended history of depression, and I've tried several different drugs. None of them have ever had any kind of long lasting effects."*<br><br>*"in my mind, [I] have been on the antidepressants now for like, 10-15 years […] So I'm thinking, you know, there's got to be something else here"*<br><br>*"Mostly because I've had depression for a long time and medication is kind of OK and interesting enough, but it doesn't. Well, medication doesn't fix it."*<br><br>*"Any like treatment or medication or therapies I've had over the years don't didn't really seem to be doing anything or helping me. So I was just looking into different things to try out and see if it would help."*

© 2026 Gao M et al. *JAMA Psychiatry*.

				"the reason I guess I clicked [the study link] was because I was on antidepressants at the time, and finding that they weren't doing anything for me anymore. […] I prefer not to be highly medicated, I guess so I was very interested."
		Personal agency and sense of control		*"you're trying anything […] to try and feel a bit better because I want also to feel like you get you give yourself some agency, you know, I suppose it's that sometimes it can feel like it's out of your control and maybe trying to help yourself do something can be useful."*<br><br>*"I was thinking, oh, you know, maybe I'll try and start with the basics and look at how I'm eating and things. So I just thought, you know nothing to be lost as it were."*<br><br>*"I'm really into the brain and how it's worked and how we perceive the world. And I thought this would be a really interesting way to look at other options as well and bring them into my own. My own sort of research and theories so."*
	Extrinsic	Targeted social media advertisements	Extrinsic motivational factors for participation in the ketogenic diet study revealed tailored social media advertisements played a significant role in motivating participants to enroll.<br><br>Geographic accessibility and the convenience of a remote, non-invasive study design were reported as appealing, particularly for those that lived in rural areas, or had full-time jobs or limited time.<br><br>Trust in the study's academic affiliation provided reassurance of its credibility. Incentives such as vouchers were described as a valuable bonus, enhancing the perceived appeal of participation.<br><br>Additionally, personal struggles, such as lifelong challenges with weight management, motivated participants to explore dietary changes to improve mental health. These external factors collectively supported participants' decisions to engage with the KD and highlighted the study's accessibility, legitimacy, and appeal to a wide-range of participants.	*"I saw [the study advertisement] on the Google and I have already tried way too many antidepressants and different kind of treatments. So and it wasn't working that well."*<br><br>*"Facebook's algorithm […] was targeted at me, but I seem to fit quite well the profile. The people you were looking for."*<br><br>*"I fitted the profile of someone who might be relevant for the study, so that was it. Simple as that really."*
		Accessibility/ Convenience		*"I don't really see a lot of studies related to depression, I guess. Not in my area. […]but then this one is like it reach me on social media and it is like I could do that wherever I am in UK. So […] this one reason that encourage me to enroll."*<br><br>*"I liked that, unlike most academic studies, it didn't require any attendance or travel. It was something that I could manage at home in my own space and time"*
		Other, e.g. study credibility, incentives		*"the [study] was actually part of […] the university. It wasn't just saying, like, oh, this is accredited by blah blah blah and then it's not from that destination"*<br><br>*"the vouchers were a massive bonus. So I was thinking nice, but I wasn't expecting anything but yeah"*<br><br>*"I have always battled with my weight my whole life. […] I just thought it was a good time to try to combat my mental health and look at a changing diet"*

© 2026 Gao M et al. *JAMA Psychiatry*.

				"I feel like the incentives that are good for participants are having the food provided [and] obviously you know having vouchers. For future studies I think that's a really good way to get people on board."
**Perceptions and experiences of the dietary program**	Before starting the program	Prior knowledge of the keto diet/ expectations<br><br>*"A diet for weight loss"*	Most participants had heard of the ketogenic diet, primarily as a weight-loss strategy with an emphasis on high-fat consumption, with some associating it with specific foods like cheese, bacon, and eggs.<br><br>A small number of participants had personal experience with similar diets, such as the Atkins or Paleo diet, while others gained knowledge through friends or social media.<br><br>Participants were unaware of its potential mental health benefits, with some expressing surprise or skepticism when learning about this link.<br><br>Many participants felt apprehensive about beginning the diet due to the significant shift from their usual eating habits and lingering misperceptions about the potential health risks of consuming high-fat diets.<br><br>Media narratives focusing on weight loss and limited public understanding of the broader health applications of the ketogenic diet contributed to participants' initial hesitations and biases.	*"I heard of that [keto diet] before in the context of like. [...] Like really dieting, losing weight and stuff."*<br><br>*"I knew it was one of those diets that, you know, could possibly have beneficial effect on some health conditions. But I've never seen the link made between mental health and the keto diet."*<br><br>*"one of my friends is diabetic [..] he just generally eats a lot of bacon, a lot of eggs, a lot of high fat [...] but I hadn't heard about the benefits for mental health"*<br><br>*"I have heard about lots of people on a keto diet before, but I hadn't heard about the benefits of mental health"*<br><br>*"I didn't know much about keto other than and people would use it for weight loss, I was kind of not like in the dark but very kind of naive of what it was like."*<br><br>*"I think because like I knew people used it for weight loss. When I was told I wasn't allowed to lose weight, I was quite shocked by that"*<br><br>*"I've mostly heard of it as not used for, say, like a mental health or other health issues. It was just literally purely for like weight loss reasons that I'd like known about the diet and [...] I guess a bit of confusion as well"*<br><br>*"It being a challenge, I guess, and you know it changing a lot of my current habits and stuff like that."*<br><br>*"especially media portrayal and stuff like that. That it just has this whole connotation of weight loss around it, [...]<br>So I think it's just on a whole a biased view perhaps and the lack of more rounded education of what it can be used for."*<br><br>*"I think that it is still a lot of fear of carbohydrates for people [...] things are still low fat in supermarkets and stuff. Like the eat the fat because it's actually more beneficial for you"*
	Induction to the program	Positive experiences	Participants felt well-prepared to begin the ketogenic diet programme, supported by an induction phone call, educational materials, and meal deliveries, which eased the transition and reduced decision-making stress.	*"I think the pitch of it was great for anybody, irrespective of you know, level of education or whatever."*<br><br>*"it was like, OK, I'll just follow [...]. I don't really need to think about what I eat."*

© 2026 Gao M et al. *JAMA Psychiatry*.

				"I would say it was good overall, like once I had read it I got, I got the whole thing that OK how to do it"<br><br>*"easy to understand. Having that handbook just in case."*<br><br>*"I think I was just excited to get started with it. Really. I thought that it was clear in terms of what I was being asked to do and the different support that was available"*<br><br>*"after the first week, I was fine. I just got on with it."*
		Challenges	Significant physical discomfort during the initial induction phase, including lethargy, nausea, and dizziness associated with "keto flu," posed adherence challenges for some.<br><br>A lack of cultural dietary preferences also influenced adherence, with one participant highlighting the blandness of prepared meals compared to their South Asian cuisine.	*"in the first week, I felt horrible. I was like, sort of keto flu or something"*<br><br>*"It did mention about the keto flu, but I don't think I took it seriously. I thought maybe I won't get it or something like that, but only after I started getting those symptoms I realized OK, this is quite difficult to manage."*<br><br>*"day two and three or three and four, I fell just strained. I could hardly move my body. It's like seems to feel the symptoms of that keto flu they talk about."*<br><br>*"the initial week or so is harder, like the transition of that."*<br><br>*"the diet itself, I don't think it's very easy, especially for the first week. Yeah, was super difficult."*<br><br>*"At first it was it was quite hard not having sugar or bread"*<br><br>*"I was feeling very dizzy. I felt like I wanted to throw up and I was waiting in the hospital to meet my psychiatrist"*<br><br>*"I gave up after like a week."*<br><br>*"it got a bit difficult because the [food] was a bit different than what I'm used to because I'm South Asian with lots of spices and stuff in there is bland and I would say dry for my taste"*
	During the 6-week program	Positive experiences	Professional support from dietitians through personalized guidance, meal planning, and emotional encouragement were crucial in maintaining motivation and adherence. Structured meal deliveries and educational materials provided participants with a sense of confidence and control.	*"she guided me on what to on what I can have and stuff. So I think having the dietitian did really, it made it a lot easier and having her support."*<br><br>*"I felt incredibly motivated and committed."*<br><br>*"it was a learning curve. And yeah, I really enjoyed it."*<br><br>*"It felt that it really suited me. It felt, oh, my God, I haven't felt this good for years."*

© 2026 Gao M et al. *JAMA Psychiatry*.

		Challenges	The restrictive nature of the diet, particularly the elimination of high-carbohydrate, high-sugar comfort foods such as bread, fruit, sugar, chocolate and pasta, emerged as a significant challenge.<br><br>Physical side effects, including fatigue, muscle pain, and symptoms of keto flu, along with emotional struggles such as low mood, guilt after dietary lapses, and external stressors, further complicated adherence.	*"I found a little difficult because my diet mainly like carbs, so that would be like my comfort foods."*<br><br>*"I think I took about four weeks to actually get into ketosis properly and it was very grueling "*<br><br>*"I would say I suffered and it was very brutal on my body."*
	Keto diet	Prepared meal delivery	Many participants reported appreciating the convenience of having pre-labelled, keto-compliant ready meals, which reduced the cognitive burden of meal planning and cooking.<br><br>Some participants enjoyed the meals, while others found them unappealing and monotonous. The repetitive nature of the meals, particularly the frequent use of broccoli and cauliflower, was cited as drawback.<br><br>Participants were also confused by the calorie-restricted meals despite being advised not to lose weight.<br><br>Practical challenges were reported for those who prepared meals for family members or who worked in environments without access to a microwave or adequate food storage, making meal consumption difficult during the workday.	*"I am rubbish at knowing what to have for dinner. [...] So it was good to know that it was just set and I didn't have to think about it."*<br><br>*"I think when it's provided for you, it takes the thinking and the effort away. So I think it's far more achievable in the way that the study was run to stick to it."*<br><br>*"the broccoli was obscene as in the amount of it [...] if I was paying for that service, I would not have been happy at all because there are more vegetables you can eat on it than just broccoli."*<br><br>*"I don't think there was really a bad meal. There were meals that I loved more than others and wish that I had like an endless supply of my fridge 'cause I really enjoyed them."*<br><br>*"It was handy to have meals just delivered to you, but there was just some foods that they just did not taste nice to me."*<br><br>*"you were surprisingly satisfied after smaller portions. So I think it kind of almost re educated you that you kind of, I don't know you eat with your head, do you know what I mean?"*
		Keto-friendly snacks	Participants valued the provided keto-friendly snacks as a useful option to curb sugar cravings.<br><br>Texture was often described as off-putting, and some experienced gastrointestinal discomfort leading to lower-than-recommended consumption.	*"I found that that really affected my digestive system so I tried to reduce the amount of those that I ate because I found the effects quite physical."*<br><br>*"some of the snacks didn't agree with me as well.<br>Umm, there was one in particular that would like it went through me. maybe because it was like high in fiber and yeah, [...] I would run to the toilet"*
	Dietary advice	Weekly dietitian sessions	Participants perceived the 1:1 dietary support provided during the ketogenic diet program as the most essential aspect of the intervention.	*"I knew that I had a phone call coming up on the Monday and then I could mention anything and it would, it was almost like resetting every week"*

© 2026 Gao M et al. *JAMA Psychiatry*.

			Personalized guidance by dietitians enabled participants to adapt the diet to their individual needs, offering tailored alternatives or additions to prepared meals (e.g., creamed spinach, low-sugar milk alternatives) to enhance satisfaction and satiety.<br><br>The encouragement and motivational support from dietitians were instrumental in helping participants navigate setbacks and sustain adherence to the program.<br><br>Weekly check-ins were particularly appreciated, as they offered structure, accountability, and reinforcement of progress, contributing significantly to participants' positive experiences with the dietary advice component	*"If I went through say all physical stuff with no support, I probably would have stopped it like after a week, two weeks or something like that because that was a lot to be dealing with"*<br><br>*"she gave me plans of what to eat, and she even let me get my own food and make my own keto meals."*<br><br>*"So that was really helpful as well because I don't think I could see myself continue eating those meals because I just was not enjoying them."*<br><br>*"I was craving pasta a lot, the normal pasta. So […] She gave me some links to different sort of [pastas]…that are diet friendly"*<br><br>*"She was absolutely fantastic, sending me recipes, sending me support if I needed it. I could message or email with questions. She was really, really good."*<br><br>*"If I hadn't got that, the person to talk to, it could have been very isolating"*
		Educational written materials (e.g., leaflet)	Participants initially found the educational leaflet helpful for understanding dietary restrictions, though its use diminished after the first week.<br><br>Those who valued it highlighted the daily weight and ketone tracker and often placed it in visible locations as a reminder.<br><br>However, the leaflet's long-term use was limited, with most participants not revisiting it during the program beyond the first week.	*"Yes, that was really helpful cause it gave you the list of foods to completely avoid, which I was surprised at not being able to eat apples and grapes and bananas cause I've never even considered them as not keto friendly."*<br><br>*"It did seem like very supportive and not like shaming or anything, which is really nice. And just very like informative"*<br><br>*"I was sent like almost like a simple guide to the keto diet and that was really useful. So I've still got that and I still use that in terms of my guidebook"*<br><br>*"That guide was really helpful. […] I could look in the guide and think, OK, I can use, I can eat this instead of this or substitute this for this."*<br><br>*"I must admit I read them at the beginning and read through, read through them all, and then I didn't touch them again."*<br><br>*"I put them on a clipboard next to my desk and they stayed there for six weeks. So, but it's not that I didn't read them. And I did go through them at the beginning, but I couldn't tell you what was in them now."*<br><br>*"quite helpful to record on the food I was eating and like my weight, my keto levels."*

© 2026 Gao M et al. *JAMA Psychiatry*.

				"Yeah, I gave it a read. I think I forgot exactly how useful it was, but I don't think I got lots of information from there."<br><br>*"I only really read it at the start and then I didn't really feel the need to refer to it anymore through the diet."*<br><br>*"I was already sort of aware of the of the information that the booklet provided, so for me, that book that wasn't particularly useful."*
	Facilitators to adherence	Variety and choice of keto meals and snacks	Some participants mentioned enjoying the variety and taste of certain prepared meals, along with additional program modifications suggested by the dietitian such as alternatives which made following the diet more appealing.<br><br>Many participants expressed the value and convenience of having prepared meals supplied, which simplified diet compliance by eliminating the time and cost of extensive meal planning and reducing the risk of consuming non-compliant foods.<br><br>Regular weekly check-ins with the dietitian were highly appreciated, offering program modification guidance, accountability, and nonjudgemental support that helped participants persist through challenges.<br><br>Social support was reported to play a significant role, with participants benefiting from receiving encouragement from the study team, and friends or family members supporting the diet.<br><br>The ketosis urine strips and weekly food delivery provided structure and helped participants track and monitor their progress.<br><br>Flexible strategies such as pre-selecting restaurant menu choices with the dietitian, and supplementing the prepared meals with keto-friendly additions like nuts, creamed spinach, and avocado, also enhanced adherence.	*"I was making my own keto meals and I did actually enjoy doing that then and it gave me a lot more variety."*<br><br>*"there were definitely like things that you can add like creamed spinach and stuff. So yeah, I found it easy to stick to the to the diet."*<br><br>*"It was run very well, the delivery of food and stuff like that. And you know, you can take the choice. I don't know if there's anything else that I could have asked for."*
		Convenience		*"I felt that having the meals pre prepared was better because it would be so easy to not make that meal the way you're meant to make it and accidentally put something in that you really fancy."*<br><br>*"I think because the meals were sent to me, that helped stick to it."*<br><br>*"I think it had help in a sense of the routine of it.<br>Every Sunday I'll get a delivery. And then, yeah, these are my meals for the week"*<br><br>*"It's better than calorie counting. I mean, I've done diets in the past and never been able to stick to him, so I was amazed how I could stick to the keto."*

© 2026 Gao M et al. *JAMA Psychiatry*.

		Guided support<br><br>Social support		*"anytime I had a little bit of doubt, I knew that I had a phone call coming up on the Monday"*<br><br>*"I found it really motivational to be accountable and I think it was easier in that respect that I was accountable to somebody"*<br><br>*"She was absolutely fantastic, sending me recipes, sending me support if I needed it."*<br><br>*"I didn't really deviate much. There was a little bit, but I was hopefully I was honest with her about what I deviated on."*<br><br>*"I was very open with everything and others […] I'm not holding back anything of what I'm telling you because it's, you know, it's more beneficial for you as well. And then if you have that support with you, it just makes it so much easier"*
		Self-monitoring		*"writing everything down and doing the daily urine tests as well kept me on track quite a bit more"*<br><br>*"I kind of became my own personal kind of vendetta against those keto sticks to try and be in ketosis no matter what. So I did find that I was. It came at a really good time for me to try to be motivated and keep motivated and stick to the diet."*
		Flexibility / Adaptability		*"it actually wasn't as restrictive as I thought it was gonna be like for social events […]I could email her the menu and we could go through, you know, OK, what's the best approach for this"*<br><br>*"I basically split my snacks into like a box at home and a box at work because it just made life easier."*
	Barriers to adherence	Cravings/ Temptation	Adherence to the ketogenic diet was hindered by several interrelated barriers, including psychological, social, sensory, and logistical challenges.<br><br>Participants commonly reported intense cravings for non-keto foods, particularly carbohydrates and sugar, which were perceived as a natural response to the dietary restrictions and an urge for self-reward.	*"It felt like my mind was always craving something else."*<br><br>*"after you haven't had sugar and carbs for six weeks or maybe a little bit longer. You just kind of almost want to reward yourself with something."*
		Social and family factors		*"I have a wife and three small kids, and for me to manage a separate diet to everybody else. I was pretty sure was going to be very difficult and it was difficult"*

© 2026 Gao M et al. *JAMA Psychiatry*.

			Social dynamics, such as managing a separate diet within a household or navigating non-compliant family environments, exacerbated difficulties, leading to feelings of isolation and temptation.<br><br>Experiences including fatigue from repetitive meals, a lack of perceived variety, and aversions to the richness of high-fat meals, further diminished enthusiasm for the diet.<br><br>Logistical obstacles, such as limited food preparation options while travelling, lack of time, and the cost of keto-compliant foods, compounded adherence issues.<br><br>Psychological strain, including guilt over lapses in adherence and low mood associated with perceived failure, further demotivated participants. Collectively, these barriers illustrate the complexity of maintaining a ketogenic diet over the long term.	*"Because no one in my family's following a keto diet, so there's like stuff in the household that I'm like, Oh my God, I love that kind of food"*<br><br>*"probably most challenging in terms of the diet would be the unexpected events or invitation somewhere."*
		Flavor and/or lack of variety of the keto meals		*"we cook and we like flavor […] it was a real shock of they're just not being flavor in anything."*<br><br>*"as time went by I found that I was wanting to avoid the meals. I was skipping them because I just couldn't face another meal. They started to all taste the same to me and I started really craving fresher foods."*<br><br>*"I think it probably after the 4th week that I started to think everything tasted the same."*<br><br>*"at first I like some of the recipes, I'm just trying and you that fatty element was starting to really turn me off food and I found I wasn't looking forward to dinner."*<br><br>*"having that routine of, yeah, here or your meals, you eat this that was good but especially by the end of it, I was just very tired of eating the same thing, like over and over again"*
		Practical challenges or interference with everyday life		*"there were certain days where like I would be away for work and, you know, there wasn't a microwave."*<br><br>*"if you work from an office space or you didn't have access to a microwave or you didn't have access to a fridge to keep things cold, or if you were going out for the day for a lovely long walk, or you were going to the beach or something, they weren't, there wasn't really any options"*
		Emotional and psychological barriers		*"towards the end I did like lose like track a bit […] and I felt so guilty […] my mood was so low because of it, because I was like, I failed the whole thing. And I've just ruined everything."*<br><br>*"I had a really hard time weighing myself and then giving that information up, but I think that comes more from a long time of growing up in like diet, culture […] if you're this weight then you're obviously unhealthy and everything is horrible about you."*<br><br>*"during that time I was under like quite a bit of stress. Especially with moving like I moved. it made it very hard for me to kind of stick to the diet."*

© 2026 Gao M et al. *JAMA Psychiatry*.

		Adverse Side-Effects		*"I would say I suffered and it was very brutal on my body."*<br><br>*"I think I took about four weeks to actually get into ketosis properly and it was very grueling"*
**Long-Term Sustainability**	What dietary changes have participants continued	Small, incremental changes	Some participants reported incorporating small changes after transition off the formal ketogenic diet intervention to maintain healthier eating habits. These included increased consumption of vegetables and protein intake, smaller portion sizes, and reduced sugar and processed foods, switching to almond milk and sweeteners for coffees and purchasing cookware (e.g., spiralizer) to make keto-friendly meals. However, many participants reported they stopped the ketogenic diet immediately after the 6-week program ended.	*"I've stopped eating ready meals and I generally now look at what I'm eating and try and avoid anything that's over processed."*<br><br>*"I've had a couple of meals out since or you know, I was at a wedding at the weekend and obviously some of the stuff on the menu wasn't necessarily compliant. But yeah, generally speaking, I've stuck to the same foods, which is, which is good."*<br><br>*"I brought spiraliser so I can make my own like courgetti spaghetti and things."*<br><br>*"Now I have multiple cups of coffee during the day and I did like change to light almond milk and sweeteners and that was OK, but I noticed."*<br><br>*"I still eat a lot of broccoli, cauliflower, chicken breast and all of that, all the stuff that was on the diet, I still eat, but I also do sometimes I do have things like. Biscuits."*<br><br>*"I don't overeat carbs still, you know, position wise it's still very small for me [...] I am much better eating like, you know, the protein and the fats than actually having a bigger portion of carbs"*
	Challenges to continuing	Restrictive Nature of Diet	Participants shared a variety of reasons for discontinuing the ketogenic diet after the six-week intervention period, highlighting challenges related to financial cost of purchasing the required foods, the immediate end of the structured support and meals, and difficulties with intense cravings and reintroduction of non-ketogenic foods.<br><br>Some participants expressed a strong desire to deliberately reward themselves with indulgent foods, such as cake, chocolate, bread, and pasta after abstaining from these foods on the KD. Participants noted that the reintroduction of carbohydrates and sugar was associated with negative mental and physical consequences with some	*"we don't have a lot of money and you know, buying all the meats and things and stuff [...] it'd be expensive for me because we're on benefits."*<br><br>*"It's a bit expensive, to be honest, to follow this diet"*<br><br>*"it's an all or nothing diet. Then you know if you have a bad day and then you decide to have like a binge or whatever, and then then that's kind of it"*

© 2026 Gao M et al. *JAMA Psychiatry*.

			recognizing a pattern of cravings. For a few, high-sugar food consumption cravings made adherence to the ketogenic diet unsustainable and some participants experienced re-emergence of other cravings and habits, which further complicated maintaining the ketogenic diet over the long term.<br><br>Several participants noted that personal events, such as birthdays or weekends away, disrupted their ability to continue with the ketogenic diet. While some expressed initial enthusiasm and intention to continue with the ketogenic after the six-week intervention ended, they found it difficult to sustain without weekly dietitian support, monitoring and tracking, and prepared meals.<br><br>Overall, while many participants recognized some benefits from following the ketogenic diet, most discontinued after the intervention period citing financial constraints, social events, sugar cravings, and the restrictive nature of the diet as key barriers.	*"when the delivery stops, like immediately afterwards I was like, OK, Yeah, I'm carrying on with this. Then, unfortunately, I sort of lapsed"*<br><br>*"the time and the sort of the energy investment of then trying to manage your diet by yourself"*<br><br>*"I wasn't eating the stuff I enjoy. I felt like I've lost the freedom."*
		Cravings		*"After my six weeks have finished I was thinking, oh, I can't wait for my weekend away because I'll be able to take a box of chocolates. I'd actually frozen a piece of a 21st birthday cake to take with me for the end of it."*<br><br>*"I've been buying completely unhealthy additional food. I think my meals haven't been too bad, but I've been buying chocolate bars and snacking on bits of cake and come I feel like the sugar. My mental health took a massive decline. Once I started eating sugar again, I really could feel it really could feel it."*<br><br>*"I had given up smoking and I noticed that during the keto diet and I started to crave cigarettes more and I went back to smoking."*<br><br>*"I've gone from feeling quite positive, quite upbeat, and then I had this weekend where I ate so much sugar compared to what I would have had, and all of the things you would generally do as part of your normal."*<br><br>*"I've stopped eating keto and then I've let the sugar get a grip of me and I've been buying chocolate bars."*<br><br>*"after you haven't had sugar and carbs for six weeks or maybe a little bit longer, you just kind of want to reward yourself with something. Buy pasta or I don't know, something that guilty pleasure."*

© 2026 Gao M et al. *JAMA Psychiatry*.

		External factors		*"as soon as my six weeks finished the timing wasn't great for my personal life, I suppose because I had a weekend away booked."*<br><br>*"I would do better if my job were less stressful. You know, if I could have more time to think about [it], you know, and exercise and all of that. But because of my job and my circumstances, I really just couldn't [continue]."*<br><br>*"it was not going to make me any happier and it's probably going to give me a heart attack in the end so that those were the reasons."*
**Mental and Physical Outcomes**	Impact on physical health	Positive	In terms of health outcomes, the experience was highly individualized, with some participants finding the diet transformative and others reporting minimal or mixed effects. The findings highlight the variability in outcomes based on personal circumstances and individual responses to the diet.<br><br>Some participants noted significant weight loss, reduced bloating, and improved menstrual symptoms, contributing to an overall perception of enhanced physical health outcomes.<br><br>Reported benefits were specific to the 6-week intervention period, with very few reporting continued benefits beyond stopping the intervention.<br><br>Challenges were reported, particularly with digestive health. Post-intervention, participants often experienced a return to pre-study conditions, though many acknowledged greater awareness of their dietary choices and health habits.	*"the joint pain that I previously had, because I also have hypermobility syndrome and that severely reduced, which was good."*<br><br>*"my tonsils severely reduced in size."*<br><br>*"it changed my period for the better, like I didn't get as to be a cramps or heavy bleeding as I have them previously."*<br><br>*"I would say that the thing that I noticed in diet was I was less bloated, my tummy was more, felt more healthy"*
		Negative		*"on my physical health, like 'cause, I was eating so much dairy, physically it wasn't not good for me […] my bowel habits were very different. So that was a bit of a pain"*
		Neutral/No Change		*"physical side, it was quite good cause I didn't necessarily feel hungry and I didn't necessarily feel full if that makes sense."*
	Impact on symptoms of depression	Positive	Mental health outcomes varied across participants. Many described a sense of improved mental clarity and emotional stability, with some reporting they felt better equipped to handle life's challenges and process emotions more rationally.<br><br>A notable outcome was the reduction in antidepressant use for some, with participants attributing their improved mood and energy levels to the diet.	*"I felt more mentally equipped to deal with the challenges of life that would probably normally have really upset me. I'd have dwelled on […] I felt I was more able to process them in a more rational manner."*<br><br>*"I finally start realizing my desires that I want something. And you know, when you're in long term depression you're forgetting these kind of feelings that you have joy of life or you're happy to see the sun"*<br><br>*"two weeks before I went on started the trial, my GP had raised my antidepressants and within the two weeks of starting the keto diet, I felt like I didn't need the increase"*

© 2026 Gao M et al. *JAMA Psychiatry*.

				"I told the doctor what I'd been doing. And actually since then I have come off my medication."<br><br>*"I could probably say it's been a little bit more positive rather than it's the end of the world and you know it's been doom and gloom all the time."*<br><br>*"changing my diet did so much more than relying on a little pill every day"*<br><br>*"It felt that it really suited me. It felt, oh, my God, I haven't felt this good for years"*<br><br>*"I did feel a little bit more positive and I was looking at what was going on in my life around me and nothing really had changed."*<br><br>*"I was out with my two daughters and I can't remember where we were, what we were doing. But I remember thinking this is pretty good and I remember then thinking to myself, oh, I wonder if that's to do with this diet I'm on […] and that's not a feeling I've had and a long time, probably ever. it was nice to experience that but it didn't happen very often."*<br><br>*"something has had an effect, so even if it is that small."*<br><br>*"It definitely improved my mood and we then the first two weeks I felt I was starting to feel so much better and I had so much energy I didn't know what to do with it."*<br><br>*"I stopped crying. I was crying all the time. And after that, about week three, I realized that if someone was talking to me or saying certain things, I wasn't crying."*
		Negative	For others, however, mood improvements were less pronounced, with some participants reporting persistent low mood or questioning whether changes were due to the diet or external factors, such as support systems or improved life circumstances.	*"so my mood it was a bit lower cause I took comfort in my own food which I wasn't able to have so"*<br><br>*"I was just so stressed out about everything else outside of the diet that I didn't see a difference in my mood."*<br><br>*"Not really, I've just got that persistent depression. But it just it just stays. […] it just stayed the same. So it was just the same low mood, feeling bad."*<br><br>*"sometimes for some people, it takes longer to be on it to notice the difference. And maybe for me, because everyone's body works differently. Six weeks wasn't long enough. Maybe."*

© 2026 Gao M et al. *JAMA Psychiatry*.

				"I didn't feel any positive effect from a mood perspective during the study, which is, you know it is what it is and therefore there wasn't really a motivation for me to continue with a keto diet"<br><br>*"there were still kind of dips in in mental health that definitely, you know, it didn't cut out the sort of the negative thinking and things."*
		Neutral/No Change	A few participants reported no noticeable mental health benefits, suggesting that the impact of the diet may vary depending on individual contexts and timelines.	*"It wasn't that I wasn't feeling happy, I wasn't feeling anything other than the fact that everything, everything seemed OK. I remember thinking to myself, I've never felt like that [...] something changed"*<br><br>*"Overall, I think it was up to a degree but again can I be sure that that was the diet or was [...] there was a support team and people to speak to, somebody that's interested in how you're doing and the fact that you know it takes the stress out of trying to prepare meals and things for yourself?"*<br><br>*"I had a few personal circumstances which also improved, so my mood was generally better, but I don't know if it's because of the diet or if it's because of the other circumstances."*<br><br>*"so my easy conclusion is, oh, it didn't help at all. I can't really pin it to the study as such or to the food or anything else, because any depression goes in waves like that anyway."*
	Impact on other aspects of well-being	Positive Experiences	Participants reported additional impacts on well-being including reduced self-doubt, experiencing joy or neutrality instead of persistent sadness. Functionality in daily life also saw enhancements, with participants becoming more consistent in maintaining hygiene, following routines, and engaging in life such as work, social outings, and physical activity.<br><br>Improved sleep was another commonly cited benefit, contributing to overall wellbeing, although this was not consistent across participants.<br><br>A few participants reported no lasting or significant changes.	*"I have to juggle multiple things. I work. I'm a single parent. I've got a 6 year old and a 20 year old. I'm doing a master's degree. I tutor. I juggle multiple things so life is very stressful and muddled at the best of times. I think I did notice. Yeah, that it gave me mental clarity [...] like mental robustness."*<br><br>*"I'm functional now. I would say like I take shower most of the time on time. I'm taking care of the hygiene. I can go out. I do follow my work schedule as well."*<br><br>*"I wanted to walk more. I wanted to do more things."*<br><br>*"My sleep really improved and before I wasn't sleeping much at all. But during the keto diet I was going to bed and sleeping solidly."*<br><br>*"my family commented of like, yeah, you haven't had any kind of meltdown. Not having this like crippling self doubt all the time was fantastic."*
		Challenges		*"I found it hard to get to sleep and stay asleep. I think there's one the reasons I started to reintroduce carbs. I just couldn't deal with the sleep deprivation anymore"*

		Neutral		*"I didn't really notice anything overly around the concentration or energy or anything like that."*<br><br>*"Do I feel better for having lost weight? Yeah, I do. I feel better because I can exercise more and I feel better because of various things. So in a sense, there's a positive outcome of having done it that I don't think is a direct result of [Keto] or being in [Keto], being in ketosis."*
**Future improvements**	Enhanced dietary materials and advice	Clear and specific instructions	Participants shared several recommendations to improve future ketogenic diet trials, primarily focused on accessibility, structured guidance and support beyond the intervention period, and enhancements to study protocols.<br><br>In terms of dietary support and materials, participants suggested providing clearer educational materials that included culturally diverse meal alternatives or enhancements, practical advice such as easy-to-follow grocery lists, cheat sheets and keto-friendly recipes, including alternative options for cravings. Participants also suggested enhanced educational materials that explained how the diet affects the brain and body to help enhance motivation.<br><br>Participants also emphasized the importance of accessible and personalized food options, such as pre-prepared meals that tailored to cultural preferences, electrolytes and mineral support to address symptoms, and cold food options when microwave or refrigeration options were unavailable. Budgetary support for customized meal plans or groceries to cook one's own meals was also another suggestion given the variation in palatability of the provided meals and financial constraints mentioned.<br><br>Participants expressed a desire for greater support to address emotional and mental health challenges during the diet to help with adherence. Several mentioned dips in mood, anxiety, and other emotional struggles and suggested having dedicated time to speak to that as a way to help with diet adherence.<br><br>Digital tracking tools, expanded recipe options, and flexible program modifications were suggested to improve adherence and participant satisfaction.<br><br>Study protocol recommendations included the possibility of extending the duration of the intervention since many participants felt six-weeks was not long enough for the body to fully adapt to the ketogenic diet or to notice lasting changes. Streamlining study assessments into a more user-friendly digital method instead of separate website links to complete various measurements may eliminate confusion about completing study assessments. The exploration of digital tracking tools, especially after the intervention period was mentioned as a suggestion to improve adherence to the diet.<br><br>Practicality, personalization, emotional support, and sufficient time were important factors to improve adherence and outcomes in future ketogenic diet programs.	*"you could have had some suggestions of like things you can easily buy at the supermarket or things like that."*<br><br>*"I think I think a lot more like specificity as in a bit more black and white.[...] I personally would find like you know, say like a cheat sheet or something and that would be beneficial."*<br><br>*"it would have been nice if I'd had recipes I could have thought, well OK, if I'm going to deviate, I'll make something that is still OK."*<br><br>*"this is what we're thinking happens with your brain with this kind of diet and how these different parts impact your brain. I think that might have helped me stick to it a bit more."*
	Improved meals and snacks	Variety<br><br>Cultural Considerations<br><br>Symptom Management<br><br>Program Modification<br><br>Choice and Autonomy		*"I feel like if there were more options from the beginning it may be a more present journey I guess."*<br><br>*"I went out and I bought some electrolyte and supplementary staff that other people are that of, my friends who had similar things recommended and that helped."*<br><br>*"So I was putting like Curry powder in my soup, putting garlic powder into stuff. And just like, you know, putting fresh herbs or salad or, you know, stuff with it."*<br><br>*"are there halal versions of pre prepared keto meals and such like so you know to broaden it out"*<br><br>*"would have been better if there were like some these kind of option cold food options."*<br><br>*"I would have preferred to have some sort of a budget where I can go grocery shopping and I could have gotten the list of things."*<br><br>*"maybe participants can have an option either go for customized meals or get the budget and make their own meal."*

© 2026 Gao M et al. *JAMA Psychiatry*.

	Emotional support	Understand Mental Health Context		*"I think having a different kind of support. I felt like that didn't necessarily address the emotional or anxiety issues that I was going through [...] I couldn't really talk to anybody about that. And I think that made it a lot harder to stick to the diet"*<br><br>*"I think for those of mental health, it could really go either way of whether someone could slip into being obsessive of a can't eat that because it's this or stuff like that."*
	Study Components	Study Length<br><br>Study Assessments<br><br>Tracking and Monitoring Tools		*"So maybe actually seeing whether or not a longer period other than longer than six weeks may have had an impact."*<br><br>*"Maybe to make it a bit longer, that's the one thing, because I believe that six weeks is not enough."*<br><br>*"it takes time to body to change the way of how they just things. And you know, to get used to new diet as well. So I think it have to be a bit longer, that's one thing."*

© 2026 Gao M et al. *JAMA Psychiatry*.

Reference

1. Centre NBR. You said we did – A case study from Preventing Multiple Morbidities Theme. 2024. https://oxfordhealthbrc.nihr.ac.uk/you-said-we-did-a-case-study-from-preventing-multiple-morbidities/.
2. Gao M, Kirk M, Lash E, et al. Evaluating the efficacy and mechanisms of a ketogenic diet as adjunctive treatment for people with treatment-resistant depression: A protocol for a randomised controlled trial. *Journal of Psychiatric Research* 2024; **174**: 230-6.

© 2026 Gao M et al. *JAMA Psychiatry*.
