# Supplementary material for: A Ketogenic Diet for Treatment-Resistant Depression: A Randomized Clinical Trial
Source: JAMA Psychiatry. 2026 Feb 4;83(4):331–40. doi: 10.1001/jamapsychiatry.2025.4431 (PMC12874075; doi:10.1001/jamapsychiatry.2025.4431)
Supplement: Supplement 3. — Data Sharing Statement [file jamapsychiatry-e254431-s003.pdf]

Data Sharing Statement

Gao. A Ketogenic Diet for Treatment-Resistant Depression. *JAMA Psychiatry*. Published February 04, 2026. doi:10.1001/jamapsychiatry.2025.4431

Data
Additional Information: ClinicalTrials.gov Identifier: NCT06091163
Data available: No
